# The effects of flipped classrooms to improve learning outcomes in undergraduate health professional education: A systematic review

**DOI:** 10.1002/cl2.1339

**Published:** 2023-07-07

**Authors:** Cho Naing, Maxine A. Whittaker, Htar Htar Aung, Dinesh Kumar Chellappan, Amy Riegelman

**Affiliations:** ^1^ Division of Tropical Health and Medicine James Cook University Townsville Australia; ^2^ Department of Human Biology, School of Medicine International Medical University Kuala Lumpur Malaysia; ^3^ Department of Life Sciences, School of Pharmacy International Medical University Kuala Lumpur Malaysia; ^4^ University Libraries University of Minnesota Minneapolis Minnesota USA

## Abstract

**Background:**

The ‘flipped classroom’ approach is an innovative approach in educational delivery systems. In a typical flipped class model, work that is typically done as homework in the didactic model is interactively undertaken in the class with the guidance of the teacher, whereas listening to a lecture or watching course‐related videos is undertaken at home. The essence of a flipped classroom is that the activities carried out during traditional class time and self‐study time are reversed or ‘flipped’.

**Objectives:**

The primary objectives of this review were to assess the effectiveness of the flipped classroom intervention for undergraduate health professional students on their academic performance, and their course satisfaction.

**Search Methods:**

We identified relevant studies by searching MEDLINE (Ovid), APA PsycINFO, Education Resources Information Center (ERIC) as well as several more electronic databases, registries, search engines, websites, and online directories. The last search update was performed in April 2022.

**Selection Criteria:**

Included studies had to meet the following criteria: **Participants**: Undergraduate health professional students, regardless of the type of healthcare streams (e.g., medicine, pharmacy), duration of the learning activity, or the country of study. **Intervention**: We included any educational intervention that included the flipped classroom as a teaching and learning tool in undergraduate programs, regardless of the type of healthcare streams (e.g., medicine, pharmacy). We also included studies that aimed to improve student learning and/or student satisfaction if they included the flipped classroom for undergraduate students. We excluded studies on standard lectures and subsequent tutorial formats. We also excluded studies on flipped classroom methods, which did not belong to the health professional education(HPE) sector (e.g., engineering, economics). **Outcomes**: The included studies used primary outcomes such as academic performance as judged by final examination grades/scores or other formal assessment methods at the immediate post‐test, as well as student satisfaction with the method of learning. **Study design**: We included randomised controlled trials (RCTs), quasi‐experimental studies (QES), and two‐group comparison designs. Although we had planned to include cluster‐level RCTs, natural experiments, and regression discontinuity designs, these were not available. We did not include qualitative research.

**Data Collection and Analysis:**

Two members of the review team independently screened the search results to assess articles for their eligibility for inclusion. The screening involved an initial screening of the title and abstracts, and subsequently, the full text of selected articles. Discrepancies between the two investigators were settled through discussion or consultation with a third author. Two members of the review team then extracted the descriptions and data from the included studies.

**Main Results:**

We found 5873 potentially relevant records, of which we screened 118 of them in full text, and included 45 studies (11 RCTs, 19 QES, and 15 two‐group observational studies) that met the inclusion criteria. Some studies assessed more than one outcome. We included 44 studies on academic performance and eight studies on students' satisfaction outcomes in the meta‐analysis. The main reasons for excluding studies were that they had not implemented a flipped class approach or the participants were not undergraduate students in health professional education. A total of 8426 undergraduate students were included in 45 studies that were identified for this analysis. The majority of the studies were conducted by students from medical schools (53.3%, 24/45), nursing schools (17.8%, 8/45), pharmacy schools (15.6%, 7/45). medical, nursing, and dentistry schools (2.2%, 1/45), and other health professional education programs (11.1%, 5/45). Among these 45 studies identified, 16 (35.6%) were conducted in the United States, six studies in China, four studies in Taiwan, three in India, two studies each in Australia and Canada, followed by nine single studies from Brazil, German, Iran, Norway, South Korea, Spain, the United Kingdom, Saudi Arabia, and Turkey. Based on overall average effect sizes, there was better academic performance in the flipped class method of learning compared to traditional class learning (standardised mean difference [SMD] = 0.57, 95% confidence interval [CI] = 0.25 to 0.90, *τ* ^2^: 1.16; *I* ^2^: 98%; *p* < 0.00001, 44 studies, *n* = 7813). In a sensitivity analysis that excluded eleven studies with imputed data from the original analysis of 44 studies, academic performance in the flipped class method of learning was better than traditional class learning (SMD = 0.54, 95% CI = 0.24 to 0.85, *τ* ^2^: 0.76; *I* ^2^: 97%; *p* < 0.00001, 33 studies, *n* = 5924); all being low certainty of evidence. Overall, student satisfaction with flipped class learning was positive compared to traditional class learning (SMD = 0.48, 95% CI = 0.15 to 0.82, *τ* ^2^: 0.19, *I* ^2^:89%, *p* < 0.00001, 8 studies *n* = 1696); all being low certainty of evidence.

**Authors' Conclusions:**

In this review, we aimed to find evidence of the flipped classroom intervention's effectiveness for undergraduate health professional students. We found only a few RCTs, and the risk of bias in the included non‐randomised studies was high. Overall, implementing flipped classes may improve academic performance, and may support student satisfaction in undergraduate health professional programs. However, the certainty of evidence was low for both academic performance and students' satisfaction with the flipped method of learning compared to the traditional class learning. Future well‐designed sufficiently powered RCTs with low risk of bias that report according to the CONSORT guidelines are needed.

## 1 PLAIN LANGUAGE SUMMARY

### 1.1 Flipped classrooms may improve academic performance and satisfaction of undergraduate health professional students

Flipped classroom learning appears to improve academic performance and the evidence suggests student satisfaction with the innovative learning method, but the certainty of the evidence was low.

### 1.2 What is the review about?

Students face several challenges when learning through traditional teaching settings. They need to accumulate huge amounts of factual knowledge from the courses, and to keep up‐to‐date with the prolific growth in health knowledge.

Lack of awareness about digital technologies and non‐exposure to digital‐friendly environments have made learning even more challenging. Therefore, an innovative approach to the education delivery system is needed.

A flipped class includes two elements of education: a recorded lecture (off‐campus learning as homework) and an active learning session (on‐campus learning). Pre‐recorded lectures are provided to the students as homework and as an aid to learning which is then interactively discussed later on campus.

This review aims to explore whether there is empirical evidence that supports this method of learning for undergraduate health professional students. Do flipped classrooms improve academic performance and are students satisfied with the flipped class learning method?

**What is the aim of this review?**

This Campbell systematic review examines the effects of flipped class teaching compared to the traditional teaching class. The review summarizes evidence from 45 studies, including 11 randomised controlled trials.

### 1.3 What studies are included?

This review includes studies that have evaluated the effect of flipped classes compared to traditional classes on the academic performance and course satisfaction of health professional undergraduate students.

Forty‐five studies were identified, involving 8,426 undergraduate students in medicine, pharmacy, nursing and other health professional courses.

Of these, 44 studies involving 7,813 undergraduate students examined the outcome of academic performance, measured by examination scores/final grade). Only eight studies, involving 1,696 undergraduate students, examined the outcome of students' satisfaction.

Studies spanned the period 2013 to 2021. Sixteen studies were conducted in the USA, and only three studies were from lower‐middle‐income countries, including India. All the studies had important methodological weaknesses.

### 1.4 Does the flipped class method of learning improve students' academic performance?

Yes, low certainty of evidence shows an overall improvement in academic performance when flipped classroom interventions were implemented compared to traditional lecture‐based classes.

### 1.5 Are students satisfied with flipped class learning?

Yes, low certainty of evidence shows that students' satisfaction with the flipped classroom method of learning is positive. Therefore, further research may change the estimate in either direction (that is, a larger difference, or no difference, in satisfaction).

### 1.6 What do the findings in this review mean?

The review shows that flipped classroom learning may improve academic performance and satisfaction of undergraduate health professional students. Well‐designed studies with larger samples that rigorously evaluate the outcomes are needed.

### 1.7 How up‐to‐date is this review?

The literature searches were last conducted in April 2022.

## 2 BACKGROUND

### 2.1 Description of the condition

In a traditional educational experience, a teacher stands in front of the classroom and delivers a lecture to a group of students, who sit in rows, quietly listening to the lecture and taking notes. At the end of the lecture, students are given homework or an assignment to be completed outside the classroom environment. This characterises the principle of ‘sage‐on‐the stage’ and is synonymous with the present‐day *mode* of *teacher‐centred learning*. This is also referred to as the transmittal model (King, 1993), which assumes that the students are *passive note‐takers, receivers of the content or accumulators of factoids* (Morrison, 2014). In such a scenario, the teacher usually does not have the required freedom of time to interact with the students individually during the class (Hamdan, 2013), thus neglecting those students who do not understand the lecture. The traditional didactic way of teaching is primarily unidirectional and typically witnesses limited interactions between the source of knowledge (teacher) and the passive recipients (students).

One of the main challenges faced by lecturers is the overload of academic content that needs to be taught in a relatively short time. Equally challenging is the situation faced by the students who lose interest or motivation to learn within the stipulated time (Prober, 2013). The traditional way of teaching, therefore, discourages the students from active learning and critical thinking. There is also increasing pressure from accrediting institutions, who demand evidence for ‘the ability to communicate effectively’, ‘the ability to identify, formulate and solve problems’, and ‘the ability to function as multidisciplinary teams’ (Bishop, 2013). There exists a large body of research that suggests the crucial need to transform the current pedagogical strategies that may be required to enhance active learning in a more effective way (Al Faris, 2013). Synthesis of research on the effectiveness of lectures shows that lectures are neither an effective method for teaching nor developing values or for personal development, and they may only be effective for the sole goal of transmitting information (Bligh, 2000). Considering these observations, it is essential to explore newer methods that have the potential to maximise the use of classroom time and transform the classroom into a platform for effective teacher‐student interactions and critical thinking (Rui, 2017).

Numerous factors have cumulatively led to several challenges for traditional teaching in health professional education including the availability of digital technologies, digitally‐empowered learners, the prolific expansion of courses, the amount of factual knowledge that has been accumulated in the courses, prolific growth of health knowledge, advancements in healthcare disciplines, and investments into the scholarship of teaching and learning. Technological advancements and cutting‐edge research have enabled the development of newer delivery systems encompassing active learning in HPE. Studies have reported that active participation is an effective method to improve learning and understanding (Freeman, 2014; McCoy, 2015). Thus, to enhance interaction during their learning process there are effective educational strategies, which promote active learning in traditional lectures by engaging students in doing things, and encouraging them to think about what they are doing.

There are various modifications, which can be incorporated into traditional lectures that enable active learning in the classroom, for instance; (1) the ‘feedback lecture’, which consists of two mini‐lectures separated by a small‐group study session built around a study guide, and (2) the ‘guided lecture’, where students listen to a 20‐ to 30‐min presentation without taking notes, followed by their writing for 5 min on what they remember, and spending the remainder of the class duration in small groups for clarification and elaboration on the study material (Ellis, 2010; Johnson, 2013). Moreover, there are other active learning pedagogies, which include visual‐based instructions (Johnson, 2016), small group problem‐based learning, cooperative learning, debates, drama, role‐playing and simulation, and peer teaching.

One innovative approach in the education delivery system is the ‘flipped classroom’, an educational technique that consists of two parts, interactive group learning activities inside the classroom and direct personal computer‐based individual instruction outside the classroom (Bishop, 2013). In a typical flipped class model, work was typically done as homework in the didactic model (e.g., problem‐solving, essay writing) is interactively undertaken in the class with the guidance of the teacher, whereas listening to a lecture or watching course‐related videos is undertaken at home. Hence, the term *flipped* or *inverted classroom* is used (Herreid, 2013). The essence of a flipped classroom is that the activities carried out during traditional class time and self‐study time are reversed or ‘flipped’ (Veeramani, 2015). Pedagogical approaches to undergraduate teaching have improved over the years as the Scholarship of Teaching and Learning has provided relevant evidence of what contributes to improving outcomes. However, educational delivery approaches have shown little change in many disciplines and have remained the same for the majority of the sectors (Van Vliet, 2015).

### 2.2 Description of the intervention

The flipped class is a flexible tool by itself and can be tailored according to the outcomes that are predesigned (Tetreault, 2013). Historically, the concept of flipped classroom started in the early 1990s. General Sylvanus Thayer created a system at West Point in the USA, where a set of learning materials was given to engineering students so that they obtained the core content before attending class. The classroom space was then used for critical thinking and group problem solving (Musallam, 2011). Many credited the rejuvenation of this idea with the development of, and increased access to, educational technologies (Moffett, 2015). For instance, the School of Business at the University of Miami proposed an ‘inverted classroom’, which had events that traditionally took place inside the classroom now taking place outside the classroom and vice versa (Lage, 2000). In 2000, a conference paper entitled ‘The Classroom Flip’ was presented by J. Wesley Baker and the phrase ‘flipping the classroom’ was coined. Baker described how flipping the classroom could allow the trainer to become the ‘guide on the side’ rather than the ‘sage on the stage’ (Baker, 2000).

In a sense, this reversal also flips Bloom's revised taxonomy because the lower level of cognitive work/knowledge acquisition is done by the students, while educators work interactively with the students to develop the higher forms of cognition. To date, this approach has attracted a large amount of attention in the health professional education and a subsequent surge of literature.

Fundamentally, a flipped classroom encompasses two established elements of education, the recorded lecture (off‐campus learning) and active learning (on‐campus learning). Pre‐recorded lectures are provided to the students as homework, as an aid to learning. Homework is important because it is a time where students can share their learning progress with their family, reflect on their learning, and review the material as well as the educator's feedback (Fulton, 2012). The key characteristics of a flipped classroom compared to a traditional classroom and other existing teaching methods are summarised in Table 1.

**Table 1:** Synopsis of the comparison between flipped classroom and other teaching modes.

Description	Traditional classroom	Distant education	Flipped classroom
Teacher centred	√	√	‐
Student centred	‐	‐	√
Passive learning environment	√	√	‐
Active learning environment	√	√	√
Face‐to‐face lecture	√	‐	‐
First phase (lecture)	In the classroom	At home	At home
Second phase (active activities^a^)	At home	At home	In the classroom

a Examples are group discussions, case studies, feedback sessions, problem solving activities, presentations and polling.

It has been highlighted that the flipped classroom fits into the broader context of blended learning (Tetreault, 2013). Blended learning as defined by Staker is, ‘a formal education program in which a student learns at least in part through online delivery of content and instruction with some element of student control over time, place, path and/or pace and at least in part at a supervised brick‐and‐mortar location away from home’ (Staker, 2012, p. 3). The flipped classroom consists of educational programs or classes as a means of formal learning, and interactive online tools such as educational videos, quizzes/games as mechanisms of informal learning. The flipped classroom approach is connected between what the students learn online (e.g., video lecture) and what they learn face‐to‐face (e.g., in‐class active case study), and vice versa, which is a common feature of blended learning (Tetreault, 2013). In principle, the flipped classroom assigns relatively low‐level cognitive learning capabilities such as memorising and understanding, which is accomplished outside of the classroom whereas, teaching in class is accomplished mostly through teacher‐student interactions and cooperation between peers, thereby stimulating the students' intellectual potential (Rui, 2017). The option to view video lectures (as an example) outside of the classroom has beneficial effects for the learners as they can replay the videos as many times as needed to better understand the key concepts at their own pace. Furthermore, this allows effective comprehension and analysis of the topics covered to each student's satisfaction, whereas this might not be possible in the context of conventional teacher‐centred teaching. This is an important pedagogical consideration for international students for whom English is their second language (Moraros, 2015). From the teacher's perspective, a flipped classroom setting makes it easier to engage students and empower them as active participants of their learning.

### 2.3 How the intervention might work

Several (general) theoretical frameworks are available to inform our understanding of the use of technology in the specific context of a flipped classroom. Two of these include the Technology Acceptance Model (TAM) (David, 1989) and the Unified Theory of Acceptance and Use of Technology (UTAUT) (Venkatesh, 2003). These theoretical frameworks provide guidance for the analysis and identification of relevant outcomes. We will describe how the theoretical frameworks can help us understand the pathway through which the learning outcomes can lead to an improved academic performance.

TAM includes two theoretical variables (constructs): (i) perceived usefulness and (ii) perceived ease of use. These variables are described as ‘the degree to which a person believes that using a particular system would enhance his or her job performance’ and ‘the degree to which a person believes that using a particular system would be free of effort’, respectively (David, 1989, p. 320). The first theoretical variable relies on students' prior knowledge, gained from the pre‐class video lecture (for example), in enhancing their understanding (and overall learning performance) of in‐class activities such as problem‐solving. The second theoretical variable suggests that people are more likely to adopt a flipped classroom if it is more user‐friendly than traditional teaching methods.

The goal of the UTAUT model is to explain the intentions of a user to employ a given information system and the subsequent behaviour of the user. The model is based on four primary variables: (1) performance expectancy, (2) effort expectancy, (3) social influence and (4) facilitating conditions (Venkatesh, 2003, p. 447). The first three variables reflect the motivation of the users (i.e., students). The fourth variable reflects the physical environment (i.e., the learning items necessary in class). These materials could be a video, an interactive presentation, a questionnaire, or sometimes a recorded audio presentation. Concerning these theoretical variables, if a flipped classroom is user‐friendly and the academic environment facilitates their learning, then it should promote students’ engagement, interactions, and cooperation in learning, which will further improve their performance.

There are potential advantages of a flipped classroom, including increased opportunities to provide individualised education to learners (Johnson, 2013; Kachka, 2012), increased student engagement with course material (Gross, 2015), and increased educator‐student interaction, compared to a ‘performing’ lecture. The Kirkpatrick model of educational outcomes (Issenberg, 2005; Kirkpatrick, 2006) comprises ‘learners’ reaction’ (to the educational experience); learning (modification of attitudes/perceptions and the acquisition of knowledge and skills); behaviour (self‐reported changes in practice and observed changes in practice, including new leadership positions); and results (which refers to change at the level of the organisation). For instance, with regard to the ‘results’ outcome, the flipped classroom allows the teacher to gain advanced, real‐time insight into how students learn, and quickly identify and address the curriculum content in an efficient way, the content which they originally found most challenging. This insight can be used to better inform decisions concerning effective curriculum organisation, structure, and delivery of future classes.

The success of a flipped‐classroom approach relies on several assumptions. Stimulation of students' interest in learning and guided self‐study (Moraros, 2015), primarily depends on the opportunities to actively engage students in self‐directed learning and encourage progressive improvement (Bergmann, 2012; Moraros, 2015) in assessment performances. Thus, a flipped class will not support effective learning if students fail to engage with the assigned pre‐class or in‐class activities (Kachka, 2012), for reasons which might include poorly designed educational materials (e.g., long, poor audio quality) or students feeling ‘lost’ (Moffett, 2015). As such, many contextual and structural factors may influence flipped classroom learning including resources (inputs to the program), activities (aspects of implementation), outputs (observable products of the completed activities), and outcomes (effects or impacts within various time frames) as depicted in the conceptual framework (Supporting Information: Appendix 1).

### 2.4 Why it is important to do this review

There are several individual studies, which have evaluated flipped classrooms in medical education, allied health education, and health science education, using a pre‐and post‐test design or comparative designs to explore how learning outcomes may be improved. Some studies showed positive outcomes with flipped classrooms (Galway, 2014; Van Vliet, 2015), while others showed the opposite (Whillier, 2015). For instance, a study on integrated flipped lectures with online teaching techniques assessed the learning experiences and participation through active learning. The reported findings suggested that the students in the integrated flipped‐online lectures had achieved an increase in active learning components compared to the group that was put in a didactic model (Galway, 2014). It is important to consider the factors that could have contributed to this difference. As an example, to achieve a balance in a safe learning environment (to be free from discomfort and fear) between the two groups of students, a comparison of the personality traits of the students in each group needs to be considered. On the other hand, another individual study, which assessed the effectiveness of flipped classrooms in ophthalmology clerkship reported that the students in flipped classrooms had more burden and pressure in preparing for the pre‐class compared with the students in the lecturer‐based classroom group. However, these published individual studies varied in design, sample size, and outcome measures. It is unclear if these findings could be generalised to other health professional educations. A non‐Campbell systematic review of the flipped classroom model reported how the flipped classroom has been applied in nursing education and the achieved outcomes associated with such teaching (Betihavas, 2016). Due to the focus on a particular educational context (i.e., nursing or ophthalmology), the generalisability of their findings to other courses in undergraduate health professional education is uncertain. Another non‐Campbell collaborative systematic review, consisting of 82 studies reported on the effectiveness of flipped classrooms in medical education where a pooled estimate of a subset of six experimental studies showed generally positive perceptions of the students to the flipped classroom. However, there were no significant changes in their knowledge and skills (Cohen's *d* = −0.27 to 1.21, median: 0.08) (Chen, 2017). These systematic reviews, which focused on a particular area (either nursing education or medical education) had a limited number of included studies, considerable variation in study design, a lack of methodological quality assessment of the included studies, and the quality of evidence reported by these systematic reviews was poor.

A systematic review, which combines the results of interventions, using flipped classrooms compared with alternative learning or traditional learning, would help inform the development and implementation of successful flipped classrooms amongst health professionals. The current review also aims to serve as a reference document for decision‐makers to support evidence‐based approaches to the flipped classroom in health professional education.

## 3 OBJECTIVES

The primary objective of this systematic review was to assess the effectiveness of flipped classroom interventions for undergraduate health professional students on academic performance, and course satisfaction.

The secondary objectives were to explore:

- The influence of context in the design, delivery, and outcomes of flipped classroom interventions in undergraduate health professional education;
- The barriers and facilitators of flipped classroom learning effectiveness for undergraduate health professional students.

Specifically, this review was designed to answer the following research questions:

**Primary research question**

- What are the effects of flipped classroom learning on undergraduate health professional students' academic performance?
- What are the effects of flipped classroom learning on undergraduate health professional students' course satisfaction?

**Secondary research questions**

- Do any moderator variables affect the effectiveness of flipped classroom learning on academic performance outcomes?

Moderators such as study design, student‐related factors including the amount of out‐of‐class preparation time, classroom availability, limited high‐speed Internet access for rural and remote students, quality of interactive tools, and faculty‐related factors such as faculty members’ preference for a more didactic approach.

## 4 METHODS

### 4.1 Criteria for considering studies for this review

#### 4.1.1 Types of studies

This review is based on a published protocol (Naing 2019).

Included study designs were randomised controlled trials (RCTs), quasi‐experimental studies (QES), and other two‐group comparison designs (e.g., case‐control design, two cohorts). QES were inlcuded if baseline equivalence between intervention and control groups was established through matching, for example on: socioeconomic indices, school semester, enrolment, Cumulative Grade Point Average, and/or course taken.

We planned to inlcude, but did not find, cluster‐level randomised trials, natural experiments, and regression discontinuity designs.

We did not include qualitative research.

#### 4.1.2 Types of participants

We included studies conducted on undergraduate health professional students, regardless of the type of healthcare streams (e.g., medicine, dentistry, nursing, pharmacy), duration of the learning activity (e.g., one or two semesters) or the country where the study was conducted.

#### 4.1.3 Types of interventions

We included any educational intervention that included the flipped classroom as a teaching and learning tool in undergraduate programmes, regardless of the type of healthcare streams (e.g., medicine, nursing or pharmacy). We also included studies that explicitly indicated the teaching/learning activities for undergraduate students in the flipped classroom, reversed classroom, or flipping class, which aimed to improve student learning and/or student satisfaction (e.g., a study that compared a traditional lectured‐based class with a flipped class among undergraduate studies and measured academic performance and/or student satisfaction).

We excluded studies on standard lectures and subsequent tutorial formats (e.g., a study that compared a traditional lectured‐based class with a lectured‐based class and additional tutorials and measured exam scores and/or student satisfaction). Also, we excluded studies on flipped classroom methods among undergraduate or postgraduate students who are not from the healthcare streams (e.g., engineering, economics, or computer science).

#### 4.1.4 Types of outcome measures

We explored the impact of flipped classroom learning on undergraduate health professional students' academic‐related outcomes.

##### Primary outcomes

1. Academic performance was measured by examination scores, final grades or other formal assessment methods at immediate post‐test.
2. Student satisfaction (measured at immediate post‐test using a self‐report scale including the training institution's format of assessing student satisfaction).

Academic performance reflects indications of passing or failing based on cutoff point determined for the subject or course to evaluate the student's achievement against learning outcomes. The tests for passing/failing included the end‐of‐course assessment tests such as quiz items commonly used for both groups.

Student satisfaction is the measure of satisfaction with the course delivered based on the student's attitude towards the education experience, services, and facilities. It is not perceived quality as students can perceive a course as having a high degree of quality but remain unsatisfied with it.

We planned to assess the moderating effects (e.g., design, delivery, and the barriers and facilitators) of flipped classroom learning effectiveness for undergraduate health professional education. Due to limited data, we could only assess the moderating effect of study design on the effectiveness of flipped classroom interventions in undergraduate health professional education.

Outcomes were generally measured and then compared with the two methods of learning at the end of the interventions. However, in the pre‐post analysis, comparisons were done before and after implementation of the flipped class method. Substantial heterogeneity was observed due to variations in programme pathways (i.e., medicine, pharmacy, nursing, etc.), population characteristics, intervention context, outcome measures, and the tools used for outcome assessments across included studies.

For instance, even within the same programme pathway, the tools used in the Medicine programme ranged from the commonly used multiple‐choice questions (Grønlien, 2021; Hu, 2019), one‐best answer (OBA) (Isherwood, 2019), objective structured clinical examination (OSCE) (Anderson, 2017; Baris, 2020) to special tools such as Objective Structured Assessment of Technical Skills (OSATS) (Chiu, 2018). In the nursing pathway, more complex tools such as the self ‐efficacy evidence‐based practice (SE‐EBP) scale (Chu, 2019), and Ricketts' Critical Thinking Disposition Inventory (Dehghanzadeh, 2020) were used in the included studies. Please see more details in Supporting Information: Appendix 2.

##### Secondary outcomes

Following our research questions and objectives, we did not specify secondary outcome in this systematic review.

### 4.2 Search methods for identification of studies

#### 4.2.1 Electronic searches

A comprehensive search strategy was designed to identify the relevant studies in the following databases and search engines. The last search update occured April 29, 2022. The full details are presented in Supporting Information: Appendix 3.

(1) Electronic databases
  (a) MEDLINE (Ovid)
  (b) EMBASE (Ovid)
  (c) PubMed
  (d) Education Resources Information Centre (ERIC),
  (e) CENTRAL
  (f) SCOPUS
  (g) Best Evidence Medical Education
  (h) APA PsycInfo
  (i) Web of Science Core Collection
  (j) Google Scholar
(2) Research Registers and Websites
  (a) Cochrane Library
  (b) Campbell Library
  (c) Database of Abstracts of Reviews of Effectiveness
  (d) System for Information on Grey Literature
  (e) Evidence for Policy Practice Information and Coordinating Centre (EPPI‐Centre)
  (f) Applied Social Sciences Index and Abstracts (ASSIA)
(3) Dissertations and theses databases
  Proquest Global Dissertations and Theses
  Index to Theses in Great Britain and Ireland (www.theses.com)
  Theses Canada (www.collectionscanada.gc.ca/thesescanada/)
  Networked Digital Library of Theses and Dissertations (http://www.ndltd.org/)
(4) Regional bibliographic databases
  – Australia
    Australian Education Index (www.acer.edu.au/library/aei/index.html)
  – Britain
    British Education Index (www.leeds.ac.uk/bei/index.html)
  – Canada
    Canadian Business & Current Affairs (CBCA) Education (Proquest)
    Canadian Research Index (Proquest)
  – Latin America
    LILACS (http://lilacs.bvsalud.org/en/)
(5) Full‐text journals available electronically
  – BioMedCentral (www.biomedcentral.com/browse/journals/)
  – Public Library of Science (PLoS) (www.plos.org/journals/)
  – PubMedCentral (PMC) (www.pubmedcentral.nih.gov/)
  – Directory of Open Access Journals (DOAJ) (www.doaj.org)

##### Search terms

With the assistance of a Social Sciences and Evidence Synthesis Librarian (AR), we used several relevant search terms and subject headings combined with Boolean operators to target relevant studies. Such terms included ‘flipped classroom’, ‘inverted classroom,’ ‘health education’, and many more. In the final review, all searches originally performed were included so that they can be replicated. Proximity operators were used when appropriate, and search terms were truncated using the appropriate conventions for the given database or search engine syntax to include variations in the endings of words and spellings. Terms from different categories were connected with ‘OR’ within each category and by ‘AND’ between categories. The entire search strategy is provided in Supporting Information: Appendix 3.

#### 4.2.2 Searching other resources

To identify unpublished studies such as theses, conference proceeding, institutional reports, we searched grey literature sources by searching the following:

(a) Social Science Research Network
  We looked for studies from the year 2000 and onwards, regardless of the language or study setting.
(b) Conference abstracts and proceedings from the American Educational Research Association Repository (http://www.aera.net/EventsMeetings/tabid/10063/Default.aspx) for the year 2013−2017 were also reviewed to identify any potentially relevant studies.

To ensure that relevant studies were reviewed for inclusion in the meta‐analysis, we searched the following Institutional repositories;

- Canadian Institutional Repositories http://www.carl-abrc.ca/ir.html
- Directory of Open Access Repositories (OpenDOAR)
- Register of Open Access Repositories (ROAR)

We also searched existing reviews and publications to check references for studies that should be included (or excluded).

We also searched ongoing studies in the Social Care Online (http://www.scie-socialcareonline.org.uk).

We contacted the key researchers on the topic (Melissa Geist, Shinong Pan) about whether they had any studies in progress or unpublished research.

Lastly, we searched the Web using Google (www.google.com) and Bing (www.bing.com) to locate additional articles.

*Manual search*

Limited resources and personnel prevented us from conducting a comprehensive hand search of social science journals where flipped classroom‐based studies were previously published.

We conducted a hand search of journals that were relevant to the topic in

- *American Educational Research Journal* and
- *Journal of Educational Research*

We also identified relevant literature from the reference lists of the potentially eligible studies retrieved for full‐text screening and we included such studies in the full‐text screening.

We did a double screen by two investigators and inter‐rater agreement was assessed using Cohen's *κ*.

### 4.3 Data collection and analysis

#### 4.3.1 Selection of studies

Two review authors (CN, DKC) independently extracted data from included studies. A coding sheet was piloted based on several studies and was then revised. Any disagreements were resolved by discussion and a consensus was reached in all cases.

The two investigators independently screened 40% of the records, where the Cohen's *κ* 0.83 indicated strong agreement.

#### 4.3.2 Data extraction and management

Two review authors (CN, DKC) independently extracted data from the included studies. Any disagreements were resolved by discussion and a consensus was reached in all cases.

We extracted the following data from each study included in this review.

Description of study: type of study design, study country, study setting (e.g., college/university/institute, discipline).

Description of participants: type of study participants (e.g., gender, age group, year at school).

Description of the educational programme: for example, duration of the flipped class, comparators, modality of intervention such as video lecture, YouTube lecture, and so forth.

Description of the comparator/any other interventions in addition to the education method.

Main outcomes: primary and secondary outcomes, outcome measurements (e.g., definition of the outcome, tools used to measure the outcome, time points of outcome measurement), and any additional information that potentially affected the results.

We corresponded with investigators of the primary studies (i.e., Geist, 2015) to clarify study eligibility or any missing information (e.g., baseline equivalence). When an author query did not retrieve the requested data, the study was still reported but was not included in the final meta‐analysis. Extracted data was stored in a Microsoft Excel sheet.

#### 4.3.3 Assessment of risk of bias in included studies

We assessed the risk of bias at the study level by using the Cochrane Risk of Bias tool (Higgins, 2011a). For non‐randomised designs, we used the ‘Risk of Bias’ tool from the Cochrane Effective Practice and Organisation of Care Group (EPOC, 2009) with some modifications. The tool used covers allocation sequence, the similarity of baseline outcome measurement, the similarity of baseline characteristics, incomplete outcome data, blinding of allocation, protection against contamination, selective outcome reporting, and other risks of bias. We prepared a risk of bias table that includes both RCTs (and/or non‐RCTs and/or controlled before‐after (CBA) studies) and interrupted time series (ITS) studies in Review Manager 5.4.1 (RevMan Web, 2019), as suggested in Risk of Bias Criteria for EPOC reviews (EPOC, 2017b). The two review authors (HHA, CN) independently assessed the risk of bias. For most of the items, we rated them as ‘yes’ (low risk of bias), ‘no’ (high risk of bias), or ‘unclear’ (unclear risk of bias) to make judgements of risk of bias.

Two investigators independently evaluated all eligible studies, where the Cohen's *κ* 0.86 indicated perfect agreement. Discrepancies were settled by consensus and consulted a third investigator of the team (DKC) if needed.

We presented an overall grading of the evidence related to each of the main outcomes using the GRADE (Grades of Recommendation, Assessment, Development, and Evaluation) approach. The GRADE approach defines the quality of a body of evidence as to the extent to which one can be confident that an estimate of effect or association is close to the true quantity of specific interest. The quality of a body of evidence involves the consideration of the risk of bias within a trial (methodological quality), the directness of evidence, heterogeneity, the precision of effect estimates, and the risk of publication bias (Schünemann, 2011). A level of evidence for the ‘body of evidence’ is assigned, ranging from high, moderate, low to very low, as part of the GRADE process (Atkins, 2004). We did not exclude studies on the grounds of risk of bias, but sources of bias were reported when presenting the results of studies. We presented all included studies and provided a narrative discussion on the risk of bias together with the potential limitations of the review as well as implications of bias in the interpretation of the results under the ‘Discussion’ section of the full‐text review.

#### 4.3.4 Measures of treatment effect

##### Methods for handling dependent effect sizes

If the independence assumption was violated by studies reporting several estimates based on the same individuals or if there were clusters of studies that were not independent (such as those carried out by the same facilitator), then we planned to use the robust variance estimator of the covariance matrix of meta‐regression coefficients, as described elsewhere (Hedges, 2010; Higgins, 2020). We did not find any study that required us to use a robust variance estimator in this review.

#### 4.3.5 Unit of analysis issues

In cluster‐randomised trials, the unit of allocation is a group, rather than an individual. In such an event, we used cluster‐level assignment planned to adjust the standard errors of all effect size estimates using the Methods of analysis for cluster‐randomised trials (23.1.3) of the Cochrane Handbook (Higgins, 2020). If the intra‐class correlation that was needed to make this adjustment was not reported in the primary studies, we planned to use similar intraclass correlations reported in other education trials (Hedges, 2007) and planned to conduct sensitivity analyses using a range of plausible values.

If the included cluster‐randomised trials sufficiently account for the cluster design, we planned to include the effect estimates in the meta‐analysis. However, there were no cluster‐randomised trials identified for this review.

#### 4.3.6 Dealing with missing data

We contacted the respective corresponding author for any missing standard deviations (SDs) for continuous outcomes or study characteristics (i.e., Geist, 2015; Lin, 2017; Wu, 2020). If these were not available, we calculated these using case‐analysis such as imputing SDs from standard errors (SEs), CIs, *t‐*values or *p* values (as appropriate) that were related to the differences between means in two groups, as described in the *Cochrane Handbook for Systematic Reviews of Interventions* (Higgins, 2020).

When there was insufficient information available to calculate the SDs, we imputed SDs. We imputed the SD of the mean difference of each group, using the calculator provided in RevMan (RevMan Web, 2019). The effect of missing data on the overall results was assessed through sensitivity analysis by doing a meta‐analysis without imputing missing information.

#### 4.3.7 Assessment of heterogeneity

We assessed statistical heterogeneity using the *χ* ^2^ test, *τ* ^2^ test, and the *I* ^*2*^ measure. The *χ* ^2^ test assesses whether the observed differences in results are compatible with chance alone. The *τ* ^2^ test is an estimate of the between‐study variance in a random‐effects meta‐analysis (Deeks, 2020). The *I* ^2^ measure examines the percentage of total variation across studies due to (statistical) heterogeneity rather than to chance and we interpreted *I* ^2^ values as in Deeks (2020):

- 0%–40%: might not be important;
- 30%–60%: may represent moderate heterogeneity;
- 50%–90%: may represent substantial heterogeneity;
- 75%–100%: considerable heterogeneity.

#### 4.3.8 Assessment of reporting biases

Based on a required number of studies, we used funnel plots to display the information about possible publication bias only on examination score in the medical programme. We were not able to assess publication bias on other outcomes or in other programmes identified for this review.

#### 4.3.9 Data synthesis

The primary goal of this meta‐analysis was to address primary and secondary research questions by estimating the effect of flipped class on student academic outcomes and students’ satisfaction outcomes, and by examining the extent to which these outcomes are moderated by study characteristics, including fidelity of implementation.

When there were at least two studies with the same comparison (flipped classroom group vs traditional lecture class group) on the same outcome, we employed meta‐analysis. More studies were needed for a moderator analysis (Borenstein, Hedges, Higgins, & Rothstein, 2009).

For dichotomous outcomes, we used risk ratio (RR) and respective 95% confidence interval (CI) and we conducted meta‐analyses, based on RRs and summarised the results as a summary RR and its 95% CI.

For continuous outcomes such as mean and SD, we used standardised mean difference (SMD) and its 95% CIs as studies used different scales of measurement. We interpreted SMD as follows (Schünemann, 2022).

- SMD less than 0.40 represents a small intervention effect.
- SMD between 0.40 and 0.70 represents a moderate intervention effect.
- SMD greater than 0.70 represents a large intervention effect.

For studies with continuous data as median and range values or median and interquartile, we planned to calculate the means and standard deviations using statistical algorithms as described elsewhere (Luo, 2018; Wan, 2014).

An SMD greater than zero or RR greater than 1 indicates an increase in the outcome in the intervention group (flipped classroom) compared to the comparison group.

In performing the meta‐analysis, we synthesised the effect sizes for each outcome using the inverse‐variance random‐effects meta‐analysis.

We used RevMan (RevMan Web, 2019) to conduct the meta‐analysis. We did not combine evidence from different designs and outcome types in the same Forest Plot.

Results were reported using Forest Plots with study sample sizes, effect sizes, 95% CIs, *p*‐values, tests of homogeneity, and model choice of random effects.

#### 4.3.10 Subgroup analysis and investigation of heterogeneity

Based on a sufficient number of studies reporting the relevant data, we stratified analysis including:

- Study design: Do randomised and non‐randomised designs exhibit consistently different effect sizes and significance values?

We planned a moderator analysis with sub‐specialty (e.g., ophthalmology, pharmacology, epidemiology), amount of out‐of‐class preparation time, classroom availability and limited high‐speed Internet access for rural and remote students, quality of interactive tools used, and/or faculty members' preference for a more didactic approach. However, only limited studies included in the main meta‐analysis also reported this data.

#### 4.3.11 Sensitivity analysis

Based on the required number of studies, we performed the sensitivity analysis on studies that used imputed data values to explore its impact on the effect estimates. This was necessarily performed for one main outcome namely the academic performance (final grade/exam scores), which is described under Section 10. We imputed data as described in the section ‘Dealing with missing data’.

We also planned to perform sensitivity analysis by removing studies with an overall high and unclear risk of bias from the meta‐analyses. Therefore, the analysis would include only studies with an overall low risk of bias in all key domains. However, almost all studies included had a high risk of bias. Hence, we did not perform sensitivity analysis for the risk of bias.

We planned to perform analysis using different plausible values for intraclass correlation estimation especially for studies with cluster assignment. However, there were insufficient studies in the meta‐analysis to conduct this sensitivity analysis.

##### Summary of findings and assessment of the certainty of the evidence

We presented an overall assessment of the certainty of the evidence related to each of the main outcomes using the GRADE (Grades of Recommendation, Assessment, Development and Evaluation) approach. The GRADE approach defines the quality of a body of evidence as to the extent to which one can be confident that an estimate of effect or association is close to the true quantity of specific interest. The quality of a body of evidence involves the consideration of the risk of bias within the trial (methodological quality), directness of evidence, heterogeneity, the precision of effect estimates, and the risk of publication bias (Schünemann, 2011). A level of evidence for the ‘body of evidence’ is assigned, ranging from high, moderate, low to very low, as part of the GRADE process (Atkins, 2004). We do not exclude studies on the grounds of risk of bias, but sources of bias are reported when presenting the results of studies. We presented all included studies and provided a narrative discussion on the risk of bias together with the potential limitations of the review as well as implications of bias in the interpretation of the results under the ‘Discussion’ section of the full‐text review.

## 5 RESULTS

### 5.1 Description of studies

The summary characteristics of included and excluded studies are presented in Characteristics of excluded studies; Characteristics of studies awaiting classification; Characteristics of ongoing studies.

#### 5.1.1 Results of the search

**Figure 1:**
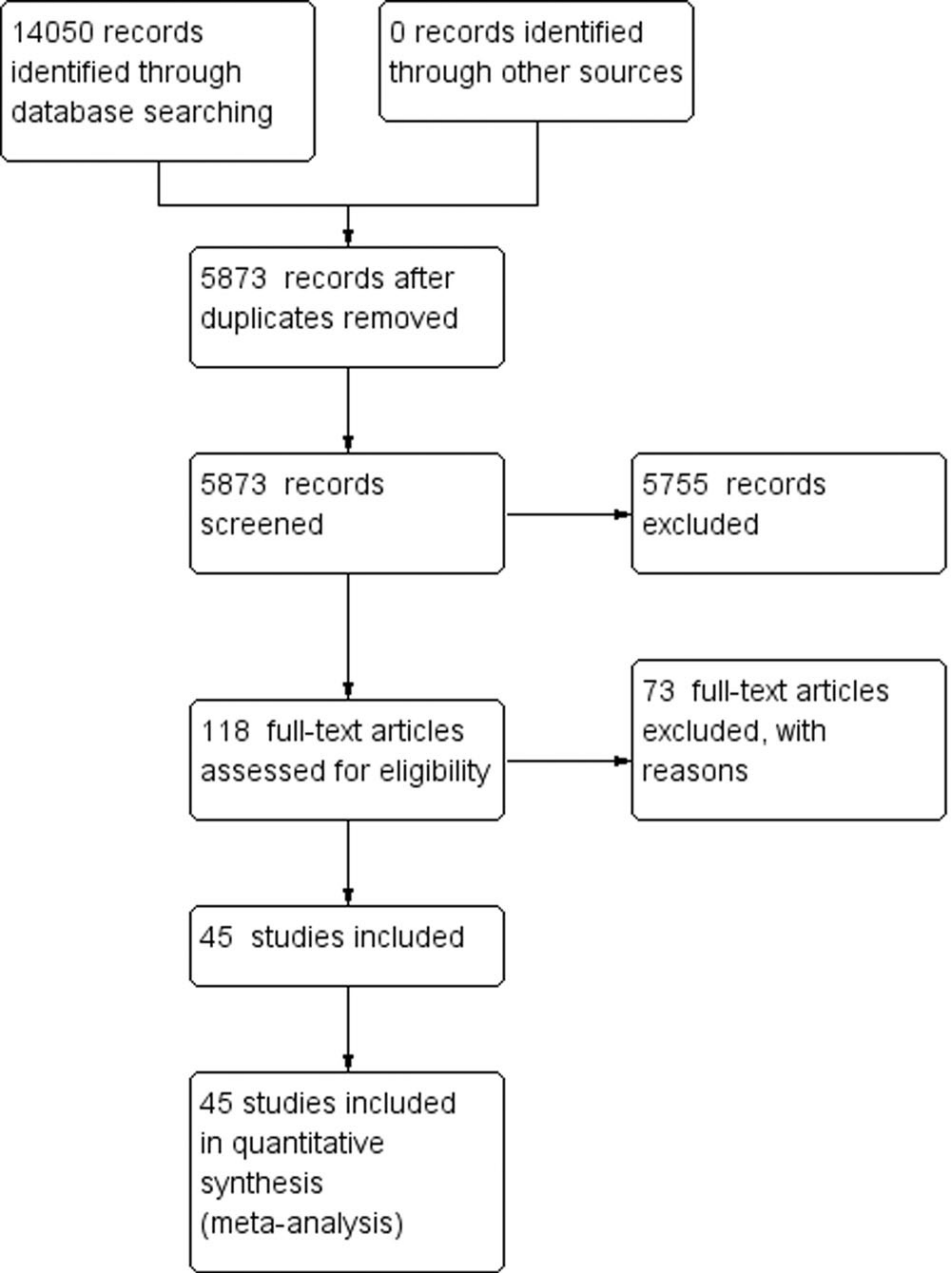
Study selection process.

Studies retrieved from literature searches were screened. Figure 1 summarises the study selection process.

A comprehensive search identified 14,050 items from the reproducible search strategies listed under the section of identification of studies and search strategy under the Methods section and presented in Supporting Information: Appendix 3. Duplicates (*n* = 8177) were removed using the duplication detection features of EndNote and Covidence. An initial search was conducted in November 2019 followed by a search update in April 2022. We filtered all records based on title and abstract. We removed 5755 records based on titles and abstracts. We screened the remaining 118 records in full text. In total, 45 studies met the inclusion criteria for this review, and we extracted data from these studies. Two investigators independently screened the records, where the Cohen's *κ* 0.83 indicated strong agreement.

#### 5.1.2 Included studies

We included all 45 studies with a total of 8426 participants in the meta‐analysis. Details of individual studies are presented in the Characteristics of included studies.

##### Study design

Eleven of the 45 included studies were RCTs (Anderson, 2017; Chiu, 2018; Dodiya, 2019; Harrington, 2015; Heitz, 2015; Isherwood, 2019; Kuhl, 2017; Ren, 2020; Rui, 2017; Wang, 2021; Zheng, 2020), and 19 were QES (Angadi, 2019; Baris, 2020; Chu, 2019; Dehghanzadeh, 2020; Fan, 2020; Grønlien, 2021; Herrero, 2020; Hu, 2019; Huang, 2020; Lin, 2017; Lucchetti, 2018; Missildine, 2013; Park, 2018; Sajid, 2020; Sinclair‐Bennett, 2019; Street, 2015; Suda, 2014; Tang, 2017; Zhu, 2020), and 15 were observational studies of two group comparison designs (i.e., case‐control design or two different cohorts) (Bossaer, 2016; Boysen‐Osborn, 2016; Burak, 2015; Chaudhuri, 2019; Cheng, 2016; Cotta, 2016; Evans, 2016; Gillispie, 2016; Morton, 2017; O'Connor, 2016; Stewart, 2013; Whelan, 2015; Whillier, 2015; Wilson, 2016; Wong, 2014). We could not identify any eligible ITS or cluster‐RCT for this review.

##### Participants in various disciplines

Participants of 24 studies were undergraduate medical students in various academic/school years or semesters in a variety of disciplines/modules (Angadi, 2019; Baris, 2020; Boysen‐Osborn, 2016; Burak, 2015; Chaudhuri, 2019; Chiu, 2018; Dodiya, 2019; Evans, 2016; Gillispie, 2016; Heitz, 2015; Herrero, 2020; Hu, 2019; Kuhl, 2017; Lin, 2017; Lucchetti, 2018; Morton, 2017; O'Connor, 2016; Ren, 2020; Rui, 2017; Sajid, 2020; Street, 2015; Tang, 2017; Whelan, 2015; Zheng, 2020). Seven studies included undergraduate pharmacy students (Anderson, 2017; Bossaer, 2016; Cotta, 2016; Stewart, 2013; Suda, 2014; Wilson, 2016; Wong, 2014), while eight studies were with undergraduate nursing students (Chu, 2019; Dehghanzadeh, 2020; Fan, 2020; Grønlien, 2021; Harrington, 2015; Missildine, 2013; Park, 2018; Sinclair‐Bennett, 2019), one study included undergraduates from the medical, dental, and nursing schools (Zhu, 2020), and the remaining five studies involved other disciplines such as dentistry (Isherwood, 2019; Wang, 2021) and allied health sciences such as chiropractic (Whillier, 2015), Chinese medicine (Cheng, 2016) and medical technology (Huang, 2020).

##### Location of studies

Studies were frequently (35.6%, 16/45) carried out in the high‐income countries such as the USA (Anderson, 2017; Bossaer, 2016; Boysen‐Osborn, 2016; Cotta, 2016; Evans, 2016; Harrington, 2015; Heitz, 2015; Missildine, 2013; Morton, 2017; O'Connor, 2016; Sinclair‐Bennett, 2019; Stewart, 2013; Street, 2015; Suda, 2014; Wilson, 2016; Wong, 2014), followed by eight studies (17.8%) in China (Cheng, 2016; Hu, 2019; Lin, 2017; Ren, 2020; Rui, 2017; Tang, 2017; Zheng, 2020; Zhu, 2020).

##### Interventions

All these studies used flipped class teaching/blended class as an intervention, albeit with variation in their implementation. For instance, a study used flipped class in the 2012 cohort, while using a traditional class in the 2011 cohort (Wong, 2014). Another study used flipped class in 2010 and traditional class in 2009 (Stewart, 2013). Also, a study used flipped class in the 2013‐2014 cohort and traditional class in the 2012–2013 cohort (Street, 2015). The contents covered by interventions varied within the discipline. For example in Medicine, one study used flipped class in radiology module (O'Connor, 2016), two studies were done on ophthalmology course (Lin, 2017; Tang, 2017), while one study each was done in advanced cardiac life support (Boysen‐Osborn, 2016), epidemiology (Evans, 2016), hepatology (Burak, 2015) or laparoscopic skill training modules (Chiu, 2018). In the context of the Pharmacy discipline, two single studies were carried out on cardiac arrhythmias (Wong, 2014) and oncology modules (Bossaer, 2016).

##### Comparisons

In most of the studies (97.8%, 44/45) classes used conventional/traditional lecture‐based class/large classroom‐based lecture as a comparator, while the remaining studies compared the flipped class with historical cohort (i.e., used their historical performance data) of traditional class (Evans, 2016).

##### Outcomes

Forty‐four studies (97.8%,44/45) reported on examination scores/grades (Anderson, 2017; Angadi, 2019; Baris, 2020; Bossaer, 2016; Boysen‐Osborn, 2016; Burak, 2015; Chaudhuri, 2019; Cheng, 2016; Chiu, 2018; Chu, 2019; Cotta, 2016; Dehghanzadeh, 2020; Dodiya, 2019; Fan, 2020; Gillispie, 2016; Grønlien, 2021; Harrington, 2015; Heitz, 2015; Herrero, 2020; Hu, 2019; Huang, 2020; Isherwood, 2019; Kuhl, 2017; Lin, 2017; Lucchetti, 2018; Missildine, 2013; Morton, 2017; O'Connor, 2016; Park, 2018; Ren, 2020; Rui, 2017; Sajid, 2020; Sinclair‐Bennett, 2019; Stewart, 2013; Street, 2015; Suda, 2014; Tang, 2017; Wang, 2021; Whelan, 2015; Whillier, 2015; Wilson, 2016; Wong, 2014; Zheng, 2020; Zhu, 2020). Eight studies reported on student satisfaction; six studies (13.3%) assessed student satisfaction in continuous data (Evans, 2016; Fan, 2020; Missildine, 2013; Sinclair‐Bennett, 2019; Street, 2015; Whelan, 2015), while two studies (4.4%) assessed in dichotomous options (Herrero, 2020; Tang, 2017).

These studies measured the outcomes with various tools. For instance, students' performance in examinations was most frequently assessed with multiple‐choice questions (MCQs) (Angadi, 2019; Bossaer, 2016; Boysen‐Osborn, 2016; Chaudhuri, 2019; Cheng, 2016; Gillispie, 2016; Heitz, 2015; Herrero, 2020; Stewart, 2013; Suda, 2014; Tang, 2017; Wong, 2014). Some studies used content‐specific assessment tools such as the Ricketts' Critical Thinking Disposition Inventory (Dehghanzadeh, 2020), and the Forensic test score (Huang, 2020).

#### 5.1.3 Excluded studies

Details of individual studies are presented in the Characteristics of excluded studies.

Of the 118 full‐text reviewed, we excluded 73 studies. Due to the large number of studies screened in full text, we were unable to describe each excluded study in detail. We excluded studies as they did not target the health professional undergraduates. For example, two studies (Koo, 2016; Martinelli, 2017) were focused solely on postgraduate programs. We also excluded studies that did not include two separate groups for comparison (Armbruster, 2009; Belfi, 2015; Busebaia, 2020; Libert, 2016; Sheppard, 2017; Sohn, 2019; Vadakedath, 2019; Vavasseur, 2020; Veeramani, 2015; Wu, 2020).

### 5.2 Risk of bias in included studies

This review included a total of 45 studies: 11 RCTs, 19 QES, and 15 observational studies).

**Figure 2:**
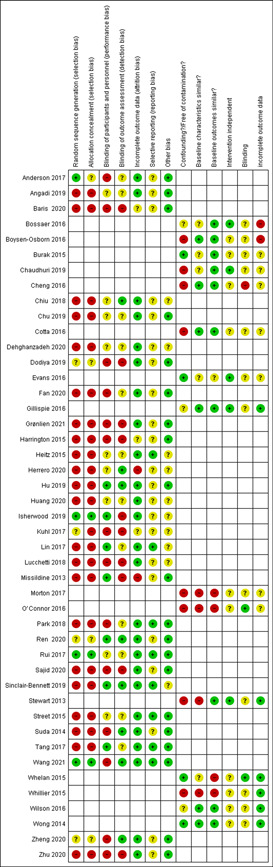
Risk of bias summary: Review authors' judgements about each risk of bias item for each included study.

To assess the risk of bias, we used the Cochrane Risk of Bias tool (Higgins, 2011a) and expanded domains for non‐randomised designs, as described in the Cochrane Effective Practice and Organisation of Care Group (EPOC, 2009) with some modifications (Figure 2).

#### 5.2.1 Allocation (selection bias)

In 11 RCTs, four studies were adequately done on random sequence generation (Anderson, 2017; Isherwood, 2019; Rui, 2017; Wang, 2021) and were judged as having a low risk of selection bias. Three RCTs (Chiu, 2018; Harrington, 2015; Heitz, 2015) were judged as having a high risk of selection bias and four RCTs (Dodiya, 2019; Kuhl, 2017; Ren, 2020; Zheng, 2020) were judged as having an ‘unclear risk of bias’ due to inadequate randomisations.

Allocation concealment was adequately reported in only three RCTs (Isherwood, 2019; Rui, 2017; Wang, 2021) and was judged as having a low risk of selection bias. Four RCTs (Chiu, 2018; Harrington, 2015; Heitz, 2015; Kuhl, 2017) were judged as having a high risk of selection bias and another four RCTs (Anderson, 2017; Dodiya, 2019; Ren, 2020; Zheng, 2020) was having an unclear risk of allocation concealment.

Randomisation was not used in 19 QES studies, and therefore, was judged as having a high risk of selection bias. These 19 QES studies did not adequately report, or there was a lack of information on allocation concealment and were judged as having a high risk of selection bias. Of note, QES has a risk of bias by default on selection bias since these two items (random sequences generation and allocation concealment) were not usually performed in this type of study.

#### 5.2.2 Blinding (performance bias and detection bias)

##### Performance bias

Two RCTs (Isherwood, 2019; Ren, 2020) were judged as having a low risk of performance bias. It was stated that ‘unseen by the participants’ (Isherwood, 2019), and ‘all students were unaware of their group assignments before class’ (Ren, 2020). Six RCTs (Anderson, 2017; Dodiya, 2019; Harrington, 2015; Kuhl, 2017; Wang, 2021; Zheng, 2020) were judged as having a high risk of bias due to a lack of blinding the students about their assigned method of teaching. For instance, the same instructors (study investigators) were assigned to teach both course sections (Anderson, 2017). Hence, they would be able to identify the participants from each group at the time of evaluation. An open‐label design (Dodiya, 2019), and the assessors were able to distinguish which group the participants belonged to as the experimental group received the question paper as a hard copy on‐site, and the ‘control’ (traditional group) has the same question paper delivered and replied via email (Kuhl, 2017). Hence, they would be able to identify the participants from each group at the time of evaluation. The remaining three RCTs (Chiu, 2018; Heitz, 2015; Rui, 2017) were judged as having an unclear risk of bias due to insufficient information on blinding.

Six QES (Hu, 2019; Lin, 2017; Missildine, 2013; Park, 2018 Sinclair‐Bennett, 2019; Tang, 2017) were judged as having a low risk of performance bias. Six QES (Baris, 2020; Fan, 2020; Grønlien, 2021; Lucchetti, 2018; Park, 2018; Suda, 2014) were judged as having a high risk of bias due to the lack of blinding among the students about their assigned method of teaching. The remaining seven QES (Angadi, 2019; Chu, 2019; Dehghanzadeh, 2020; Grønlien, 2021; Herrero, 2020; Huang, 2020; Street, 2015) were judged as having an unclear risk of bias due to insufficient information on blinding.

*Detection bias*

Four RCTs (Chiu, 2018; Ren, 2020; Wang, 2021; Zheng, 2020) adequately blinded the outcome assessors and were judged as having a low risk of detection bias. We judged four RCTs (Dodiya, 2019; Harrington, 2015; Isherwood, 2019; Kuhl, 2017) as having a high risk of detection bias since the outcome assessors were not adequately blinded. We judged three RCTs (Anderson, 2017; Heitz, 2015; Rui, 2017) as having an unclear risk of detection bias due to inadequately reported blinding of the assessors.

We judged four QES (Herrero, 2020; Hu, 2019; Sinclair‐Bennett, 2019; Suda, 2014) as having a low risk of detection bias, while six QES (Baris, 2020; Grønlien, 2021; Lucchetti, 2018; Missildine, 2013; Sajid, 2020; Zhu, 2020) as having a high risk of detection bias, since the outcome assessors were not adequately blinded. The remaining nine QES (Angadi, 2019; Chu, 2019; Dehghanzadeh, 2020; Fan, 2020; Huang, 2020; Lin, 2017; Park, 2018; Street, 2015; Tang, 2017) were judged as having an unclear risk of detection bias.

#### 5.2.3 Incomplete outcome data (attrition bias)

We judged nine RCTs (Anderson, 2017; Chiu, 2018; Dodiya, 2019; Heitz, 2015; Isherwood 2019; Ren, 2020; Rui, 2017; Wang, 2021; Zheng, 2020) as having a low risk of attrition bias since there was no significant loss to follow‐up, while two RCTs (Harrington, 2015; Kuhl, 2017) were judged as having an unclear risk of attrition bias due to inadequate information.

We judged 16 QES (Angadi, 2019; Chu, 2019; Dehghanzadeh, 2020; Fan, 2020; Grønlien, 2021; Hu, 2019; Huang, 2020; Lin, 2017; Lucchetti, 2018; Park, 2018; Sajid, 2020; Sinclair‐Bennett, 2019; Street, 2015; Suda, 2014; Tang, 2017; Zhu, 2020) as having a low risk of attrition bias. Two QES (Herrero, 2020; Missildine, 2013) were judged as having a high risk of attrition bias. The remaining study (Baris, 2020) was judged as having an unclear risk of attrition bias due to inadequate information.

#### 5.2.4 Selective reporting (reporting bias)

We judged three RCTs (Heitz, 2015; Rui, 2017; Wang, 2021) as having a low risk of bias since these studies reported baseline information for one of the outcomes/according to the protocols. Eight RCTs (Anderson, 2017; Chiu, 2018; Dodiya, 2019; Harrington, 2015; Isherwood, 2019; Ren, 2020; Sajid, 2020) were judged as having an unclear risk of reporting bias since we could not access their protocols.

We judged five QES (Lin, 2017; Park, 2018; Sinclair‐Bennett, 2019; Street, 2015; Tang, 2017) as having a low risk of bias. Fourteen QES (Angadi, 2019; Baris, 2020; Chu, 2019; Dehghanzadeh, 2020; Fan, 2020; Grønlien, 2021; Herrero, 2020; Hu, 2019; Huang, 2020; Lucchetti, 2018; Missildine, 2013; Park, 2018; Sajid, 2020; Suda, 2014; Zhu, 2020) were judged as having unclear risk.

Two investigators independently screened the records, and Cohen's κappa 0.83 indicated strong agreement.

#### 5.2.5 Other potential sources of bias

We judged three studies (Anderson, 2017; Harrington, 2015; Rui, 2017) as having a low risk of bias and the remaining eight RCTs (Chiu, 2018; Dodiya, 2019; Heitz, 2015; Isherwood, 2019; Kuhl, 2017; Ren, 2020; Wang, 2021; Zheng, 2020) as unclear risk of other potential sources of bias.

We judged 13 QES (Angadi, 2019; Baris, 2020; Chu, 2019; Fan, 2020; Grønlien, 2021; Hu, 2019; Missildine, 2013; Park, 2018; Sajid, 2020; Street, 2015; Suda, 2014; Tang, 2017; Zhu, 2020) as having a low risk of bias and the remaining six QES (Dehghanzadeh, 2020; Herrero, 2020; Huang, 2020; Lin, 2017; Lucchetti, 2018; Sinclair‐Bennett, 2019) as unclear risk of other potential sources of bias. Concerns over ‘conflict of interest is an important factor regarded as another source of bias in this review.

*The additional risk of bias in observational two groups design (i.e., case‐control or two cohorts)*

**i. Confounding**

Of 15 observational studies, four studies (Burak, 2015; Evans, 2016; Whelan, 2015; Wong, 2014) had a low risk of confounding bias since the flipped class and comparator classes were implemented with adequate time intervals. We judged eight studies (Boysen‐Osborn, 2016; Chaudhuri, 2019; Cheng, 2016; Cotta, 2016; Morton, 2017; O'Connor, 2016; Stewart, 2013; Whelan, 2015) as having a high risk of bias because the students in those two groups were from the same school and may have been aware of which participants were assigned to which group and/or which examination questions were used, or because they were volunteer participants, where no other details of their underlying characteristics such as age and education level. Hence, there was a risk of contamination between groups. The remaining three studies (Bossaer, 2016; Gillispie, 2016; Wilson, 2016) were judged to have an unclear risk of confounding bias.

**ii. Baseline characteristic imbalance**

In six observational studies (Boysen‐Osborn, 2016; Cheng, 2016; Cotta, 2016; Gillispie, 2016; Wilson, 2016; Wong, 2014), baseline characteristics were similar and had a low risk of bias. We judged four studies (Morton, 2017; O'Connor, 2016; Stewart, 2013; Whillier, 2015) as having a high risk of bias due to an imbalance in the number of participants or an imbalance in the proportion of males in the two groups.

The remaining five studies (Bossaer, 2016; Burak, 2015; Chaudhuri, 2019; Evans, 2016; Whelan, 2015) were rated as having an unclear risk of baseline imbalance due to a lack of information.

**iii. Baseline outcomes similar**

We judged six studies (Boysen‐Osborn, 2016; Cheng, 2016; Cotta, 2016; Gillispie, 2016; Wilson, 2016; Wong, 2014) as having a low risk of bias due to the use of the same tests in both, while four studies (Morton, 2017; O'Connor, 2016; Stewart, 2013; Whillier, 2015) as high risk since the studies used different exam items and five studies (Bossaer, 2016; Burak, 2015; Chaudhuri, 2019; Evans, 2016; Whelan, 2015) as unclear risk of outcome imbalance.

**iv. Intervention independent**

We judged five studies (Bossaer, 2016; Chaudhuri, 2019; Evans, 2016; Gillispie, 2016; Stewart, 2013) as having a low risk of intervention dependence bias since the flipped class and traditional lecture‐based class were not implemented in the same cohort at the same time, while 10 studies (Boysen‐Osborn, 2016; Burak, 2015; Cheng, 2016; Cotta, 2016; Morton, 2017; O'Connor, 2016; Whelan, 2015; Whillier, 2015; Wilson, 2016; Wong, 2014) as unclear risk of independent intervention.

**v. Analysed appropriately**

Twelve observational studies (Bossaer, 2016; Boysen‐Osborn, 2016; Burak, 2015; Evans 2016; Gillispie, 2016; Morton, 2017; O'Connor, 2016; Stewart, 2013; Whelan, 2015; Whillier, 2015; Wilson, 2016; Wong, 2014) had a low risk of bias since these studies were analysed appropriately. We judged three studies (Chaudhuri, 2019; Cheng, 2016; Cotta, 2016) as having an unclear risk of bias.

**vi. Blinding**

We judged two studies (O'Connor, 2016; Whillier, 2015) as having a low risk of bias based on adequate information on blinding. One study (Cheng, 2016) was judged as having a high risk of bias and the remaining 12 studies (Bossaer, 2016; Boysen‐Osborn, 2016; Burak, 2015; Chaudhuri, 2019; Cotta, 2016; Evans, 2016; Gillispie, 2016; Morton, 2017; Stewart, 2013; Whillier, 2015; Wilson, 2016; Wong, 2014) were judged as having an unclear risk of bias.

**vii. Addressing incomplete outcome data**

We judged four studies (Gillispie, 2016; Morton, 2017; Stewart, 2013; Whillier, 2015) as having a low risk of bias due to a low non‐response rate. Four studies (Boysen‐Osborn, 2016; Whelan, 2015; Wilson, 2016; Wong, 2014) were judged as having a high risk of bias and the remaining seven studies (Bossaer, 2016; Burak, 2015; Chaudhuri, 2019; Cheng, 2016; Cotta, 2016; Evans, 2016; O'Connor, 2016) as having unclear risk.

In brief, the studies included had problems with randomisation, allocation concealment, and confounding, and this will be returned to the sensitivity testing of our results in Section 10.

### 5.3 Effects of interventions

Overall, 45 studies were included across all the various analyses that are described subsequently. We extracted data from the included studies, and then, the effect estimates were calculated. The most frequently reported effect estimates were the examination scores/grades in 44 studies (44/45, 97.8%).

#### 5.3.1 Primary outcomes

##### Academic performance (measured with final examination score/grade) (Analysis 1.1; Figure 3).

Forty‐four studies (*n* = 7813) reported academic performance measured with final examination grades/scores. Academic performance was higher in the flipped class group compared to the traditional class group (SMD 0.57, 95% CI 0.25, 0.90, 44 studies, *n* = 7813). Heterogeneity was substantial (*τ* ^2^ = 1.16, *p* < 0.00001; *I* ^2^: 98%). The SMD of 0.57 can be interpreted as a moderate effect size.

Although a large effect size was observed in five studies included (i.e., Burak, 2015; Gillispie, 2016; Stewart, 2013; Whelan, 2015; Wong, 2014), concerns still remain about whether the flipped teaching curriculum is truly effective for more complex and time‐consuming topics (Wong, 2014).

It is possible that if the study had evaluated all exam questions, results would likely be affected by a ‘watering down’ effect as some questions pertain to other learning outcomes. If this is the case, then the analysis used is more appropriate to the teaching technique used than to any end‐of‐course exam scores not limited to specific learning outcomes (Stewart, 2013).

##### Students' satisfaction with the method of learning

Eight studies measured student satisfaction (Analysis 2.1; Figure 4).

Eight studies (*n* = 1696) reported students' satisfaction with the method of learning for the two groups. Students' satisfaction was higher in the flipped class group (SMD: 0.48, 95% CI: 0.15, 0.82, 8 studies, *n* = 1696). Heterogeneity was substantial (*τ* ^2^ = 0.19, *p* < 0.00001; *I* ^2^: 89%). The SMD of 0.48 can be interpreted as a moderate effect size.

##### Moderator effects

We performed a moderator analysis to investigate the influences of study design (please see 2.1 Academic Performance by study design). Due to the paucity of data, we could not assess other moderator effects such as school setting, semester, course contents, previous achievement, and delivery time.

One study included in this review reported that students' academic achievement was found to be significantly associated with the level of student's previous achievement of the cumulated GPA (*p* < 0.05) (Park, 2018). This was also reported in another study (*p* < 0.001) (Anderson, 2017).

##### Academic Performance by study design

###### RCT (Analysis 3.1; Figure 5)

Eleven studies using RCTs (Anderson, 2017; Chiu, 2018; Dodiya, 2019; Harrington, 2015; Heitz, 2015; Isherwood, 2019; Kuhl, 2017; Ren, 2020; Rui, 2017; Wang, 2021; Zheng, 2020) reported better academic performance in the flipped class group compared to the traditional class group (SMD: 0.42, 95% CI: 0.18, 0.65, 11 studies, *n* = 1398). Heterogeneity was high (*τ* ^2^ = 0.12, *p* = 0.0001, *I* ^2^: 75%). The SMD of 0.42 can be interpreted as a moderate effect size.

###### QES (Analysis 3.2)

Nineteen QES (Angadi, 2019; Baris, 2020; Chu, 2019; Dehghanzadeh, 2020; Fan, 2020; Grønlien, 2021; Herrero, 2020; Hu, 2019; Huang, 2020; Lin, 2017; Lucchetti, 2018; Missildine, 2013; Park, 2018; Sajid, 2020; Sinclair‐Bennett, 2019; Street, 2015; Suda, 2014; Tang, 2017; Zhu, 2020) reported better examination scores in the flipped class group compared to the traditional class group (SMD: 0.52, 95% CI: 0.21, 0.83, 19 studies, *n* = 3894). There was substantial heterogeneity (*τ* ^2^ = 0.43, *p* < 0.00001, *I* ^2^: 95%). The SMD of 0.52 can be interpreted as a moderate effect size.

**Figure 3:**
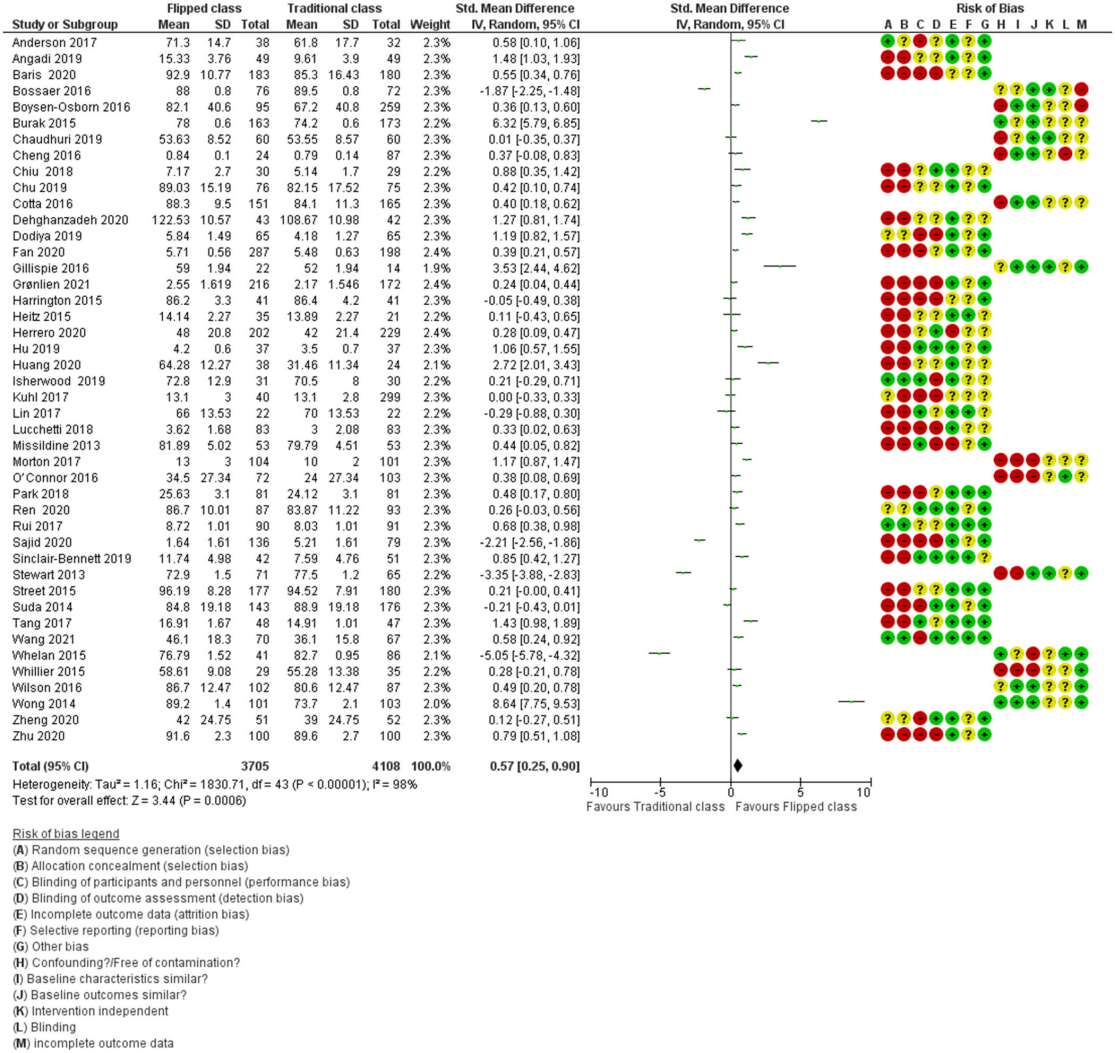
(Analysis 1.1) Forest plot of comparison: 1 Flipped versus Traditional, outcome: 1.2 Final Grade/t immediate post‐test.

**Figure 4:**
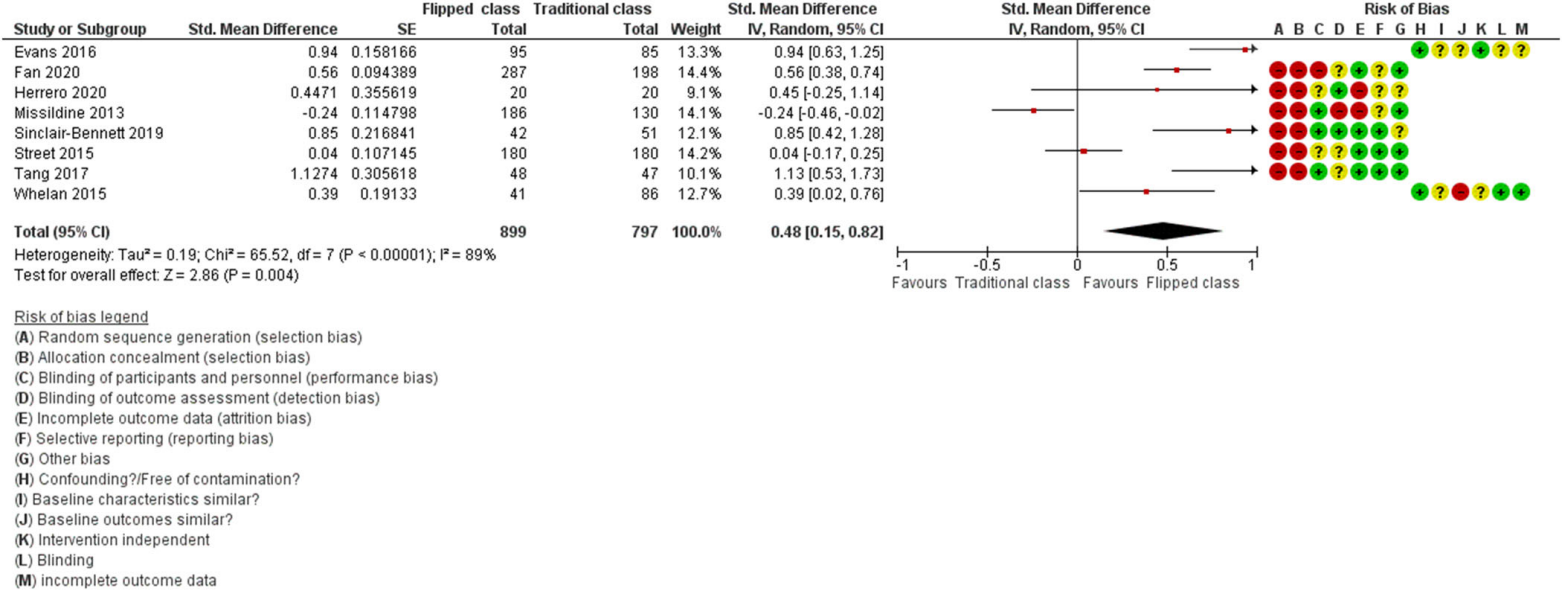
(Analysis 2.1) Forest plot showing the results of students' satisfaction.

**Figure 5:**
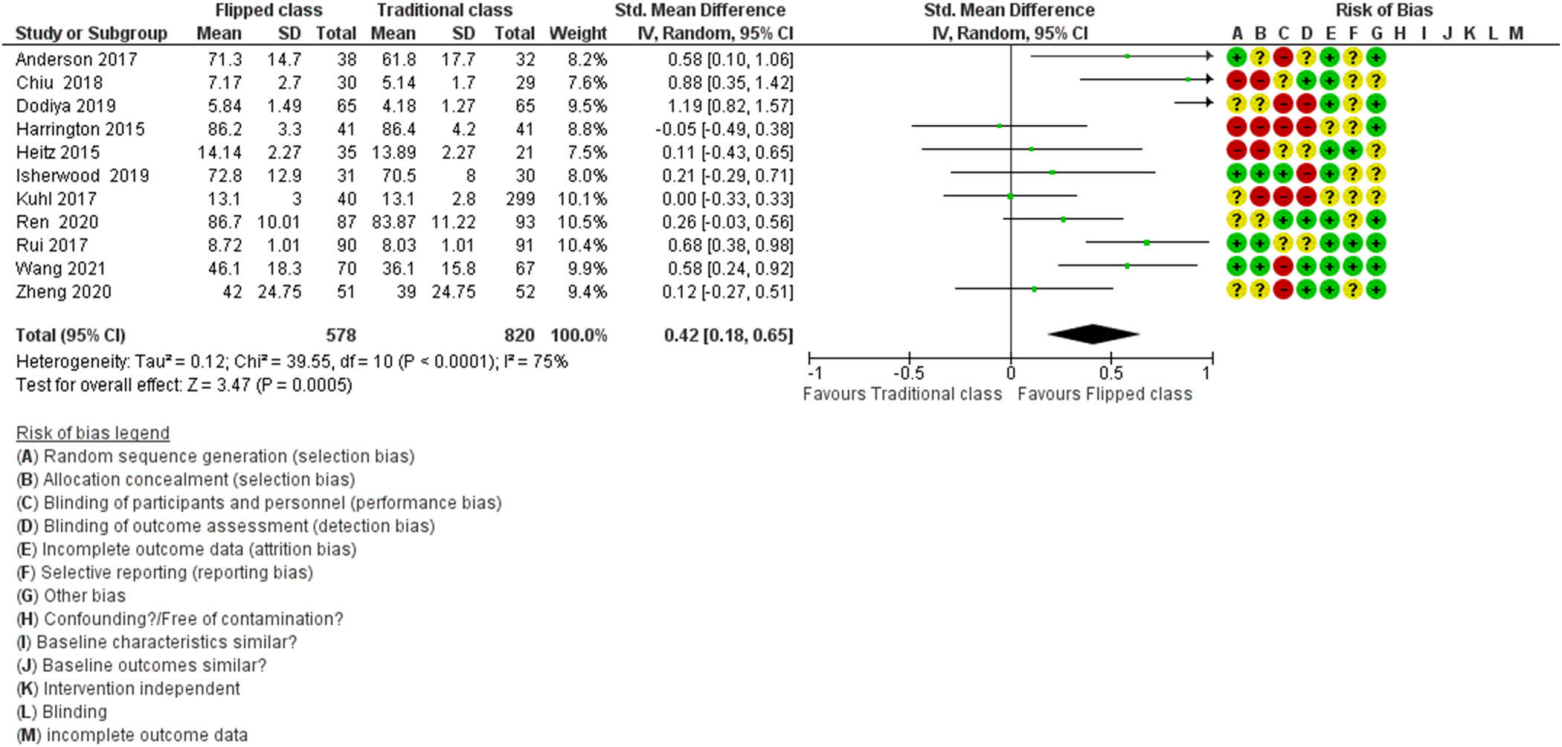
(Analysis 3.1) Forest plot showing the results of academic performance in 11 randomised controlled trials.

###### Two‐group observational design (Analysis 3.3)

Of 15 observational studies with two‐group comparisons, 14 studies (Bossaer, 2016; Boysen‐Osborn, 2016; Burak, 2015; Chaudhuri, 2019; Cheng, 2016; Cotta, 2016; Evans, 2016; Gillispie, 2016; Morton, 2017; O'Connor, 2016; Stewart, 2013; Whelan, 2015; Wilson, 2016; Wong, 2014) reported comparable examination scores in the two groups (SMD: 0.81, 95% CI: −0.23, 1.85, 14 studies, *n* = 2523). Heterogeneity was substantial (*τ* ^2^ = 3.87, *p* < 0.00001, *I* ^2^: 99%). The remaining study did not report this outcome (Evans, 2016).

These analyses suggested that there was a relationship between study design and effect size, such that experimental, randomised designs tend to yield smaller effect sizes, compared to non‐randomised designs.

##### Facilitators (enabling factors) and barriers

Only a limited number of studies reported detail relating to barriers and facilitators, with variations in descriptions (Supporting Information: Appendix 4).

One study highlighted that an effective flipped class model required ‘course facilitators being qualified’ (Chiu, 2018). In this study all programme facilitators were qualified by Taiwan Evidence‐Based Medicine Association, making it easier to create acceptable content and prepare relevant questions.

On the other side, the barriers most encountered in the reported studies were concerns over Internet accessibility (Angadi, 2019; Bossaer, 2016). Also, the time factor was another concern (Bossaer, 2016). For instance, students commented…‘did not have enough time to listen to lectures before coming to class’ *(*Bossaer, 2016) Another concern was the adequacy and quality of the study material provided to the students (Baris, 2020; Bossaer, 2016; Chaudhuri, 2019).

#### 5.3.2 Sensitivity analysis (Analysis 4.1)

After the removal of eleven studies with imputed data from the original analysis of 44 studies, the overall academic performance was retained: higher in the flipped class group compared to the traditional class group (SMD: 0.54, 95% CI: 0.24, 0.85, 33 studies, *n* = 5924, *τ* ^2^ = 0.76, *p* < 0.00001, *i* ^2^: 97%). The SMD of 0.54 can be interpreted as a moderate effect size. Heterogeneity was substantial. Qualitatively, the direction of effect size was the same. The magnitude of the effect changes slightly but there is considerable overlap between the uncertainty estimates (the confidence intervals) of the main analysis and the sensitivity analysis.

This reflects that data imputations have no serious impact in this review on the effect estimates.

#### 5.3.3 Assessment of reporting biases

This section below reports findings of publication bias by visualising the funnel plot asymmetry.

**Figure 6:**
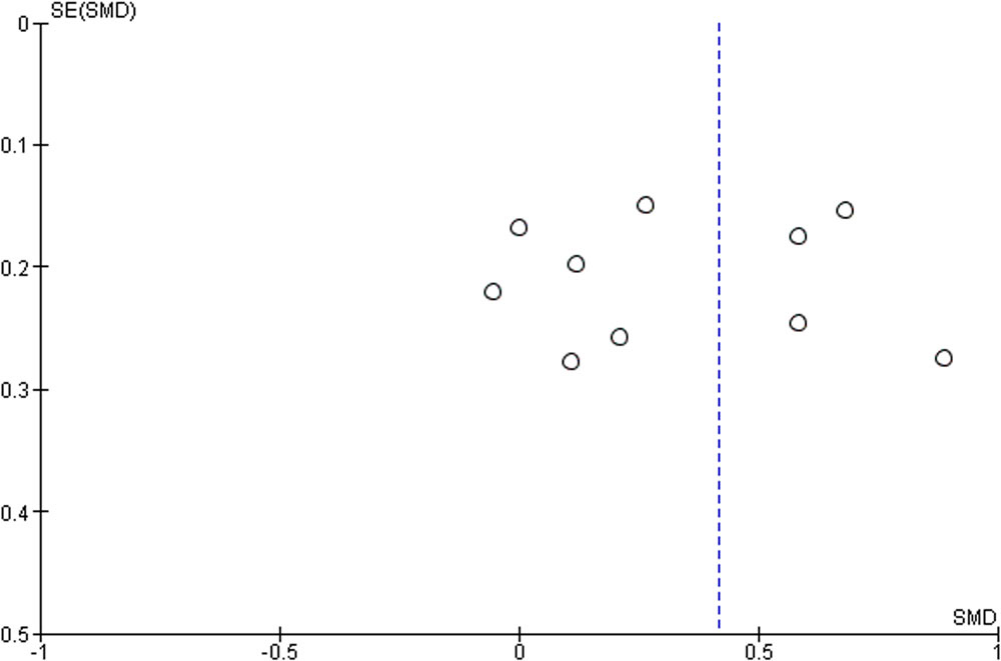
(Analysis 3.1) Funnel plot showing the likelihood of publication bias.

Figure 6 displays a funnel plot on academic performance measured with examination scores/grades by RCT (11 studies). The effect sizes are shown on the *X*‐axis, while Standard errors are shown on the *Y*‐axis. There were no clear signs of asymmetry.

Based on the required number of studies and adequate data sets, we investigated publication bias only on examination scores pertinent to the RCT design. We found funnel plot symmetry, indicating an absence of publication bias. However, our interpretation is limited to direct evidence of publication bias or the lack thereof. We, therefore, were cautious in the interpretation of our results.

## 6 DISCUSSION

### 6.1 Summary of main results

Our main objective was to assess the effectiveness of flipped classroom intervention for undergraduate health professional education students on academic performance and course satisfaction. In total, this review included 45 studies with a total of 8426 participants comprising 11 RCTs, and 34 non‐RCTs (19 QES, 15 Observational designs).

Participants in these studies were undergraduate students from various health professional pathways with Medicine being the most common program pathway. The majority of the studies were conducted in high‐income countries such as the USA with only three studies from low‐and‐middle‐income countries such as India. Flipped class contexts were heterogeneous across included studies.

The results from 44 studies could be pooled in a single meta‐analysis for an outcome of academic performance, and eight studies for student satisfaction. All the meta‐analyses showed better academic performance and higher satisfaction in the flipped class of learning.

Studies mostly reported the examination scores/grades (44/45, 97.8%), but only a few studies assessed student satisfaction with the methods of learning (8/45, 17.7%). Outcomes were measured mostly in the mean difference between the two methods of learning with the use of various assessment tools including MCQs, OSCE, quizzes, Likert scales, and other tools that are less common or more specialised in context (Supporting Information: Appendix 2).

A caveat was that moderator analysis with potential factors (e.g., school setting, semester, course contents, previous achievement, and delivery time) was not done in this review. This was because lack of sufficient information on these potential factors reported by the included studies. These additional moderators should be considered and included in future reviews. Even though increasing the number of moderators might help in reducing confounding, doing so may reduce the statistical power of the analysis of the additional moderators do not significantly explain the observed variation. Including many moderators may also cause multicollinearity (Dietrichson, 2021).

Outcomes were generally measured and then compared with the two methods of learning after the interventions. After the variations in programme pathways, population characteristics, intervention context, measures of outcome assessments, and the tools used for assessments across included studies, substantial heterogeneity was observed, as expected. For instance, even within the same programme pathway, the tools used in the Medicine programme ranged from the commonly used MCQ, and OSCE to special tools such as OSATS (Chiu, 2018). In the nursing pathway, more complex tools such as the SE‐EBP scale (Chu, 2019) and Ricketts' Critical Thinking Disposition Inventory (Dehghanzadeh, 2020) were used in the included studies. Furthermore, the baseline characteristics of participants varied across included studies. For instance, participants included ranged from novices (Year 1), and sophomores (Years 2, 3) to exit level (>3 years, the final year) at their learning institutes (Supporting Information: Appendix 2).

### 6.2 Overall completeness and applicability of evidence

Related to the implications of the findings of this review for educational practice, several issues need to be considered.

Although we conducted an extensive search to find relevant studies, there might still be some gaps that remain. While large subject and interdisciplinary databases were used, it's certainly possible that relevant studies were missed, especially if they were not widely indexed. The search terms were all in English which does not preclude discovery of non‐English published research, but future reviews could be more intentional about searching with non‐English search terms. While grey literature sources were targeted, education grey literature (e.g., untranslated, unindexed reports) can be challenging to find and retrieve.

The heterogeneity of findings among these studies might be attributed to different subjects or course designs. Almost all the included studies did not explain the correlation among different domains in Bloom's taxonomy of learning objectives from flipped classes (Wu, 2018). Hence, the explicit effectiveness of the flipped class method is still a concern. Moreover, the studies included belonged to a single context of students from a particular cohort in a particular year in an undergraduate curriculum studying a particular subject (Issenberg, 2005). Our findings, therefore, cannot be generalised to other contexts, such as students in other year cohorts or specialties. Published non‐Campbell systematic reviews on the outcomes of the flipped class method have reported that such outcomes are often *not* generalisable (Chen, 2017; Issenberg, 2005). Knowledge‐based scores (e.g., MCQ) and skill‐based scores (e.g., OSCE) are only helpful for evaluating academic achievement in the short term, which is limited in determining effectiveness in the long‐term.

In summary, the applicability of the evidence of this review to current practice in undergraduate health professional education is limited, and the generalisability of the findings should be interpreted with caution.

### 6.3 Quality of the evidence

We have summarised the certainty of evidence in Summary of findings Table 1.

The GRADE assessment showed low‐certainty evidence for both academic performance outcomes and students' satisfaction. The evidence suggests our confidence in the effect estimate of academic performance, and students' satisfactions are limited, and the true effect may be substantially different from the estimate of the effect.

Many of the included studies have not mentioned pre‐published protocols and analysis plans. Therefore, whether there was selective reporting or not is a concern. Information about how the random sequence was generated was lacking in most RCTs, and the randomisation procedure was often sparsely described. As this information is easy to include, this is an area where the reporting of studies can be improved.

Confounding may have occurred during the interventions such as if the teachers were involved in the assessment of both intervention and control, they may affect the outcome.

Blinding was a concern in almost all included studies. Complete blinding is difficult to achieve in educational research, but, for example, it is possible to use investigators that are blinded to intervention status. In several included studies, students self‐reported and were not blinded. Moreover, both groups were administered the same program at the same institution, leading to the assumption that cross‐contamination may have occurred (Fan, 2020). Lack of blinding could contribute to the bidirectional bias of the results in favour of the intervention group as well as in favour of the control group. For example, if students are well aware of participating in an experiment (flipped class approach in this case) and work harder—that is, a *Hawthorne effect*. If so, the beneficial effects are overestimated. On the other side, students in the control group (i.e., traditional class) may also work harder because they were aware that they did not get the intervention or/and they wanted to compete with the other group—that is the *John Henry effect*. In that case, the beneficial effects of flipped classes are underestimated. Moreover, if attrition by comparatively low‐achieving students in the intervention group (flipped class group) is more common, then the effects in this meta‐analysis would be overestimated.

If not all, many observational studies did not provide justifications on why one group of students was assigned to the intervention group and another to the control group. It was, therefore, difficult to assess the risk of selection for the intervention. It is not certain whether included effect sizes were reasonably well balanced on observable confounders though. Moreover, faculty members on both learning approaches (flipped class and traditional teaching or experimental group and controlled group) could have communicated or shared their teaching strategies, which may have influenced the results (Fan, 2020).

### 6.4 Potential biases in the review process

This review was based on a published protocol (Naing, 2019) and any deviations from the published protocol are noted in the section on differences between the protocol and review. Incomplete identification of studies for this review is unlikely as we performed a comprehensive search of databases, websites, trial registries, and reference lists. However, there are areas that may have introduced bias into the review.

First, there might be bias in the review process, for example, the screening or data extraction processes, although we had put maximum efforts to be comprehensive. Second, we contacted the authors for missing information for details of study characteristics and/or clarification on data. We did not receive replies. As described in a published meta‐analysis, we did not know how they would have influenced the estimates, albeit with no reason to suspect a systematic bias from these missing studies (Lag, 2019). Third, the most frequent studies were from the USA, an English Speaking country. We may also have missed studies from European countries where languages other than English are used. Moreover, many studies were from high‐income countries such as China and USA. Limited connectivity to the internet and access to databases are challenges that will need to be considered when implementing flipped class teaching in the low‐and‐ middle‐income countries. As learning does not occur in a vacuum, it is essential to take into consideration the context within which learning takes place (Rohwer, 2017). Fourth, the concurrent use of two learning models in the same semester is one potential limitation of this review. The possibility that students in the two conditions shared materials cannot be discounted (Anderson, 2017). In some studies, a combination of the flipped class and another teaching method (e.g., PBL) was compared with the traditional class (Hu, 2019), and there was no separate data for the flipped class alone. Hence, higher or lower effect estimates of a flipped class are a concern. Fifth, there were different traditional learning' conditions across the primary studies, and these may also affect the results. For instance, it is anticipated that the more active the students involved in the traditional class group are, it is likely that there will be a smaller difference with the flipped classroom group.

### 6.5 Agreements and disagreements with other studies or reviews

A systematic review of students in pharmacy education, incorporating six observational studies with 1395 participants reported no significant difference in final examination scores (i.e., academic performance in the present review) comparing the two educational models (MD: 2.90, 95% CI: −0.02‐5.81, *p* = 0.05). There was substantial heterogeneity among the studies included (*I* ^2^: 91%) (Gillette, 2018). Although the exact reasons were not known, this could be attributed to the concerns about faculty time and resources (McLaughlin, 2014) as well as student time for preparation (Gillette, 2018). In this sense, a study reported that to flip a class, a professor would have to invest 127% more time in course development and management. After initial development, the flipped classroom requires 57% more time to maintain when compared to a lecture course (McLaughlin, 2014). From the findings of this review, it is difficult to demonstrate evidence to support flipped class method of learning. That is not to suggest they are inappropriate, merely the fact that there is still a paucity of well‐designed randomised controlled trial data to guide this key area. A meta‐analysis incorporating 28 studies in a variety of disciplines (i.e., medicine, pharmacy, nursing, and so on) reported that there was no significant variation when comparing studies with different research designs (Hew, 2018). With the magnitude measured in this way, the effect sizes found in our review were larger than comparable effect sizes from a previous review in the same field (Hew, 2018). Thus, the results of this review provide support for trying out flipped class interventions for undergraduate health professional students.

## 7 AUTHORS' CONCLUSIONS

### 7.1 Implications for practice

Based on the low certainty evidence of this review, the flipped class approach may increase or reduce academic performance, and students satisfaction among health professional undergraduate students.

There is speculation that traditional assessment methods may not accurately reflect gains from the flipped classroom, which may cause the reported effect to be underestimated (Gillette, 2018). This is because the flipped classroom is designed to develop higher order thinking in students and, as such, graded assessments (e.g., open text, essay, etc.) should provide students the opportunity to demonstrate the development of these skills. Moreover, for flipped learning, assessment should be used to hold students accountable for pre‐class learning such as guided questions for pre‐class material. This will further act as a mechanism for encouraging students to learn foundational material before coming to the (flipped) class (Persky, 2017).

The literature shows that students report satisfaction being receptive to the concept of the flipped classroom, but there were concerns (e.g., workload and lack of time to prepare) that were consistently reported by students across many studies. To implement a flipped class in the curriculum development continuum, it is worth remembering that pre‐qualification flipped class can be regarded as an investment in the future.

Students were likely unhappy to do work at home that was traditionally done in a face‐to‐face class format, and they may have considered watching the pre‐class videos as time pressure (Hew, 2018). Concerning theoretical variables in UTA (David, 1989) and UTAUT (Venkatesh, 2003), if a flipped classroom is user‐friendly and the learning environment facilitates their learning, then it will promote students' engagement, interactions, and cooperation in learning, which will further improve their performance. Hence, instructors who wish to employ flipped classrooms should first promote students' understanding of this new instructional approach by explaining the rationale, and potential benefits of the flipped classroom and consider limiting the total length of all combined video segments to about 20 min (Hew, 2018).

### 7.2 Implications for research

Despite the quantity of research output on the flipped classroom as an instructional strategy, most of the studies did not employ a rigorous design. When planning future trials of the flipped classroom, attention should be given to the following aspects, which would improve evidence‐based information: rigorous randomisation procedures and larger sample sizes. Importantly, studies should include at least one common outcome to enable a formal summation of the evidence. A description of pre‐publishing trial protocols and analysis plans is desirable to reduce researcher bias and promote transparency. More research studies using prospective, randomised designs with larger classes should be conducted before the widespread adoption of this teaching methodology. Due to a lack of evidence on the impact of flipped classes on resources (e.g., costs and benefits), attention is needed in this area.

## CONTRIBUTIONS OF AUTHORS

**Content**: CN, MAW, and DKC

**Systematic review methods**: CN, MAW, and HHA

**Statistical analysis**: CN, DKC, and HHA

**Information retrieval:** Amy Riegelman

## DECLARATIONS OF INTEREST

CN: none known

MAW: none known

HHA: none known

DKC: none known

AR: none known

## DIFFERENCES BETWEEN PROTOCOL AND REVIEW

Due to limited institutional access, we did not conduct a hand‐search in the American Educational Research Journal and Journal of Educational Research. We did not search CINAHL because we were confident that items indexed in CINAHL would have been located in many other databases and search engines that were utilised. We did not search ‘Education Research Global Observatory’ (http://ergo.asu.edu/ejdirectory.html) as the directory is no longer working during both search periods. Wayback Machine was used to further investigate why the page hasn't worked in several years. According to our protocol, we contacted the authors for clarification of data or missing data. We did not receive a reply. Our protocol stipulated that we would use intention‐to‐treat (ITT) estimates whenever available. However, the included studies did not report ITT estimates. We could not find cluster‐level RCTs, natural experiments, or regression discontinuity designs, as planned. In this review, we reported the combined analysis (combining the data from all disciplines) for the primary outcome (academic performance, student satisfaction) and then the secondary outcome (moderating effects according to study design). In the protocol, we have wrongly indicated student satisfaction as a secondary outcome, although this was linked to primary research question. We have corrected this error and regarded student satisfaction as the primary objective/outcome.

## PUBLISHED NOTES

**Characteristics of studies**

**Characteristics of included studies**

Anderson 2017

**Table:** 

**Methods**	RCT, two groups parallel design
**Participants**	1st year students in PHAR 541 course *N* = 70 (FC: 38 vs. TC: 32) Males, n (%): FC, 18(47.4); TC,14(43.7) Age in year, mean(±SD): FC 27.3 (±5.5); TL 26.6 (±6.7) Inclusion criteria: All students enroled within the PHAR 541 course (*n* = 578) were eligible Exclusion criteria: Not described
**Interventions**	**Intervention:** Flipped classroom (FC) (*n* = 38) Prework with readings, recorded lectures, performance of guided tasks or other activities developed by the instructor **Control/Comparator:** traditional lecture (TL) (*n* = 32) Course delivered in 16‐h pharmacy calculations education (a 5‐week course of instruction) in PHAR 541 course
**Outcomes**	Students' performance on basic pharmaceutical calculations (OSCE); Regression analysis on primary independent variable and the demographic variables
**Notes**	Setting: Marshall University School of Pharmacy; Ethics approval: Obtained Funding: Not mentioned Study period: Not stated

Risk of bias table

**Table:** 

Bias	Authors' judgement	Support for judgement
Random sequence generation (selection bias)	Low risk	Block randomisations: Block strata by quartile of student performance on the Pharmacy College Admission Test (PCAT) Quantitative domain.
Allocation concealment (selection bias)	Unclear risk	Insufficient information Quote: ‘Students were randomly assigned to one of two educational conditions’. p. 3
Blinding of participants and personnel (performance bias)	High risk	Quote: ‘the same instructors (study investigators) were assigned to teach bothcourse sections’ p. 3
Blinding of outcome assessment (detection bias)	Unclear risk	Insufficient information Quote: ‘both course sections met on the same day of each week; and the same posted course materials’. p. 3
Incomplete outcome data (attrition bias)	Low risk	No withdrawal
Selective reporting (reporting bias)	Unclear risk	protocol not available
Other bias	Low risk	None
Confounding?/Free of contamination?	Unclear risk	
Baseline characteristics similar?	Unclear risk	
Baseline outcomes similar?	Unclear risk	
Intervention independent	Unclear risk	
Blinding	Unclear risk	
incomplete outcome data	Unclear risk	

Angadi 2019

**Table:** 

**Methods**	Quasi‐experimental
**Participants**	2nd year medical undergraduate students in India *N* = 98 (FC, 49 vs. TC,49) Male, *n* (%): Not mentioned Age in years: Not mentioned Inclusion/exclusion criteria: not stated
**Interventions**	**Interventio**n: Flipped classroom (FC) **Control/Comparator**: conventional small group teaching (TC) Topic is ‘Drugs acting on cardiovascular system’ in pharmacology course
**Outcomes**	Students' performance on pre‐and post test (MCQ) Mean scores of the end of module test (short essay type question) Students' perceptions to flipped classroom
**Notes**	Setting: The J. N. Medical College, Belagavi. India; Ethic approval: Obtained. Funding: Self‐funded study. Study period: 06/2018–12/2018

Risk of bias table

**Table:** 

Bias	Authors' judgement	Support for judgement
Random sequence generation (selection bias)	High risk	Quote: ‘Randomly selected’. p. 2; details not described.
Allocation concealment (selection bias)	High risk	Quote: ‘two groups by Lot method’. p. 2; details not described
Blinding of participants and personnel (performance bias)	Unclear risk	Insufficient information
Blinding of outcome assessment (detection bias)	Unclear risk	Insufficient information
Incomplete outcome data (attrition bias)	Low risk	No withdrawal
Selective reporting (reporting bias)	Unclear risk	Protocol not available
Other bias	Low risk	None
Confounding?/Free of contamination?	Unclear risk	
Baseline characteristics similar?	Unclear risk	
Baseline outcomes similar?	Unclear risk	
Intervention independent	Unclear risk	
Blinding	Unclear risk	
incomplete outcome data	Unclear risk	

Baris 2020

**Table:** 

**Methods**	prospective controlled post‐ test study (Quasi‐experiment)
**Participants**	2nd year medical students; *N* = 363 (FC 183 vs LBL 180) Male, *n* (%): FC, 95 (52); LBL, 93 (52) Age in years: 19–21 years: FC 67%, LBl 65%; (22–24): FC 33%, LBL 35%
**Interventions**	**Intervention** = Flipped classroom (FC) **Control/comparator** = Lecture‐based (theoretical class) (LBL)
**Outcomes**	Performance test on skill performance in OSCE; Persistence of skill performance; Students' feedback
**Notes**	Setting: Faculty of Medicine at the Hacettepe University in Turkey. Ethics approval: Not applicable (Informed consent was taken of participants Funding: Not mentioned Study period: one semester (Spring semester of 2017–2018)

Risk of bias table

**Table:** 

Bias	Authors' judgement	Support for judgement
Random sequence generation (selection bias)	High risk	Insufficient information; Quote: ‘small groups are created random’. p. 3
Allocation concealment (selection bias)	High risk	Based on previous academic achievement, gender, age; did not control for variability in teaching skill of the teachers for FC and LBL
Blinding of participants and personnel (performance bias)	High risk	Students were informed about their assigned method of teaching
Blinding of outcome assessment (detection bias)	High risk	The same lecturers, and the practices were assessed by the same 8 trainers
Incomplete outcome data (attrition bias)	Unclear risk	Not stated
Selective reporting (reporting bias)	Unclear risk	No protocol available
Other bias	Low risk	None
Confounding?/Free of contamination?	Unclear risk	
Baseline characteristics similar?	Unclear risk	
Baseline outcomes similar?	Unclear risk	
Intervention independent	Unclear risk	
Blinding	Unclear risk	
incomplete outcome data	Unclear risk	

Bossaer 2016

**Table:** 

**Methods**	A design experiment (See below)
**Participants**	3rd year pharmacy students in oncology module in 2012 and 2013 *N*: 146, (FC,76; TC,72) Male, *n* (%): not mentioned Age in years: not mentioned Inclusion/exclusion criteria: not described
**Interventions**	**Intervention**: Flipped classroom in Year 2013(FC) in 2013 batch ‐to watch video pod casts before in class case studies **Control/comparator**: Interactive lecture in 2012 batch (TC) ‐ large classroom setting, with optional case studies as supplemental homework
**Outcomes**	Student performance in pharmacotherapy oncology module: End‐of‐module examination (60 MCQ) Oncology module examination scores Undergraduate GPA COP GPA Pharmacotherapy series GPA Total PCAT scores
**Notes**	Setting: East Tennessee State University (ETSU) Bill Gatton College of Pharmacy, Tennessee, USA Ethical approval: Obtained. Funding: Rasht Islamic Azad University, Iran (grant #. 1179508260009) Study period: 2012 and 2013 **A design experiment**: The term was introduced in 1992 as a method to conduct formative research and refine educational designs based on principles derived from prior research. Design experiments are set in the messy situations that characterize real‐life learning; to avoid the distortions of laboratory experiments and therefore constitute a means of addressing the complexity that is a hallmark of educational settings (Piercea, 2012a).

Risk of bias table

**Table:** 

Bias	Authors' judgement	Support for judgement
Random sequence generation (selection bias)	Unclear risk	
Allocation concealment (selection bias)	Unclear risk	
Blinding of participants and personnel (performance bias)	Unclear risk	
Blinding of outcome assessment (detection bias)	Unclear risk	
Incomplete outcome data (attrition bias)	Unclear risk	
Selective reporting (reporting bias)	Unclear risk	
Other bias	Unclear risk	
Confounding?/Free of contamination?	Unclear risk	Different cohorts, but the same materials used
Baseline characteristics similar?	Unclear risk	Demographic variables are different in two groups
Baseline outcomes similar?	Low risk	Cumulative GPA in previous pharmacotherapy courses, College Admissions Test (PCAT)
Intervention independent	Low risk	Different cohorts
Blinding	Unclear risk	insufficient information
incomplete outcome data	High risk	low response rate (39.4%)

Boysen‐Osborn 2016

**Table:** 

**Methods**	case‐control study, (historical control classes)
**Participants**	Final year medical students in advanced cardiac life support (ACLS) course *N* = 354 (FC/TBL,95 vs. LB, 259). Age in years: Not mentioned. Male, *n* (%): Not mentioned. Inclusion: used 3 recent histological cohorts Exclusion criteria: Not described.
**Interventions**	**Intervention**; team‐based learning in 2015 (FC/TBL) **Control/comparator**: lecture‐based in 2012 (LB) i. 27.5 h of instruction for FC/TBL model ii. 20 h (12 h lecture, 8 h simulation) in LB iii. TBL covered 13 cardiac cases; LB had none iv. Seven simulation cases and didactic contents
**Outcomes**	Scores of 3 evaluation (MCQ test, cardiac rhythm test, clinical management test)
**Notes**	Setting: University of California‐Irvine School of Medicine, USA Ethical approval: Obtained (HS# 2014‐1195) Funding: Not mentioned Study duration: 2012–2014 in LB; 2015 in FC/TBL

Risk of bias table

**Table:** 

Bias	Authors' judgement	Support for judgement
Random sequence generation (selection bias)	Unclear risk	
Allocation concealment (selection bias)	Unclear risk	
Blinding of participants and personnel (performance bias)	Unclear risk	
Blinding of outcome assessment (detection bias)	Unclear risk	
Incomplete outcome data (attrition bias)	Unclear risk	
Selective reporting (reporting bias)	Unclear risk	
Other bias	Unclear risk	
Confounding?/Free of contamination?	High risk	different total instructional time (Table 1); a single instructor for the large group component of TBL.
Baseline characteristics similar?	Low risk	students of same background; Average MCAT scores: 31.8 in FC/TBL versus 32.1 in LB; Average GPA: 3.68 in both groups
Baseline outcomes similar?	Low risk	same assessment test
Intervention independent	Unclear risk	insufficient information
Blinding	Unclear risk	not stated
incomplete outcome data	High risk	Quote: ‘up to 1/3 of students apparently did not watch the pod casts at all’. p. 5

Burak 2015

**Table:** 

**Methods**	Observational study
**Participants**	Medical students in hepatology course *N* = 338 (FC, 163 vs. TL, 175) Age in years: Not mentioned Male, *n* (%): Not mentioned Age in years: Not mentioned Inclusion/exclusion criteria: Not described
**Interventions**	**Intervention**: Flipped class in 2014 (FC) **Control/Comparator:** Traditional class in 2012 (TL)
**Outcomes**	Exam performance Student satisfaction
**Notes**	Setting: Cumming School of Medicine in Alberty, Canada Ethical approval: Obtained. Funding: Not mentioned. Study duration: 2012 cohort and 2014 cohort Only abstract is available.

Risk of bias table

**Table:** 

Bias	Authors' judgement	Support for judgement
Random sequence generation (selection bias)	Unclear risk	
Allocation concealment (selection bias)	Unclear risk	
Blinding of participants and personnel (performance bias)	Unclear risk	
Blinding of outcome assessment (detection bias)	Unclear risk	
Incomplete outcome data (attrition bias)	Unclear risk	
Selective reporting (reporting bias)	Unclear risk	
Other bias	Unclear risk	
Confounding?/Free of contamination?	Low risk	6 h independent study time
Baseline characteristics similar?	Unclear risk	Not stated, only abstract is available
Baseline outcomes similar?	Low risk	Same to both groups
Intervention independent	Unclear risk	Insufficient information, only abstract is available
Blinding	Unclear risk	Not stated
incomplete outcome data	Unclear risk	Insufficient information, only abstract is available

Chaudhuri 2019

**Table:** 

**Methods**	Cohort study
**Participants**	1st year medical student at Department of Physiology *N*: 120 (10 FC class vs. 10 TC class, number in each group not mentioned) Male, *n* (%): Not mentioned Age in years: Not mentioned Inclusion criteria: All students enroled in the first MBBS programme were included. Ten lecture classes Exclusion criteria: Not described.
**Interventions**	**Intervention**: Flipped class (FC) **Control/comparator**: Traditional class (TC) (10‐lecture classes assisted by FC vs. 10‐traditional lecture class)
**Outcomes**	Exam score of 10 MCQ (post‐ sessions)
**Notes**	Setting: Medical College of West Bengal, India Ethics approval: Obtained. Funding: Not mentioned Study period: Not mentioned

Risk of bias table

**Table:** 

Bias	Authors' judgement	Support for judgement
Random sequence generation (selection bias)	Unclear risk	
Allocation concealment (selection bias)	Unclear risk	
Blinding of participants and personnel (performance bias)	Unclear risk	
Blinding of outcome assessment (detection bias)	Unclear risk	
Incomplete outcome data (attrition bias)	Unclear risk	
Selective reporting (reporting bias)	Unclear risk	
Other bias	Unclear risk	
Confounding?/Free of contamination?	High risk	Students from the same school
Baseline characteristics similar?	Unclear risk	10 FC class vs. 10 TC class, number in each group not mentioned.
Baseline outcomes similar?	Low risk	Same assessment tests used
Intervention independent	Low risk	Different teaching methods
Blinding	Unclear risk	Not stated
incomplete outcome data	Unclear risk	Quote: ‘Most of the students (98%) did not pay attention to the study materials provided to them before their classes’. p. 577

Cheng 2016

**Table:** 

**Methods**	Cohort study
**Participants**	Traditional Chinese Medicine program in 2014 *N* = 111 (FC, 24 vs. TC, 87) Male, n (%): FC 10 (41.7%); TC 33(37.9) Age in years: not mentioned Inclusion/exclusion criteria:
**Interventions**	Intervention: Flipped class (FC) Control/comparator: Conventional/traditional class (TC)
**Outcomes**	Test scores (MCQ) Questionnaire to FC
**Notes**	Setting: Jinan University, China Ethics approved—Obtained from the Jinan University Imbalance sample size

Risk of bias table

**Table:** 

Bias	Authors' judgement	Support for judgement
Random sequence generation (selection bias)	Unclear risk	
Allocation concealment (selection bias)	Unclear risk	
Blinding of participants and personnel (performance bias)	Unclear risk	
Blinding of outcome assessment (detection bias)	Unclear risk	
Incomplete outcome data (attrition bias)	Unclear risk	
Selective reporting (reporting bias)	Unclear risk	
Other bias	Unclear risk	
Confounding?/Free of contamination?	High risk	Quote: ‘all of the FC students in this study were volunteers’. p. 9
Baseline characteristics similar?	Low risk	students from same background, same content
Baseline outcomes similar?	Low risk	the same examination papers at the end of the semester; The papers were graded by the same teachers
Intervention independent	Unclear risk	insufficient information
Blinding	High risk	Quote: ‘The papers were graded by the same teachers, thus allowing for direct comparison of the learning outcomes’. p. 4
incomplete outcome data	Unclear risk	two groups of imbalance sample size; Quote: ‘All of the FC students in this study were volunteers’.

Chiu 2018

**Table:** 

**Methods**	RCT
**Participants**	3rd year medical students in a 1‐hour laparoscopic skill training session *N* = 59 (FC, 30 vs. TC, 29) Male, *n* (%): FC, 22 (73.3); TC,18 (62) Age in years: Not mentioned **Inclusion criteria**: 6th year medical students(=3rd year in US system), who had no previous laparoscopic suturing experiences **Exclusion criteria**: Not described
**Interventions**	**Intervention**: Flipped classroom (FC) **Control/comparator**: Conventional/traditional class (TC)
**Outcomes**	performance in laparoscopic suturing and intracorporeal knot‐tying using modified OSATS mean satisfaction scores
**Notes**	Setting: Taipei Medical University Hospital, an academic teaching hospital Ethical approval: Obtained Funding: Not mentioned Study period: Not mentioned OSATS: Objective structured assessment of technical skills tool

Risk of bias table

**Table:** 

Bias	Authors' judgement	Support for judgement
Random sequence generation (selection bias)	High risk	Assignment of participants was based on their registered order
Allocation concealment (selection bias)	High risk	Quote: The former half students (*n* = 29)were allotted to ‘the conventional group’ and the latter half (*n* = 30) were to ‘the flipped group’. p. 327
Blinding of participants and personnel (performance bias)	Unclear risk	insufficient information
Blinding of outcome assessment (detection bias)	Low risk	Quote ‘Instructors were blinded to the grouping of the students’. p. 327
Incomplete outcome data (attrition bias)	Low risk	No withdrawal/no missing data
Selective reporting (reporting bias)	Unclear risk	Protocol not available
Other bias	Unclear risk	Quote: ‘recruited voluntarily’. p. 327
Confounding?/Free of contamination?	Unclear risk	
Baseline characteristics similar?	Unclear risk	
Baseline outcomes similar?	Unclear risk	
Intervention independent	Unclear risk	
Blinding	Unclear risk	
incomplete outcome data	Unclear risk	

Chu 2019

**Table:** 

**Methods**	quasi‐experimental design with nonequivalent control group
**Participants**	nurses enroled for EBN in‐service training course at medical center in northern Taiwan, aged>20 yrs *N* = 151 (FC, 75 vs. TC, 76) Male, *n* (%): FC, 2 (2.63); TC, 1(1.33) Age in years, mean (±SD): FC, 35.2 (±9.19); TC 33.61(±8.5); **Inclusion criteria**: i. employed nursing staff, ii. aged > 20 years, iii. willing to sign a consent form to participate in the study. Exclusion criteria:not described
**Interventions**	**Intervention**: Flipped classroom (FC) **Control/comparator**: Traditional classroom (TC)
**Outcomes**	Pre‐course, post‐course, and one month after the course (Based Nursing knowledge scale) Self‐efficacy in EBP scale
**Notes**	Setting: Medical center in northern Taiwan Ethics approval: Obtained (IRB # 106‐0828C) Funding: Chang Gung Memorial Hospital (MOST NMRPG3F0541) Study period: Not mentioned EBN: Evidence‐based nursing EBP: Evidence‐based practice (SE‐EBP)

Risk of bias table

**Table:** 

Bias	Authors' judgement	Support for judgement
Random sequence generation (selection bias)	High risk	convenient sampling
Allocation concealment (selection bias)	High risk	Quote: ‘the first 75 nurses were assigned to the control group and the following 76 nurses were assigned’. p. 4
Blinding of participants and personnel (performance bias)	Unclear risk	Not stated
Blinding of outcome assessment (detection bias)	Unclear risk	Not stated
Incomplete outcome data (attrition bias)	Low risk	No missing data; no withdrawal
Selective reporting (reporting bias)	Unclear risk	No protocol available
Other bias	Low risk	None
Confounding?/Free of contamination?	Unclear risk	
Baseline characteristics similar?	Unclear risk	
Baseline outcomes similar?	Unclear risk	
Intervention independent	Unclear risk	
Blinding	Unclear risk	
incomplete outcome data	Unclear risk	

Cotta 2016

**Table:** 

**Methods**	Cohort study
**Participants**	Pharmaceutical calculations course N = 316 (FC 151 vs. TL 165) Age in years: Not mentioned Male, *n* (%): Not mentioned Inclusion/exclusion criteria: Not described
**Interventions**	**Intervention**: Flipped classroom (FC) (2011 and 2012) **Control/comparator**: Traditional lecture (TL) (Section II, 2011) 2 h/week **×** 10‐week course
**Outcomes**	Students' performance: Final exam score (short answer or fill in the blank questions) Students' satisfaction to the course
**Notes**	Settings: South University School of Pharmacy(SUSOP) in Georgia, USA Ethic approval: Obtained Funding: Not mentioned Study period: 2011–2012

Risk of bias table

**Table:** 

Bias	Authors' judgement	Support for judgement
Random sequence generation (selection bias)	Unclear risk	
Allocation concealment (selection bias)	Unclear risk	
Blinding of participants and personnel (performance bias)	Unclear risk	
Blinding of outcome assessment (detection bias)	Unclear risk	
Incomplete outcome data (attrition bias)	Unclear risk	
Selective reporting (reporting bias)	Unclear risk	
Other bias	Unclear risk	
Confounding?/Free of contamination?	High risk	Different facilitators for section I and II
Baseline characteristics similar?	Low risk	Same content, students of same background
Baseline outcomes similar?	Low risk	Same test
Intervention independent	Unclear risk	Not stated
Blinding	Unclear risk	Not stated
incomplete outcome data	Unclear risk	Not stated

Dehghanzadeh 2020

**Table:** 

**Methods**	Quasi‐experimental study, nonequivalent control group
**Participants**	2nd year bachelor's nursing students under the musculoskeletal (MSK) and medical‐surgical nursing theoretical training course *N* = 85 (FC, 43 vs. TL, 42) Male, *n* (%): FC, 8 (18.6); TL, 6(14.3) in TL Age in years, mean (±SD): Fc, 19.77 (1.52); TL, 19.98 (1.15) **Inclusion criteria:** Signing up for MSK Medical‐Surgical Nursing theoretical training course, No previous experience of FC‐based learning
**Interventions**	**Intervention**: Flipped classroom (FC) **Comparator/control**: Traditional lecture (TL) Divide at 1st semester into two 42‐ and 43‐student groups and attended separate theoretical training classes 120‐minute/week, 8 weeks
**Outcomes**	Ricketts' Critical Thinking Disposition Inventory (engagement, maturity, innovativeness)
**Notes**	Setting: Nursing and midwifery Faculty of the Rasht branch of Islamic Azad University, Rasht, Iran Ethical approval: Obtained Funding:Rasht Islamic Azad University, Iran grant (no. 1179508260009). Study period: 2016

Risk of bias table

**Table:** 

Bias	Authors' judgement	Support for judgement
Random sequence generation (selection bias)	High risk	Quote: randomly allocated to either TL or FC group through coin flipping
Allocation concealment (selection bias)	High risk	Coin flipping
Blinding of participants and personnel (performance bias)	Unclear risk	Not stated
Blinding of outcome assessment (detection bias)	Unclear risk	Not stated
Incomplete outcome data (attrition bias)	Low risk	No withdrawal
Selective reporting (reporting bias)	Unclear risk	No protocol available
Other bias	Unclear risk	Not stated
Confounding?/Free of contamination?	Unclear risk	
Baseline characteristics similar?	Unclear risk	
Baseline outcomes similar?	Unclear risk	
Intervention independent	Unclear risk	
Blinding	Unclear risk	
incomplete outcome data	Unclear risk	

Dodiya 2019

**Table:** 

**Methods**	Open‐labelled interventional study
**Participants**	1st MBBS course, undergraduate medical students in the Department of Physiology *N* = 130 (FC 65 vs. TC 65) Male, *n* (%): Not mentioned Age in year, (mean ± SD): Not mentioned
**Interventions**	Intervention: Flipped class: FC Control/comparator: Traditional class: TC
**Outcomes**	Posttest exam scores Students' feedback on flip classroom
**Notes**	Setting: GMERS Medical College, Gandhinaga, India Ethics approval: ‘approval from the institutional committee’ Funding: Stated as ‘Nil’ Study period: November 2017–January 2018

Risk of bias table

**Table:** 

Bias	Authors' judgement	Support for judgement
Random sequence generation (selection bias)	Unclear risk	Need more information on Quote: ‘The study design was a open‐labeled interventional study (Education Intervention)’
Allocation concealment (selection bias)	Unclear risk	Need more information on Quote: ‘The study design was a open‐labeled interventional study (Education Intervention)’
Blinding of participants and personnel (performance bias)	High risk	Open label
Blinding of outcome assessment (detection bias)	High risk	open label
Incomplete outcome data (attrition bias)	Low risk	No withdrawls
Selective reporting (reporting bias)	Unclear risk	Can not be determined (no protocol)
Other bias	Low risk	None
Confounding?/Free of contamination?	Unclear risk	
Baseline characteristics similar?	Unclear risk	
Baseline outcomes similar?	Unclear risk	
Intervention independent	Unclear risk	
Blinding	Unclear risk	
incomplete outcome data	Unclear risk	

Evans 2016

**Table:** 

**Methods**	Cohort study with a historic control
**Participants**	1st year Stanford medical students enroled in quantitative medicine module *N* = 279(FC: 101 vs. TC: 178) Male, n (%): FC: 54 (54%); TC: NA Age in years: Not mentioned inclusion/exclusion criteria: Not stated
**Interventions**	**Intervention**: blended curriculum (flipped) in 2013 **Control/comparator** (a historic control):traditional class in 2011‐ 2012
**Outcomes**	Performance on final exam (insufficient data) Overall satisfaction
**Notes**	Settings: 3 small‐group sessions at the Stanford University, USA Ethical approval: exempted by the Stanford University institutional review board Study period: 2011–2013

Risk of bias table

**Table:** 

Bias	Authors' judgement	Support for judgement
Random sequence generation (selection bias)	Unclear risk	
Allocation concealment (selection bias)	Unclear risk	
Blinding of participants and personnel (performance bias)	Unclear risk	
Blinding of outcome assessment (detection bias)	Unclear risk	
Incomplete outcome data (attrition bias)	Unclear risk	
Selective reporting (reporting bias)	Unclear risk	
Other bias	Unclear risk	
Confounding?/Free of contamination?	Low risk	Different cohorts using historic data
Baseline characteristics similar?	Unclear risk	Not mentioned
Baseline outcomes similar?	Unclear risk	Same assessments
Intervention independent	Low risk	Blended class in a different cohort
Blinding	Unclear risk	Not stated
incomplete outcome data	Unclear risk	Not stated

Fan 2020

**Table:** 

**Methods**	Quasi‐experimental design
**Participants**	2‐year students, registered nurse‐to‐Batchelor of nursing program *N*: 485 (FC, 287 vs. TC, 198) Male, *n* (%): 20(4.1) Age in years (mean ± SD): 20.18 ± 0.59 Inclusion/exclusion: Not described
**Interventions**	**Intervention**: Flipped classroom (FC) **Control/comparator**: Traditional teaching class (TC)
**Outcomes**	pre‐post intervention scores self‐evaluated core competencies scale (SECC), meta cognitive inventory for nursing students (MINS), self‐directed learning readiness scale (SDLRS) student satisfactions
**Notes**	Setting: Nursing program at a private university in Taiwan Ethical approval: Obtained (IRB #. 104‐5709 C) Funding: 1. Ministry of Science and Technology [MOST104–2511‐S‐255‐002], 2. Administration Center of the Medical Research Department, Chang Gung Memorial Hospital, Taiwan (BMRPB80) Study period: 09/2015–02/2016

Risk of bias table

**Table:** 

Bias	Authors' judgement	Support for judgement
Random sequence generation (selection bias)	High risk	No randomisation, revealed as a quasi experimental
Allocation concealment (selection bias)	High risk	Different campus; Quote: ‘both groups were administered the same program at the same institution, cross‐contamination may have occurred’ Quote: ‘minimize intervention contamination between experimental and control group’. p. 5
Blinding of participants and personnel (performance bias)	High risk	Quote: ‘Faculty members on both campuses could have communicated or shared their teaching strategies, which may have influenced the results’.
Blinding of outcome assessment (detection bias)	Unclear risk	Not described
Incomplete outcome data (attrition bias)	Low risk	No withdrawal
Selective reporting (reporting bias)	Unclear risk	No protocol available
Other bias	Low risk	None
Confounding?/Free of contamination?	Unclear risk	
Baseline characteristics similar?	Unclear risk	
Baseline outcomes similar?	Unclear risk	
Intervention independent	Unclear risk	
Blinding	Unclear risk	
incomplete outcome data	Unclear risk	

Gillispie 2016

**Table:** 

**Methods**	Observational studies with two cohorts
**Participants**	3rd and 4th years of medical students in Obstetrics and gynaecology clerkship *N* = 70 (FC, 31 vs. TC,30) Male, n (%): FC 23 (74.1); TC 19(63.3) Age in years, (mean): FC (28.5); TC (27.9) in rotation 2 FC (28.8); TC 26.8 in rotation 3
**Interventions**	**Intervent**ion: Flipped classroom (FC) [rotation 2 and 3 of the 2015 academic year] **Control/comparator**: Traditional teaching class (TC) [rotation 2 and 3 of 2014 academic year]
**Outcomes**	Student performance (MCQ, OSCE)
**Notes**	Setting: The Ochsner Clinical School in New Orleans, LA & the University of Queensland in Brisbane, Australia Ethical approval: Not stated Funding: Not mentioned Study period: 2014–2015

Risk of bias table

**Table:** 

Bias	Authors' judgement	Support for judgement
Random sequence generation (selection bias)	Unclear risk	
Allocation concealment (selection bias)	Unclear risk	
Blinding of participants and personnel (performance bias)	Unclear risk	
Blinding of outcome assessment (detection bias)	Unclear risk	
Incomplete outcome data (attrition bias)	Unclear risk	
Selective reporting (reporting bias)	Unclear risk	
Other bias	Unclear risk	
Confounding?/Free of contamination?	Unclear risk	Not stated about facilitators;a small study
Baseline characteristics similar?	Low risk	Quote: ‘no statistical difference was observed in sex distribution or age between the comparison groups’. *p* values > 0.05 (Table 1)
Baseline outcomes similar?	Low risk	The same assessments
Intervention independent	Low risk	Different cohort in different yr
Blinding	Unclear risk	Not stated
incomplete outcome data	Low risk	According to the outcomes reported in the methodology

Grønlien 2021

**Table:** 

**Methods**	Quasi‐experimental study
**Participants**	1st semester of nursing bachelor studies in e anatomy, physiology and biochemistry (APB) course *N* = 388 (FC in 2017: 216 vs TC in 2016: 172) Age in years, (mean SD) Male, *n* (%): FC: 15% vs. TC: 12%)
**Interventions**	Intervention: Blended learning/flipped class: FC Control/comparator: Face‐to‐ face class/traditional class (TC)
**Outcomes**	Academic performance Course evaluation
**Notes**	Setting: Ostfold University College in Norway Ethics approval: ‘approval by the Norwegian Social Science Data Services (NSD) ethical guidelines for experimental studies’. Funding: Østfold University College's Strategic Found for digital development projects. Study period: 1st semester 2016 and 1st semester 2017

Risk of bias table

**Table:** 

Bias	Authors' judgement	Support for judgement
Random sequence generation (selection bias)	High risk	Seems no randomisation Quote: ‘was used as the study object in a quasi‐experimental design’
Allocation concealment (selection bias)	High risk	Different year.
Blinding of participants and personnel (performance bias)	High risk	Seems no blinded Quote: ‘the recordings were provided by the bioscientist who gave most of the lectures and was well known to the students’
Blinding of outcome assessment (detection bias)	High risk	Seems no blinded Quote: ‘the recordings were provided by the bioscientist who gave most of the lectures and was well known to the students’
Incomplete outcome data (attrition bias)	Low risk	All analysed
Selective reporting (reporting bias)	Unclear risk	Cannot be determined (no protocol)
Other bias	Low risk	None
Confounding?/Free of contamination?	Unclear risk	
Baseline characteristics similar?	Unclear risk	
Baseline outcomes similar?	Unclear risk	
Intervention independent	Unclear risk	
Blinding	Unclear risk	
incomplete outcome data	Unclear risk	

Harrington 2015

**Table:** 

**Methods**	RCT
**Participants**	Semester 2, undergraduate nursing students *N* = 82 (n = 41 per each group) Male, *n* (%): Not mentioned Age in years: Not mentioned inclusion/exclusion criteria: Not described
**Interventions**	**Intervention**: Flipped classroom **Control/comparator**; traditional class
**Outcomes**	3 exams (24 quizzes, written paper)
**Notes**	Setting: baccalaureate nursing program of a public university, USA Ethical approval: Obtained exemption. Funding: Not mentioned Study period: 01/2013–04/2013

Risk of bias table

**Table:** 

Bias	Authors' judgement	Support for judgement
Random sequence generation (selection bias)	High risk	Quote: ‘convenient randomisation’. p. 179
Allocation concealment (selection bias)	High risk	Quote: ‘randomly assigned to the traditional class or to the flipped classroom’. p. 179
Blinding of participants and personnel (performance bias)	High risk	Quote: ‘Four faculty members taught course content based on their expertise in both the traditional and the flipped classroom’. p. 179
Blinding of outcome assessment (detection bias)	High risk	Quote: ‘Examinations were given to both groups at the same time and in the same classroom’. p. 179
Incomplete outcome data (attrition bias)	Unclear risk	Not stated
Selective reporting (reporting bias)	Unclear risk	No protocol available
Other bias	Low risk	None
Confounding?/Free of contamination?	Unclear risk	
Baseline characteristics similar?	Unclear risk	
Baseline outcomes similar?	Unclear risk	
Intervention independent	Unclear risk	
Blinding	Unclear risk	
incomplete outcome data	Unclear risk	

Heitz 2015

**Table:** 

Methods	RCT
**Participants**	*N* = 60 recruited N = 56 analysed Late 3rd year (*n* = 35)/4th‐year (*n* = 21) medical students in EM rotation/EM elective *N* = 60 recruited *N* = 56 analysed Male, *n* (%): not mentioned Age in years: not mentioned Inclusion criteria: Late 3rd or 4th year medical students enroled in the required EM rotation or the EM elective at either site. ii. Participants underwent informed consent at the beginning of the rotation that participation in the study was inconsequential to their final grade on the rotation Exclusion criteria: Not described
**Interventions**	**Intervention**: Flipped class (FC) **Control/comparator**: Standard class; 4 week rotation (SC)
**Outcomes**	Primary: Scores archived for a flipped clerkship vs standard learning on 10 peer‐reviewed MCQs
**Notes**	Setting: Two academic sites (Virginia Tech Carilion School of Medicine & University of Maryland School of Medicine), USA Ethical approval: Obtained. Funding: Not mentioned Study period: 01/07/to 30/06/2014

Risk of bias table

**Table:** 

Bias	Authors' judgement	Support for judgement
Random sequence generation (selection bias)	High risk	Quote: ‘designated a study number that assigned them’. p. 852
Allocation concealment (selection bias)	High risk	Quote: ‘…assigned them to a combination of two chief complaints commonly’ Quote: ‘participants were assigned to one of the six combinations of chief complaints’. p. 852
Blinding of participants and personnel (performance bias)	Unclear risk	Not stated
Blinding of outcome assessment (detection bias)	Unclear risk	Quote: ‘All participants took the same examination, although the order of questions was altered by the testing software to minimize chances of unethical behavior’. p. 851
Incomplete outcome data (attrition bias)	Low risk	Less than 10% withdrawn (4/60 students)
Selective reporting (reporting bias)	Low risk	Protocol was presented in the paper
Other bias	Unclear risk	Quote: ‘The FC on the examination was not considered towards their final grade on the rotation’. p. 852
Confounding?/Free of contamination?	Unclear risk	
Baseline characteristics similar?	Unclear risk	
Baseline outcomes similar?	Unclear risk	
Intervention independent	Unclear risk	
Blinding	Unclear risk	
incomplete outcome data	Unclear risk	

Herrero 2020

**Table:** 

**Methods**	Quasi‐experimental
**Participants**	3rd year medical students in 2 consecutive year (2017–2018 and 2018–2019) in pathophysiology course *N* = 430 in 2 consecutive year (FC, 201 vs. TC 229) Male, *n* (%): FC, 78 (38.8); TC, 73(31.9) Age in years, mean (±SD): FC, 20(74.6%) and >20 (25.4%) TC 20(83%) and >20 (17%) Inclusion criteria: Two different cohort included.
**Interventions**	**Intervention**: Flipped class (FC) in 2018 **Control/comparator:** Traditional class (TC) in 2017
**Outcomes**	Student performance (exam scores of 100 MCQ)
**Notes**	Setting: the Universidad de Navarra (Pamplona, Spain) Ethical approval: obtained (project 2018–112) Funding: Not mentioned Study period: 2017‐2018

Risk of bias table

**Table:** 

Bias	Authors' judgement	Support for judgement
Random sequence generation (selection bias)	High risk	Quote: ‘absence of randomisation’. p. 374
Allocation concealment (selection bias)	High risk	Not stated(seems not conceled)
Blinding of participants and personnel (performance bias)	Unclear risk	Not stated
Blinding of outcome assessment (detection bias)	Low risk	Quote: ‘Data from the students were recorded in a coded database, without personal information’. p. 371
Incomplete outcome data (attrition bias)	High risk	Low response rate (10%), nonresponse bias
Selective reporting (reporting bias)	Unclear risk	Not known
Other bias	Unclear risk	Quote ‘Lack of an evaluation of systematic biases’ (p. 374)
Confounding?/Free of contamination?	Unclear risk	
Baseline characteristics similar?	Unclear risk	
Baseline outcomes similar?	Unclear risk	
Intervention independent	Unclear risk	
Blinding	Unclear risk	
incomplete outcome data	Unclear risk	

Hu 2019

**Table:** 

**Methods**	Intervention study with two groups (Quasi‐experimental design)
**Participants**	4th year medical students in PBL of endocrinology (hyperthyroidism) course *N* = 74 (FC, 37: TC vs. TC, 37) Male, *n* (%): FC, 19(51.4): TC, 18 (48.6) Age in years, mean ± SD): FC, 22.4 ± 0.9; TC, 2.1 ± 1.0 I**nclusion/exclusion criteria:** Not described
**Interventions**	**Intervention**: Flipped classroom with problem‐based learning (FCPBL) **Control/comparator**: Traditional lecture‐based class (TC)
**Outcomes**	Mean scores of pre–post‐quiz; Students' perspectives, self‐perceived competence, satisfaction (**≥**4 points was defined as satisfactory)
**Notes**	Setting: Internship at the First Affiliated Hospital of Bengbu Medical College, China Ethics approval: Obtained. Funding: Quality Project for Undergraduate Teaching, Bengbu Medical College. (Grant # 2017jyxm62). Study period: Not mentioned

Risk of bias table

**Table:** 

Bias	Authors' judgement	Support for judgement
Random sequence generation (selection bias)	High risk	Randomly allocated into either group; no further details provided
Allocation concealment (selection bias)	High risk	Randomly allocated
Blinding of participants and personnel (performance bias)	Low risk	Quote: ‘All students were unaware of their group assignments before the internship’. p. 2
Blinding of outcome assessment (detection bias)	Low risk	Quote: ‘As numbers were used in the quizzes and surveys instead of real names’. p. 3
Incomplete outcome data (attrition bias)	Low risk	No withdrawal
Selective reporting (reporting bias)	Unclear risk	A protocol is not available
Other bias	Low risk	None
Confounding?/Free of contamination?	Unclear risk	
Baseline characteristics similar?	Unclear risk	
Baseline outcomes similar?	Unclear risk	
Intervention independent	Unclear risk	
Blinding	Unclear risk	
incomplete outcome data	Unclear risk	

Huang 2020

**Table:** 

**Methods**	Quasi‐experimental design
**Participants**	Medical technology students *N* = 62 (FC,38 vs. TC, 24) Male, *n* (%): FC: 5(13.15); TC: 4(16.7) Age in years, (mean ± SD): FC: (20.3 ± 1.5); TC (20.4 ± 1.3)
**Interventions**	**Intervention**: Flipped class (FC) **Control/comparison**: Traditional class (TC)
**Outcomes**	Fresno test scores; Student satisfaction (self‐made questions with open‐ended questions)
**Notes**	Setting: Kaohsiung Chang Gung Memorial Hospital, Taiwan Ethics approval: Obtained from Kaohsiung Chang Gung Memorial Hospital, Taiwan Funding: Kaohsiung Chang Gung Memorial Hospital, Taiwan and Ministry of Science and Technology of Taiwan Study period: Not mentioned

Risk of bias table

**Table:** 

Bias	Authors' judgement	Support for judgement
Random sequence generation (selection bias)	High risk	Quote: participants could not be randomly assigned
Allocation concealment (selection bias)	High risk	Not stated (seems not concealed)
Blinding of participants and personnel (performance bias)	Unclear risk	Quote ‘As teachers and students were participating a research, there might be Pygmalion effect in which student's performance is affected by teacher's expectation’. p. 8
Blinding of outcome assessment (detection bias)	Unclear risk	Not stated
Incomplete outcome data (attrition bias)	Low risk	No withdrawal
Selective reporting (reporting bias)	Unclear risk	No protocol available
Other bias	Unclear risk	None
Confounding?/Free of contamination?	Unclear risk	
Baseline characteristics similar?	Unclear risk	
Baseline outcomes similar?	Unclear risk	
Intervention independent	Unclear risk	
Blinding	Unclear risk	
incomplete outcome data	Unclear risk	

Isherwood 2019

**Table:** 

**Methods**	RCT
**Participants**	5th year undergraduate dental students in December 2017–March 2018 *N* = 61, (FC 31 vs. CL 30) Inclusion criteria: 5th yr LUDH undergraduate Dental Students hospital scheduled to undertake teaching on Orthodontic emergencies volunteering Exclusion criteria: students repeating their 5th year of the BDS
**Interventions**	**Intervention**: Flipped class (FC) **Control/comparator:** Conventional lecture (CL)
**Outcomes**	Formative assessment (20 OBA) Mean exam result Perceptions of flipped classroom (Focus group)
**Notes**	Setting: Liverpool University, UK Study period: 07/09/2017–30/09/2017

Risk of bias table

**Table:** 

Bias	Authors' judgement	Support for judgement
Random sequence generation (selection bias)	Low risk	Computer generated random allocation
Allocation concealment (selection bias)	Low risk	Concealed from the main investigator
Blinding of participants and personnel (performance bias)	Low risk	Quote: ‘unseen by the participants’ p. 60, Chapter 6.9
Blinding of outcome assessment (detection bias)	High risk	Quote: Neither the main researcher nor the participants were blinded. p. 62, Chapter 6.10
Incomplete outcome data (attrition bias)	Low risk	No withdrawal
Selective reporting (reporting bias)	Unclear risk	Protocol not available
Other bias	Unclear risk	Not known
Confounding?/Free of contamination?	Unclear risk	
Baseline characteristics similar?	Unclear risk	
Baseline outcomes similar?	Unclear risk	
Intervention independent	Unclear risk	
Blinding	Unclear risk	
incomplete outcome data	Unclear risk	

Kuhl 2017

**Table:** 

**Methods**	RCT
**Participants**	2nd semester medical students *N* = 341 (IC, 42 vs. TC, 299) Male, *n* (%): not mentioned Age in years: Not mentioned Inclusion/exclusion criteria: Not described
**Interventions**	**Intervention**: Inverted class (IC). Two IC intervention groups **Control/comparator**: Traditional class (TC): 14 groups
**Outcomes**	Motivation, satisfaction, acceptance of the teaching methods biochemistry EOS score
**Notes**	Setting: Medical Faculty in Ulm University, Germany Ethical approval: Obtained Funding: ‘Sonderlinie Medizin’ of the State of Baden‐Württemberg, Germany. Study period: Summer semester 2016

Risk of bias table

**Table:** 

Bias	Authors' judgement	Support for judgement
Random sequence generation (selection bias)	Unclear risk	Quote: ‘14 groups with 299 students were assigned at random to the traditional control group and 2 groups with 42 students to the IC intervention group’. p. 3
Allocation concealment (selection bias)	High risk	Quote: ‘assigned to groups by the office of student affairs of the medical Faculty without any influence from the lecturers’. p. 3
Blinding of participants and personnel (performance bias)	High risk	Quote: ‘The traditional student group was taught by a team of mainly experienced lecturers who had held the seminar in this form for several years (12 out of the 14 control groups)’. p. 11
Blinding of outcome assessment (detection bias)	High risk	Quote: ‘The FC groups received these in paper form during the on‐site phase II, the traditional groups online in an e‐mail sent to the students that contained a link to the online survey via the evaluation platform EvaSys’. p. 5
Incomplete outcome data (attrition bias)	Unclear risk	No protocol available
Selective reporting (reporting bias)	Unclear risk	Quote: ‘the use of the knowledge test for only the IC group’. p. 11
Other bias	Unclear risk	Quote: ‘the lecturer for the IC intervention group displayed a high level of motivation’. p. 11
Confounding?/Free of contamination?	Unclear risk	
Baseline characteristics similar?	Unclear risk	
Baseline outcomes similar?	Unclear risk	
Intervention independent	Unclear risk	
Blinding	Unclear risk	
incomplete outcome data	Unclear risk	

Lin 2017

**Table:** 

**Methods**	RCT two‐group parallel design
**Participants**	International students enroled in MBBS *N* = 44 (FC: 22 vs. TC: 22), Male, *n* (%): FC: 9(40.9); TC: 10(45.4) Age in years, (mean ± SD): FC (24.2 ± 2.2); TL (23.5 ± 1.1)
**Interventions**	**Intervention**: Flipped classroom (FC) **Control/comparison**: 1. Traditional lecture‐based curriculum & FC (TLFC) in glaucoma classroom (*n* = 22) 2. TLFC in ocular trauma classroom (n = 22)
**Outcomes**	Pre‐test scores Final exam scores Feedback questionnaires (students, *N* = 44, teachers, N = 10) Students' and teachers' attitudes towards FC
**Notes**	Setting: Zhongshan Ophthalmic Center (ZOC) of Sun Yat‐sen University, China Ethical approval: Obtained Funding: Multiple sources, Sun Yat‐sen University (2016‐150‐Ying Lin and 2016‐3‐Bingqian Liu), the National Natural Science Foundation of China (Grant # 81500709, 81570862, 81371019, 81670872), Medical Scientific Research Foundation of Guangdong Province (Grant #. B2012126, A2016460), the Project of Fundamental Research (Grant # B2012126, A2016460) Study period: Spring of 2016

Risk of bias table

**Table:** 

Bias	Authors' judgement	Support for judgement
Random sequence generation (selection bias)	High risk	Random assignment, no further details
Allocation concealment (selection bias)	High risk	Randomly divided into two groups
Blinding of participants and personnel (performance bias)	Low risk	Quote: ‘All the subjects were not aware of the differences in the course format before the enrolment’. p. 3
Blinding of outcome assessment (detection bias)	Unclear risk	Quote: ‘the instructor summarized the whole class, and reviewed all questions from the discussion’. p. 4
Incomplete outcome data (attrition bias)	Low risk	No withdrawal
Selective reporting (reporting bias)	Low risk	Procedures revealed in the paper
Other bias	Unclear risk	Students from diverse background
Confounding?/Free of contamination?	Unclear risk	
Baseline characteristics similar?	Unclear risk	
Baseline outcomes similar?	Unclear risk	
Intervention independent	Unclear risk	
Blinding	Unclear risk	
incomplete outcome data	Unclear risk	

Lucchetti 2018

**Table:** 

**Methods**	Intervention study, with a non‐randomised control group (Quasi experimental)
**Participants**	3rd year medicine (5th semester) in geriatrics and gerontology *N* = 243 (77 CG vs. TL, 83 vs FC 83) Male (%): CG (38.7), TL (38.3), FC (45.8)
**Interventions**	**Intervention**: Flipped class (FC) ‐ interactive activities (team‐based learning, discussion of clinical cases, group or paired work, jigsaw and application of the content in class) **Control/comparator**: 1. Traditional, lecture‐based class (TL)**—**provided complementary bibliography online for references, non‐mandatory online homework 2. Conventional group (CG): No intervention
**Outcomes**	Pre‐and post‐intervention assessment Comparison between CG versus TR/FL 1. Attitudes (UCLA‐GAS); 2. Knowledge (Cognitive**—**Basic geriatric knowledge); 3. Attitudes (Palmore Positivism); 4. Attitudes (Palmore total); 5. Attitudes (Maxwell–Sullivan); 6. Empathy (Maxwell–Sullivan); 7. Standardised Patient
**Notes**	Setting: Federal University of Juiz de Fora's (UFJF), School of Medicine, Brazil Ethical approval: obtained Funding: Brazilian National Council for Scientific & Technological Development (Grant # 425074/2016‐1). Study period: 07/2014 to 07/2016

Risk of bias table

**Table:** 

Bias	Authors' judgement	Support for judgement
Random sequence generation (selection bias)	High risk	No randomisation
Allocation concealment (selection bias)	High risk	Quasi design
Blinding of participants and personnel (performance bias)	High risk	Quote: ‘The amphitheater lectures of both groups were given by the same lecturer, and the practices were assessed by the same eight trainers’. p. 4
Blinding of outcome assessment (detection bias)	High risk	Quote: ‘conducted by the same TR professors’
Incomplete outcome data (attrition bias)	Low risk	Only 7 absentee in the CG (9%)
Selective reporting (reporting bias)	Unclear risk	No protocol available
Other bias	Unclear risk	Share similar complementary bibliography and same practical classes
Confounding?/Free of contamination?	Unclear risk	
Baseline characteristics similar?	Unclear risk	
Baseline outcomes similar?	Unclear risk	
Intervention independent	Unclear risk	
Blinding	Unclear risk	
incomplete outcome data	Unclear risk	

Missildine 2013

**Table:** 

**Methods**	Quasi‐experimental
**Participants**	Baccalaureate nursing students *N* = 589 over 3 semesters (LCI, 53, LO, 53, LLC,53) Male (%): 19% Age in years, (mean ± SD): 24.32 Inclusion/exclusion criteria; not described
**Interventions**	**Intervention**: Lecture capture plus innovation (LCI) **Control/comparison**: Lecture only (LO) ‐ Lecture plus lecture capture (LLC)
**Outcomes**	course exam scores; satisfaction (4‐point Likert scale, Faculty‐developed questionnaire)
**Notes**	Setting: College of Nursing, University of Texas, USA Ethical approval: Obtained Funding: The University of Texas at Tyler through the J. Burns Brown Fellowship award. Study duration **—** three semesters LLC in spring 2010; LO & LCI in fall 2010;

Risk of bias table

**Table:** 

Bias	Authors' judgement	Support for judgement
Random sequence generation (selection bias)	High risk	Convenience sampling; sample size calculated
Allocation concealment (selection bias)	High risk	Convenience sampling
Blinding of participants and personnel (performance bias)	Low risk	Quote: ‘Simulation case studies, games, and other exercises were implemented independently by faculty on each campus’. p.598
Blinding of outcome assessment (detection bias)	High risk	Quote: ‘Comparable examination items on test metrics were used from semester to semester to ensure consistency’. p. 598
Incomplete outcome data (attrition bias)	High risk	Quote: ‘satisfaction survey completed by 75.55% response rate’. p. 598
Selective reporting (reporting bias)	Unclear risk	No protocol available
Other bias	Low risk	None
Confounding?/Free of contamination?	Unclear risk	
Baseline characteristics similar?	Unclear risk	
Baseline outcomes similar?	Unclear risk	
Intervention independent	Unclear risk	
Blinding	Unclear risk	
incomplete outcome data	Unclear risk	

Morton 2017

**Table:** 

Methods	Cohort study
**Participants**	1st year medical students *N* = 203 (FC, 102 vs. LC, 101) Male, *n* (%): FC, 53 (52), LC, 48 (47) Age in years: not mentioned Inclusion criteria: 1st year medical students in 2013 (*n* = 5101) and 2014 (*n* = 5102) who were enroled in Foundations of Medicine (FOM).
**Interventions**	**Intervention**: Flipped Classroom in 2014 (FC) **Control/Comparator**: Lecture classroom in 2013 (LC)
**Outcomes**	Performance of final exam (150 exam items) assessing each Bloom's level of cognition
**Notes**	Setting: University of Utah School of Medicine, USA Ethical approval: Obtained Funding: Not mention Study period: 2013–2014

Risk of bias table

**Table:** 

Bias	Authors' judgement	Support for judgement
Random sequence generation (selection bias)	Unclear risk	
Allocation concealment (selection bias)	Unclear risk	
Blinding of participants and personnel (performance bias)	Unclear risk	
Blinding of outcome assessment (detection bias)	Unclear risk	
Incomplete outcome data (attrition bias)	Unclear risk	
Selective reporting (reporting bias)	Unclear risk	
Other bias	Unclear risk	
Confounding?/Free of contamination?	High risk	Quote: ‘Between subjects design’. p. 171
Baseline characteristics similar?	High risk	Quote: ‘it is unknown if the students were both classes were truly different academically based on the overall examination score since we were unable to analyse pre‐matriculation performance by class’. p. 174
Baseline outcomes similar?	High risk	Different exam items
Intervention independent	Unclear risk	Not stated
Blinding	Unclear risk	Not stated
incomplete outcome data	Unclear risk	Not stated

O'Connor 2016

**Table:** 

**Methods**	Prospective cohort study Multi‐institutional study of 3 Universities
**Participants**	3rd/4th year medical students in a 4‐week radiology clerkship or radiology elective *N* = 175 Male, *n* (%): Not mentioned Age in years: Not mentioned Exclusion criteria: Not described
**Interventions**	**Intervention**: Flipped class learning (FC) **Control/Comparator**: Traditional class didactic instruction (TC)
**Outcomes**	pretest‐posttest on general diagnostic imaging knowledge
**Notes**	Setting: 1. Temple University School of Medicine, Philadelphia, USA 2. Geisel School of Medicine at Dartmouth, USA 3. West Virginia University School of Medicine, Morgan Town, USA Ethics approval: Obtained. Funding: Not mentioned Study period: 01/2014 to 04/2015

Risk of bias table

**Table:** 

Bias	Authors' judgement	Support for judgement
Random sequence generation (selection bias)	Unclear risk	
Allocation concealment (selection bias)	Unclear risk	
Blinding of participants and personnel (performance bias)	Unclear risk	
Blinding of outcome assessment (detection bias)	Unclear risk	
Incomplete outcome data (attrition bias)	Unclear risk	
Selective reporting (reporting bias)	Unclear risk	
Other bias	Unclear risk	
Confounding?/Free of contamination?	High risk	Quote: ‘The survey assessment of task value and achievement emotions may have been confounded by effects of concurrent non‐neuroimaging teaching sessions’. ‘Variations in class size, ranging from 3 to 12 students per block, could have had an effect on both instruction and learning’. p. 818
Baseline characteristics similar?	High risk	4 different instructors and students from 3 institutions Quote ‘inability to control for differences in stylistic approach by instructors’. p. 818
Baseline outcomes similar?	High risk	Differences in instructional time Quote ‘It is possible that this difference could have affected student performance, task value, and achievement emotions’. p. 818
Intervention independent	Unclear risk	Quote: ‘Student assignment to flipped learning (intervention group) or traditional didactic lectures (control group) alternated with each block of the clerkship’. p. 813
Blinding	Low risk	Quote ‘Instructors were blinded as to which students enrolled in the study’. p. 813
incomplete outcome data	Unclear risk	Not stated

Park 2018

**Table:** 

**Methods**	Quasi‐experimental design
**Participants**	Junior students from a nursing science major in 2015 *N* = 81 (FC, 81 vs. TC, 81; 1st half and 2nd half of the study) Age in years (mean ± SD): 22.1(0.89) Male, *n* (%): 9 (11.1) Inclusion criteria: Voluntary participants
**Interventions**	**Intervention**: Flipped class (FC) **Control/Comparator**: Traditional class (TC)
**Outcomes**	Critical thinking (mean score difference) Academic achievement (mean score difference)
**Notes**	Setting: Daegu University, Daegu, South Korea. Ethics approval: Obtained Funding: Daegu University, Daegu, South Korea (No. 20160195). Study period: 03/2015–06/2015

Risk of bias table

**Table:** 

Bias	Authors' judgement	Support for judgement
Random sequence generation (selection bias)	High risk	convenience sampling
Allocation concealment (selection bias)	High risk	Each participant involved in both methods (1st half and 2nd half of the study)
Blinding of participants and personnel (performance bias)	High risk	Quote: ‘each participant was tested multiple times to see’
Blinding of outcome assessment (detection bias)	Unclear risk	The same participants involved in both groups.
Incomplete outcome data (attrition bias)	Low risk	No withdrawal
Selective reporting (reporting bias)	Low risk	Procedure described
Other bias	Low risk	None
Confounding?/Free of contamination?	Unclear risk	
Baseline characteristics similar?	Unclear risk	
Baseline outcomes similar?	Unclear risk	
Intervention independent	Unclear risk	
Blinding	Unclear risk	
incomplete outcome data	Unclear risk	

Ren 2020

**Table:** 

Methods	RCT
**Participants**	Medical students enroled in histology and biochemistry experiments *N* = 180 (FC: 87 vs. TC: 93) Age in years (mean ± SD) Male, *n* (%): Not mentioned
**Interventions**	Intervention: Flipped class (FC) Control/Comparator: Traditional class (TC)
**Outcomes**	Academic performance (test scores) Students satisfaction
**Notes**	Setting: Dalian Medical University, China Ethics approval: approved by the Dalian Medical University Funding: Grant No. 14YJA880106 from the General Project of the Humanities and Social Sciences Research Fund of the Ministry of Education; Grant No. UPRP20160383 from the research project on the undergraduate teaching reform of general higher education in Liaoning Province; Grant No. JG17DB140 from Liaoning Province Education Science ‘13th Five‐Year Plan’ Project; Grant No. 2016B‐YJS019, 2016B‐JS013, 2016B‐JC014 from Medical Education Research Topics 2016 of Medical Education Branch of Chinese Medical Association. Study period: Not mentioned

Risk of bias table

**Table:** 

Bias	Authors' judgement	Support for judgement
Random sequence generation (selection bias)	Unclear risk	Need more information Quote: ‘Some participants were randomly located into common (*n* = 93) and flipped (*n* = 87) group’
Allocation concealment (selection bias)	Unclear risk	Need more information
Blinding of participants and personnel (performance bias)	Low risk	All students were unaware of their group assignments before class
Blinding of outcome assessment (detection bias)	Low risk	All students were unaware of their group assignments before class.
Incomplete outcome data (attrition bias)	Low risk	All analysed
Selective reporting (reporting bias)	Unclear risk	Cannot be determined (No protocol)
Other bias	Low risk	None
Confounding?/Free of contamination?	Unclear risk	
Baseline characteristics similar?	Unclear risk	
Baseline outcomes similar?	Unclear risk	
Intervention independent	Unclear risk	
Blinding	Unclear risk	
incomplete outcome data	Unclear risk	

Rui 2017

**Table:** 

Methods	RCT
**Participants**	Junior‐year medical undergraduates majoring in clinical medicine (2015–2016) *N* = 181 (FC, 90 vs. LBL, 91) Male, *n* (%): FC, 41(45.6); LBL, 50 (54.9) Age in years, mean ± SD: FC (0.84 ± 0.67), LBL (20.90 ± 0.58) Inclusion criteria: those who agreed and signed the consent form
**Interventions**	**Intervention** = Flipped classroom (FC) **Control/comparison** = Lecture‐based Learning (LBL)
**Outcomes**	Test scores 1 week after intervention Self‐administered questionnaire Students ‘attitudes towards FC Comparison of the Investment in studies
**Notes**	Setting: Sichuan University, Chengdu, China Ethics approval: Obtained Funding: Daegu University, Daegu, South Korea (# 20160195). Study period: 2015–2016

Risk of bias table

**Table:** 

Bias	Authors' judgement	Support for judgement
Random sequence generation (selection bias)	Low risk	Computer‐based random digital method
Allocation concealment (selection bias)	Low risk	Computer‐based
Blinding of participants and personnel (performance bias)	Unclear risk	Not stated
Blinding of outcome assessment (detection bias)	Unclear risk	Not stated
Incomplete outcome data (attrition bias)	Low risk	No withdrawal
Selective reporting (reporting bias)	Low risk	Procedure described
Other bias	Low risk	None
Confounding?/Free of contamination?	Unclear risk	
Baseline characteristics similar?	Unclear risk	
Baseline outcomes similar?	Unclear risk	
Intervention independent	Unclear risk	
Blinding	Unclear risk	
incomplete outcome data	Unclear risk	

Sajid 2020

**Table:** 

**Methods**	Quasi‐experimental study
**Participants**	second‐year MBBS students during the Neuroscience Block *N* = 215, FC: 136 vs. TC: 79 Analysed: *N* = 193, FC 128 vs TC 65 Male, n (%): FC, 0(0%); TC, 79(100%) Age in years (mean ± SD): Not mentioned
**Interventions**	Intervention: Flipped class (FC) Control/comparison: lecture‐based classroom group (i.e., traditional class): TC
**Outcomes**	Academic performance (pre–post test with MCQs) Students' feedback
**Notes**	Setting: College of Medicine, Alfaisal University, Riyadh, Saudi Arabia Ethics approval: Institutional Review Board (IRB) approval (vide IRB‐20004) Funding: Study period:

Risk of bias table

**Table:** 

Bias	Authors' judgement	Support for judgement
Random sequence generation (selection bias)	High risk	Quasi‐experimental design (i.e no randomisation)
Allocation concealment (selection bias)	High risk	All females in FC, all males in TC
Blinding of participants and personnel (performance bias)	High risk	Students were aware of their group assignments
Blinding of outcome assessment (detection bias)	High risk	seems no blinding
Incomplete outcome data (attrition bias)	Low risk	81% analysed
Selective reporting (reporting bias)	Unclear risk	Can not be determined (No protocol
Other bias	Low risk	None
Confounding?/Free of contamination?	Unclear risk	
Baseline characteristics similar?	Unclear risk	
Baseline outcomes similar?	Unclear risk	
Intervention independent	Unclear risk	
Blinding	Unclear risk	
incomplete outcome data	Unclear risk	

Sinclair‐Bennett 2019

**Table:** 

**Methods**	Quasi‐experimental study, convenience sampling
**Participants**	Associate degree nursing students (*N* = 93, FC 42 vs. TC 51) *N* = 93 (FC: 42 vs. TC: 51) Male, *n* (%): FC, 7 (16.7) vs. TC, 10 (19.6) Age under 25 years, *n* (%): 35 (37.65%) Inclusion criteria: 1. students currently enroled in a medical surgical course 2. completed a fundamentals of nursing course Exclusion criteria: 1. novice students (i.e., 1st semester nursing students), 2. last semester nursing students 3. students enrolled in maternal newborn, pediatrics or mental health nursing courses
**Interventions**	Intervention: Flipped class (FC) Control: Traditional lecture class (TC)
**Outcomes**	clinical reasoning scores (pre‐ and posttest)
**Notes**	Setting: Capella University, Minnesota, USA Ethics approval: Obtained Study period: 3 separate times during the 2018‐2019 school year. Power analysis for sample size calculations done

Risk of bias table

**Table:** 

Bias	Authors' judgement	Support for judgement
Random sequence generation (selection bias)	High risk	Non‐ random
Allocation concealment (selection bias)	High risk	Students from two different campus assigned to two different methods
Blinding of participants and personnel (performance bias)	Low risk	Used a log in and password which was not correlated with the student's name or personal identification such as gender or race
Blinding of outcome assessment (detection bias)	Low risk	Used a log in and password
Incomplete outcome data (attrition bias)	Low risk	No withdrawal
Selective reporting (reporting bias)	Low risk	All outcomes were addressed
Other bias	Unclear risk	1. Mean years of employment in health care; 4.45 in FC; 2.5 years in TC 2. The control group had 8 h more in‐class instruction and 16 h more in the clinical environment
Confounding?/Free of contamination?	Unclear risk	
Baseline characteristics similar?	Unclear risk	
Baseline outcomes similar?	Unclear risk	
Intervention independent	Unclear risk	
Blinding	Unclear risk	
incomplete outcome data	Unclear risk	

Stewart 2013

**Table:** 

**Methods**	retrospective cohort study
**Participants**	3rd year pharmacotherapy course pod casting group in 2010 *N* = 136 (AL,71 vs. DC, 65) Male, *n* (%): Not mentioned Age in years: Not mentioned Inclusion/exclusion criteria: Not described
**Interventions**	**Intervention**: Podcasting and active learning in 2010 (AL) **Control/comparator**: Didactic class in 2009 (DC)
**Outcomes**	End‐of‐course exam scores, MCQs
**Notes**	Setting: East Tennessee State University, USA Ethics approval: Obtained Funding: Not mentioned Study period: Fall semesters of 2009 (control) and 2010 (pod casts).

Risk of bias table

**Table:** 

Bias	Authors' judgement	Support for judgement
Random sequence generation (selection bias)	Unclear risk	
Allocation concealment (selection bias)	Unclear risk	
Blinding of participants and personnel (performance bias)	Unclear risk	
Blinding of outcome assessment (detection bias)	Unclear risk	
Incomplete outcome data (attrition bias)	Unclear risk	
Selective reporting (reporting bias)	Unclear risk	
Other bias	Unclear risk	
Confounding?/Free of contamination?	High risk	Students having different GPA
Baseline characteristics similar?	High risk	Quote: ‘major limitation in this interpretation is that students were not held responsible for completing the assignment, thus it is likely that students in the lower 50% of their class chose not to complete the out‐of‐class material as assigned’. p. 577
Baseline outcomes similar?	Low risk	Quote: ‘same multiple‐choice questions in different cohort’. p. 575 End of course exam (same quality of exam)
Intervention independent	Low risk	different years; Quote: ‘The exam questions……… used were very similar between multiple cohorts(including the two cohorts evaluated over 4 yr period, making the questions themselves less likely to be confounders’. p. 575
Blinding	Unclear risk	Insufficient information
incomplete outcome data	Low risk	None

Street 2015

**Table:** 

**Methods**	Quasi‐experimental design
**Participants**	5th year medical students, preclinical physiology course; FC in 2013–2014 cohort; TC in 2012–2013 cohort *N*: 360 (FC, 180 vs. TC 180) Male, %: FC, 47.2%; TC, 55% Age in years: not mentioned Inclusion/exclusion criteria: Not described.
**Interventions**	**Intervention**: Flipped classroom in 2013–2014 cohort (FC) **Control/comparator**: Traditional class in 2012‐2013 cohort (TC)
**Outcomes**	Performance on examination Student satisfaction (course evaluation, survey) Flipped classroom survey (143/180)
**Notes**	Setting: University of North Carolina School of Medicine, USA Ethical approval: Obtained (#14‐1218). Funding: Not mentioned Study period: Group 1, TC: 2012–2013 cohort Group 2, FC: 2013–2014 cohort

Risk of bias table

**Table:** 

Bias	Authors' judgement	Support for judgement
Random sequence generation (selection bias)	High risk	Not mentioned
Allocation concealment (selection bias)	High risk	Different cohorts in different academic years
Blinding of participants and personnel (performance bias)	Unclear risk	Two different cohorts
Blinding of outcome assessment (detection bias)	Unclear risk	Two different cohorts
Incomplete outcome data (attrition bias)	Low risk	No withdrawal
Selective reporting (reporting bias)	Low risk	Same outcomes measured; both cohorts consisted of 26 common items.
Other bias	Low risk	None
Confounding?/Free of contamination?	Unclear risk	
Baseline characteristics similar?	Unclear risk	
Baseline outcomes similar?	Unclear risk	
Intervention independent	Unclear risk	
Blinding	Unclear risk	
incomplete outcome data	Unclear risk	

Suda 2014

**Table:** 

**Methods**	Quasi‐experimental design
**Participants**	3rd year pharmacy students in drug information and literature evaluation course; FC in 2013, TC in 2012 N = 319 (FC, 143 vs. TC 176) Male, *n* (%): Not mentioned. Age in years: Not mentioned
**Interventions**	**Intervention**: Blended learning (Flipped class): FC **Control/comparator**: Traditional class: TC
**Outcomes**	Final exam (MCQs) Overall course grades Course evaluations Survey (respondents *N* = 140)
**Notes**	Setting: College of Pharmacy in the University of Tennessee, USA Ethic approval: Exempted (p. 368) Funding: The Scholarship of Teaching and Learning Seed Grant Program by the University of Tennessee, College of Pharmacy Study period: Fall, semester of 2011 Definitions: Blended learning = a course composed of online lectures and in‐class active learning sessions. Online lectures = Lectures that were viewed using Media‐sites technology

Risk of bias table

**Table:** 

Bias	Authors' judgement	Support for judgement
Random sequence generation (selection bias)	High risk	Not mentioned
Allocation concealment (selection bias)	High risk	Quote: ‘Based on team readiness assurance tests (TRATs)’. p. 368
Blinding of participants and personnel (performance bias)	High risk	Quote: ‘Both course offerings were taught using synchronous distance learning technology’. p.368
Blinding of outcome assessment (detection bias)	Low risk	Quote: ‘Students were asked to complete an anonymous, self‐administered online survey at the conclusion of the semester’. p. 368
Incomplete outcome data (attrition bias)	Low risk	No withdrawal
Selective reporting (reporting bias)	Unclear risk	Protocol not available
Other bias	Low risk	None
Confounding?/free of contamination?	Unclear risk	
Baseline characteristics similar?	Unclear risk	
Baseline outcomes similar?	Unclear risk	
Intervention independent	Unclear risk	
Blinding	Unclear risk	
incomplete outcome data	Unclear risk	

Tang 2017

**Table:** 

**Methods**	Quasi‐experimental design
**Participants**	4th year medical students in an ophthalmology clerkship *N* = 95, (FC, 48 vs. TC, 47) Male, *n* %: FC, 25 (52); TC, 23 (48.9) Age in years (mean ± SD): FC (2.3 ± 0.6); TC (22.6 ± 0.4) Inclusion/exclusion criteria: Not described
**Interventions**	**Intervention:** Flipped classroom (FC) **Control/Comparator**: Traditional class (TC)
**Outcomes**	Feedback questionnaires (students' perspectives) Pre‐ and posttests (MCQs)
**Notes**	Setting: Medical school of Sun Yat‐sen University, China Ethics approval: Obtained (IRB‐ZOC‐SYSU) Funding: Not mentioned Study period: Not mentioned

Risk of bias table

**Table:** 

Bias	Authors' judgement	Support for judgement
Random sequence generation (selection bias)	High risk	Quote: ‘Randomly allocated’. p. 2
Allocation concealment (selection bias)	High risk	Quote: ‘These participants were randomly allocated into either the flipped classroom group or the traditional lecture‐based classroom group’. p. 2
Blinding of participants and personnel (performance bias)	Low risk	Quote: ‘All students were unaware of their group assignments before the clerkship’. p. 2
Blinding of outcome assessment (detection bias)	Unclear risk	Quote: ‘the students assigned in the lecture‐based classroom group had the same access to the recorded lecture video and supplementary study materials as those in the flipped classroom group’. p. 4
Incomplete outcome data (attrition bias)	Low risk	No withdrawal
Selective reporting (reporting bias)	Low risk	Study flow diagram shown.
Other bias	Low risk	None
Confounding?/Free of contamination?	Unclear risk	
Baseline characteristics similar?	Unclear risk	
Baseline outcomes similar?	Unclear risk	
Intervention independent	Unclear risk	
Blinding	Unclear risk	
incomplete outcome data	Unclear risk	

Wang 2021

**Table:** 

**Methods**	
**Participants**	4th grade of a 6‐year Doctor of Dental Surgery (DDS) program in 3 consecutive academic years (2017,2018,2019) *N* = 144 recruited Analysed: 137 (FC, 70 vs. TC, 67) Male, *n* (%): Not mentioned Age in years (mean ± SD): Not mentioned
**Interventions**	Intervention: Flipped class (FC) Control/comparator: Traditional class/lecture‐based class (TC)
**Outcomes**	Academic performance: Individual and team readiness assurance tests (IRAT/TRAT) Student satisfaction
**Notes**	Setting: Tokyo Medical and Dental University (TMDU) Registry: Clinical Trials Registry (www.umin.ac.jp/) (UMIN000028111, registered in 01/09/2017) Ethics approval: Institutional Review Board of the Tokyo Medical and Dental University (TMDU) (approval no. D2017‐024 Funding: ‘The authors received no specific funding for this work’ Study period: October 2017 to February 2019

Risk of bias table

**Table:** 

Bias	Authors' judgement	Support for judgement
Random sequence generation (selection bias)	Low risk	A computerised random number ranging from 0 to 1 was generated for each participant.
Allocation concealment (selection bias)	Low risk	Concealed; assigned numbers <0.5 were allocated to the lecture group, while those with numbers >0.5 were allocated to the flipped group.
Blinding of participants and personnel (performance bias)	High risk	Not blined to participants
Blinding of outcome assessment (detection bias)	Low risk	Single‐assessor‐blinded trial.
Incomplete outcome data (attrition bias)	Low risk	ITT analysis
Selective reporting (reporting bias)	Low risk	As planned assessment (register protocol)
Other bias	Low risk	None
Confounding?/Free of contamination?	Unclear risk	
Baseline characteristics similar?	Unclear risk	
Baseline outcomes similar?	Unclear risk	
Intervention independent	Unclear risk	
Blinding	Unclear risk	
incomplete outcome data	Unclear risk	

Whelan 2015

**Table:** 

**Methods**	Cross‐sectional survey
**Participants**	Integrated anatomy education, streamlined anatomy curriculum Students who had completed the pre‐clerkship program (M2, M3, and M4) *N* = 478 (FC, 340 vs FAL 138) Age in years: Not mentioned Male, *n* (%): Not mentioned
**Interventions**	**Intervention**: Emphasised independent‐learning(flipped classroom) (EIL or FC) **Control/Comparator:** Facilitated active learning (FAL)
**Outcomes**	Quantitative (Likert‐style questions) qualitative data (independent thematic analysis of open‐ended commentary) Overall Response rate ‐ 47.1% (225 out of 478 possible students)
**Notes**	Setting: University of Ottawa, Canada Ethics approval: obtained an exempted status Funding: not mentioned Study period: 05/2014

Risk of bias table

**Table:** 

Bias	Authors' judgement	Support for judgement
Random sequence generation (selection bias)	Unclear risk	
Allocation concealment (selection bias)	Unclear risk	
Blinding of participants and personnel (performance bias)	Unclear risk	
Blinding of outcome assessment (detection bias)	Unclear risk	
Incomplete outcome data (attrition bias)	Unclear risk	
Selective reporting (reporting bias)	Unclear risk	
Other bias	Unclear risk	
Confounding?/Free of contamination?	Low risk	Quote ‘we also did not survey demographic information such as age, gender, or academic ability upon admission in survey respondents’. (p. 49)
Baseline characteristics similar?	Unclear risk	No demographic information
Baseline outcomes similar?	High risk	Quote: ‘the reliability (Cronbach's alpha) for the survey questions regarding perceptions of the laboratory learning environment (*α* = 0.74)‐‐many items related to that construct’. p. 49
Intervention independent	Unclear risk	Not stated
Blinding	Low risk	Quote: ‘coders worked independently using open coding to identify themes in the first 33% of the data set. Through’. p. 43
incomplete outcome data	Low risk	None

Whillier 2015

**Table:** 

**Methods**	Observational study
**Participants**	2nd year medical student in neuroanatomy unit *N* = 64 (FC, 29 vs. TC, 35) Male, n (%): not mentioned Age in years (mean ± SD): FC, (23.18 ± 10.41), TC (23.2 ± 6.3)
**Interventions**	**Intervention**: Flipped classroom in 2013 (FC) **Control/Comparator**: Traditional (regular) in 20 11 (TC)
**Outcomes**	Final course grades (standard numerical grade) Level of satisfaction (questionnaire)
**Notes**	Setting: Macquarie University, Australia Ethics Approval: obtained**—**(reference #: 5201100130; # 5201300691) Funding: Macquarie University Teaching Delivery Grant (4071/2054‐2013). Study period: 2011–2013

Risk of bias table

**Table:** 

Bias	Authors' judgement	Support for judgement
Random sequence generation (selection bias)	Unclear risk	
Allocation concealment (selection bias)	Unclear risk	
Blinding of participants and personnel (performance bias)	Unclear risk	
Blinding of outcome assessment (detection bias)	Unclear risk	
Incomplete outcome data (attrition bias)	Unclear risk	
Selective reporting (reporting bias)	Unclear risk	
Other bias	Unclear risk	
Confounding?/Free of contamination?	High risk	different tasks (Table 1) and duration for flipped classroom and the regular class Quote ‘questionnaire used, this has not been tested for reliability and validity’ (p. 132).
Baseline characteristics similar?	High risk	Quote: ‘Session was run over 6 weeks in 2011 and over only 5 weeks in 2013’ (p.129)
Baseline outcomes similar?	High risk	Quote: ‘The final SNG was used to compare the 2 cohorts. But this grade is dependent on the appropriateness and degree of similarity of the assessment tasks. The schedule of assessments was not the same for both cohorts’. (p. 132)
Intervention independent	Unclear risk	In 2011 vs. in 2013
Blinding	Unclear risk	Insufficient information
incomplete outcome data	Low risk	None

Wilson 2016

**Table:** 

**Methods**	Observational study
**Participants**	1st year–3rd year ‘over‐the‐counter(OTC) course pharmacotherapy’ Yeaf 1–>3 years ‘over‐the‐counter(OTC) course pharmacotherapy’ *N* = 189 (TBL, 102 vs. TC, 87) Male, *n* (%): Not mentioned Age in year, mean (±SD): TBL 27.2 (±4.7); TC, 25.1 ± 3
**Interventions**	**Intervention**: Team‐based learning in 2013 (TBL) **Control/Comparator**: Traditional didactic lecture (traditional class(TC)
**Outcomes**	Exam score for short‐term retention, 15 quizzes for long‐term retention Questionnaire (response rate 41% after excluding the incomplete questionnaire))
**Notes**	Setting: Wingate University School of Pharmacy, USA Ethical approval: Exempted Funding: declared no financial disclosure Study period: Spring 2014 and Spring 2015

Risk of bias table

**Table:** 

Bias	Authors' judgement	Support for judgement
Random sequence generation (selection bias)	Unclear risk	
Allocation concealment (selection bias)	Unclear risk	
Blinding of participants and personnel (performance bias)	Unclear risk	
Blinding of outcome assessment (detection bias)	Unclear risk	
Incomplete outcome data (attrition bias)	Unclear risk	
Selective reporting (reporting bias)	Unclear risk	
Other bias	Unclear risk	
Confounding?/Free of contamination?	Unclear risk	The same School; Quote: Information regarding the purpose of the survey was provided in the email announcement and survey;
Baseline characteristics similar?	Low risk	Students form same academic background, same content
Baseline outcomes similar?	Low risk	Same quizzes for long‐term retention, same questionnaire
Intervention independent	Unclear risk	Not stated
Blinding	Unclear risk	Quote: ‘The questionnaire was reviewed internally by a faculty research group before distribution’. p. 642
incomplete outcome data	Low risk	None

Wong 2014

**Table:** 

**Methods**	Case‐control design
**Participants**	1st year pharmacy students on the topic of cardiac arrhythmias *N* = 206 (FC, 101 vs. TC, 103) Male, *n* (%): FC, 33 (32.7); TC 31 (30.1) Age in years, mean (±SD): FC, 24.1 (0.3); TC, 24.7 (0.4) Exclusion criteria: Students not attended all 3 classes and/or were repeating any courses
**Interventions**	**Intervention**: Flipped classroom in 2012 (FC) **Control/comparison:** Traditional class in 2011 (TC)
**Outcomes**	Final examination scores (5–6 MCQs on cardiac arrhythmias) Students perception
**Notes**	Setting: California College of Pharmacy, Touro University, USA Ethical approval: Exempted Funding: Not mentioned Study period: Spring of 2012,

Risk of bias table

**Table:** 

Bias	Authors' judgement	Support for judgement
Random sequence generation (selection bias)	Unclear risk	
Allocation concealment (selection bias)	Unclear risk	
Blinding of participants and personnel (performance bias)	Unclear risk	
Blinding of outcome assessment (detection bias)	Unclear risk	
Incomplete outcome data (attrition bias)	Unclear risk	
Selective reporting (reporting bias)	Unclear risk	
Other bias	Unclear risk	
Confounding?/Free of contamination?	Low risk	Same background
Baseline characteristics similar?	Low risk	Quote: ‘The demographic characteristics of the intervention and control groups did not differ in mean age, gender, or undergraduate grade point average (GPA), though the intervention group had a slightly higher pharmacy GPA’. p. 3
Baseline outcomes similar?	Low risk	Same exam questions
Intervention independent	Unclear risk	Not stated
Blinding	Unclear risk	Not mentioned
incomplete outcome data	Low risk	None

Zheng 2020

**Table:** 

**Methods**	
**Participants**	
**Interventions**	
**Outcomes**	
**Notes**	Ethics approval: Institutional Review Board of Sun Yat‐sen University, China
	Study period: May–June, 2019

Risk of bias table

**Table:** 

Bias	Authors' judgement	Support for judgement
Random sequence generation (selection bias)	Unclear risk	Need more information Quote: ‘a randomized and single‐blind study’
Allocation concealment (selection bias)	Unclear risk	Need more information Quote: ‘The students assigned to the TLC group were required to finish the pre‐class exercises’
Blinding of participants and personnel (performance bias)	High risk	No blinding (single‐blind study)
Blinding of outcome assessment (detection bias)	Low risk	single‐blind study
Incomplete outcome data (attrition bias)	Low risk	All analysed
Selective reporting (reporting bias)	Unclear risk	Cannot be determined (no protocol)
Other bias	Low risk	None
Confounding?/Free of contamination?	Unclear risk	
Baseline characteristics similar?	Unclear risk	
Baseline outcomes similar?	Unclear risk	
Intervention independent	Unclear risk	
Blinding	Unclear risk	
incomplete outcome data	Unclear risk	

Zhu 2020

**Table:** 

**Methods**	Quasi‐experimental design
**Participants**	Mixed group, combined undergraduate nursing students, dental students and higher vocational medical students. *N* = 200, (FC, 100 vs. TC, 100) Nursing students: 30 vs. 31 Dental students: 32 vs. 31 Medical students: 38 vs. 31 Male, *n* (%); FC 36(36%) vs. TC, 32(32%) Age in years (mean ± SD): 21.2 ± 0.8 vs. 21.1 ± 0.9
**Interventions**	intervention: Flipped class (FC) Control/comparison: Lecture‐based learning/Traditional class(TC)
**Outcomes**	Academic performance (skill exam scores)
**Notes**	Setting: A university in China (no detailed description) Ethics approval: The region's ethical review board. Funding: Lishui University and University of Gävle Study period: April–June 2015 (nursing students), February–April 2017 (dental students) November 2017–January 2018 (medical students)

Risk of bias table

**Table:** 

Bias	Authors' judgement	Support for judgement
Random sequence generation (selection bias)	High risk	No randomisation, quasi‐experimental design
Allocation concealment (selection bias)	High risk	Not
Blinding of participants and personnel (performance bias)	High risk	Clinical lecturers (other than the researchers) completed the skill examination of students in both groups
Blinding of outcome assessment (detection bias)	High risk	Clinical lecturers (other than the researchers) completed the skill examination of students in both groups
Incomplete outcome data (attrition bias)	Low risk	All analyses
Selective reporting (reporting bias)	Unclear risk	Cannit be determined, no protocol found
Other bias	Low risk	None
Confounding?/Free of contamination?	Unclear risk	
Baseline characteristics similar?	Unclear risk	
Baseline outcomes similar?	Unclear risk	
Intervention independent	Unclear risk	
Blinding	Unclear risk	
incomplete outcome data	Unclear risk	

*Footnotes*

**Characteristics of excluded studies**

**Table:** 

Almanase 2018	
**Reason for exclusion**	Not undergraduate programme (year 4 master students)
Almodaires 2019	
**Reason for exclusion**	Not on health subject
Angshurekha 2020	
**Reason for exclusion**	Not included outcomes of interest
Appleyard 2019	
**Reason for exclusion**	A letter with no primary data
Armbruster 2009	
**Reason for exclusion**	Not a flipped class
Belfi 2015	
**Reason for exclusion**	Single group pre‐post test
Bonnes 2014	
**Reason for exclusion**	Not undergraduate students
Brown 2019	
**Reason for exclusion**	A mix of teachers and undergraduate students; no separate data for students
Burak 2017	
**Reason for exclusion**	Graduated medical students
Burden 2015	
**Reason for exclusion**	Not included outcomes of interest
Busebaia 2020	
**Reason for exclusion**	Only one group, no comparator
Chan 2020	
**Reason for exclusion**	No comparator group
Chen 2017	
**Reason for exclusion**	A review
Day 2018	
**Reason for exclusion**	Not undergraduate program, postgraduate students
Ding 2019	
**Reason for exclusion**	Study with graduate students
Dombrowski 2018	
**Reason for exclusion**	Not included outcomes of interest
El‐Banna 2017	
**Reason for exclusion**	Participants are already graduated students
Espada 2020	
**Reason for exclusion**	Not HPE (i.e., physical activity and sports science degree)
Fatima 2017	
**Reason for exclusion**	No comparison
Fatima 2019	
**Reason for exclusion**	A cross‐sectional survey, no matched comparator group
Galway 2014	
**Reason for exclusion**	Not undergraduate program, postgraduate students
Geist 2015	
**Reason for exclusion**	Difficult to extract data
Gomez‐Carrasco 2020	
**Reason for exclusion**	Not HPE
Hew 2018	
**Reason for exclusion**	A review, not a primary study
Hongsawong 2016	
**Reason for exclusion**	An abstract with insufficient data
Hopper 2020	
**Reason for exclusion**	Not included outcomes of interest; no data provided
Hurtubise 2015	
**Reason for exclusion**	No primary data
Katilya 2020	
**Reason for exclusion**	Not included outcomes of interest
Kim 2020	
**Reason for exclusion**	Not included a flipped class
King 2018	
**Reason for exclusion**	Postgraduate students
Kiviniemi 2014	
**Reason for exclusion**	Not undergraduate programme, a postgraduate programme (master level)
Koo 2016	
**Reason for exclusion**	Not undergraduate programme, a postgraduate program
Kugley 2016	
**Reason for exclusion**	Not flipped classroom, it's an information on systematic review
Kuhl 2019	
**Reason for exclusion**	Not included outcomes of interest
Lew 2016	
**Reason for exclusion**	Not included outcomes of interest
Libert 2016	
**Reason for exclusion**	Only one group, no comparator
Marchalot 2017	
**Reason for exclusion**	Residential programme
Martinelli 2017	
**Reason for exclusion**	Not undergraduate programme, a postgraduate programme
McLaughlin 2013	
**Reason for exclusion**	68% of participants were postgraduates
McLaughlin 2014	
**Reason for exclusion**	75% of participants were postgraduates
Moraros 2015	
**Reason for exclusion**	Not undergraduate programme, a postgraduate students
Njie‐Carr 2017	
**Reason for exclusion**	A review
Oliv́an 2019	
**Reason for exclusion**	Not undergraduate health programme (social workers)
Oudbier 2022	
**Reason for exclusion**	A review
Park 2015	
**Reason for exclusion**	Single group pre‐post test
Pierce 2012	
**Reason for exclusion**	Mix with undergraduate and master's degrees students; no separate data
Piercea 2012	
**Reason for exclusion**	Single group pre‐post test
Porcaro 2016	
**Reason for exclusion**	A mix sample of postgraduate and undergraduate; no separate data for undergraduate
Ramnanan 2017	
**Reason for exclusion**	A review
Rao 2001	
**Reason for exclusion**	Not a flipped class design
Rehman 2020	
**Reason for exclusion**	Not included outcomes of interest
Riddle 2017	
**Reason for exclusion**	Not undergraduate program
Roig‐Vila 2019	
**Reason for exclusion**	A review
Roy 2020	
**Reason for exclusion**	Difficult to extract data
Sait 2017	
**Reason for exclusion**	Only a letter with no primary data
Sandrone 2020	
**Reason for exclusion**	No comparator group
Sathapornsathid 2016	
**Reason for exclusion**	Insufficient data (abstract)
Schlairet 2014	
**Reason for exclusion**	No outcome data provided
Schneider 2019	
**Reason for exclusion**	No control/comparator group
Sheppard 2017	
**Reason for exclusion**	Only one group, no comparator group
Smith 2017	
**Reason for exclusion**	No outcome data provided
Sohn 2019	
**Reason for exclusion**	Only one group, no comparator group
Tsang 2016	
**Reason for exclusion**	No flipped class included
Tune 2013	
**Reason for exclusion**	Not undergraduate students (Graduate students)
Vadakedath 2019	
**Reason for exclusion**	Not flipped class included
Vavasseur 2020	
**Reason for exclusion**	Only one group, no comparator
Veeramani 2015	
**Reason for exclusion**	Only one group, no comparator
Wang 2020	
**Reason for exclusion**	Comparator is not a usual class
Watson 2015	
**Reason for exclusion**	Diffcult to extract data
Wozny 2018	
**Reason for exclusion**	Not health professional education (econometrics course)
Wu 2018	
**Reason for exclusion**	Not included outcomes of interest
Wu 2020	
**Reason for exclusion**	Only one group, no comparator
Young 2014	
**Reason for exclusion**	Not undergraduate programme
*Footnotes*	

## SUMMARY OF FINDINGS TABLES

Table 1 Summary of findings.

**Table:** 

[Flipped class compared with traditional class for undergraduuate students in health professional education programme
Patient or population: Undergraduate students
Settings: [Health professional education programme]
Intervention: [Flipped class]
Comparison: [Traditional lecture‐based class]

	Illustrative comparative risks* (95% CI)					
	Assumed risk	Corresponding risk				
Outcomes	[traditional lecture‐based class]	[Flipped class]	Relative effect (95% CI)	No of Participants (studies)	Quality of the evidence (GRADE)	Comments
*Academic performance*						
Any design (measured with exam score/Grade)			SMD 0.57 (0.25 higher to 0.9 higher)	7813 (44 studies)	⊕⊕◯◯ LOW^a,b,c^	
*Subgroup analysis*						
Academic performance (randomised controlled trial)			SMD 0.42 (0.18 higher to 0.65 higher)	1398 (11 studies)	⊕⊕◯◯ LOW^a,b,c^	
Academic performance (quasi‐experimental study)			SMD 0.52 (0.21 higher to 0.83 higher)	3894 (19 studies)	⊕⊕◯◯ LOW^a,b,c^	
Observational studies (two‐group cohort/case control design)			SMD 0.81 (0.23 lower to 1.85 higher)	2523 (14 studies)	⊕◯◯◯ Very LOW^a,b,c,d^	
Student satisfaction						
Student satisfaction (overall)			SMD 0.48 (0.15 higher to 0.82 higher)	1696 (8 studies)	⊕⊕◯◯ LOW^a,b,c^	
*Sensitivity analysis*						
Academic performance (measured with exam score/grade)			SMD 0.54 (0.24 higher to 0.85 higher)	5924 (33 studies)	⊕⊕◯◯ LOW^a,b,c^	
*The basis for the **assumed risk** (e.g., the median control group risk across studies) is provided in footnotes. The **corresponding risk** (and its 95% confidence interval) is based on the assumed risk in the comparison group and the **relative effect** of the intervention (and its 95% CI).						
CI: confidence interval; SMD: standard mean difference						
GRADE Working Group grades of evidence						
**High quality**: We are very confident that the true effect lies close to that of the estimate of the effect						
**Moderate quality**: We are moderately confident in the effect estimate: the true effect is likely to be close to the estimate of the effect, but there is a possibility that it is substantially different.						
**Low quality**: Our confidence in the effect estimate is limited: the true effect may be substantially different from the estimate of the effect.						
**Very low quality**: We have very little confidence in the effect estimate: the true effect is likely to be substantially different from the estimate of effect.						

High risk of selection bias.

Half of the studies are on opposite direction.

A wide 95% CI.

A wide 95% CI including a null value.

## DATA AND ANALYSES

**Table:** 

**Outcome or subgroup**	**Studies**	**Participants**	**Statistical method**	**Effect estimate**
**1 Academic performance (overall exam scores/grade)**				
1.1 Overall performance (exam scores/grade)	44	7813	Std. Mean Difference (IV, Random, 95% CI)	0.57 [0.25, 0.90]
**2 Students satisfaction**				
2.1 Student satisfaction (overall)	8	1696	Std. Mean Difference (IV, Random, 95% CI)	0.48 [0.15, 0.82]
**3 Academic performance (exam scores/grade) by design**				
3.1 Academic performance by RCT	11	1398	Std. Mean Difference (IV, Random, 95% CI)	0.42 [0.18, 0.65]
3.2 Academic performance by QES	19	3894	Std. Mean Difference (IV, Random, 95% CI)	0.52 [0.21, 0.83]
3.3 Academic performance by observational (two groups) design	14	2523	Std. Mean Difference (IV, Random, 95% CI)	0.81 [‐0.23, 1.85]
**4 Sensitivity analysis (after removal of studies with data imputation)**				
4.1 Overall performance (exam scores/grade)	33	5924	Std. Mean Difference (IV, Random, 95% CI)	0.54 [0.24, 0.85]

## SOURCES OF SUPPORT

**Internal sources**

- MAW and CN: College of Public Health, Medical and Veterinary Sciences, James Cook University, Townsville, Australia
  Faculty Development
- DKC and HHA: International Medical University, Kuala Lumpur, Malaysia

Faculty Development

**External sources**

- None, Other

No external support received.

## Supporting information

Supporting information.
