# Supplementary material for: The effects of flipped classrooms to improve learning outcomes in undergraduate health professional education: A systematic review
Source: Campbell Syst Rev. 2023 Jul 7;19(3):e1339. doi: 10.1002/cl2.1339 (PMC10326838; doi:10.1002/cl2.1339)
Supplement: Supplementary file 1 — Supporting information. [file CL2-19-e1339-s001.docx]

Appendices

## 1 Logic model of flipped class learning

| **RESOURCES** | **ACTIVITIES** | **OUTPUTS** | **OUTCOMES** |
| --- | --- | --- | --- |
| **Undergraduate education**  **Programme**  - (e.g. medicine, dentistry, pharmacy, nursing)  **Sub-speciality**  - (e.g. anatomy, pathology, epidemiology)  **Year/semester**  - (e.g. Year 1, Year 2, Semester 1, Semester 2)  **Resources for online learning**  - Design of the content  - Interactive tools  **Resources for face-to-face learning**  - Design of the content | **Context**  - single  - multifaceted interventions  **Execution**  - intensity (e.g. 1 hr, 2 hrs)  - integrated or stand-alone  **Delivery**  - Pure flipped classroom-learning  - blended learning  **Structure**  - space provided (e.g. classroom capacity) | **Observed products**  Students engage in  - ‘x’ number of hours during learning at their homes  - ‘x’ number of hours for face-to-face session  Students employ higher learning techniques through  interaction,  and group discussion, analysis and synthesis  Use of soft skills, namely: verbal and non-verbal communication | **Short term outcomes**  - enhanced academic performance  - enhanced learner satisfaction  - enhanced teacher satisfaction  - enhanced group activities / interaction  - enhanced motivation and application  **Mid-term outcomes**  - clearer and improved understanding of the contents  - improved higher learning  - enhanced ability to relate, interpret and applied the learned contents  **Long term outcomes**  - increased self-perceived knowledge  - improved confident on the subject matter  - improved course evaluation score |

## 2 Matrix indicating assessment tools in academic discplines

| **No.** | **Study, year** | **Country** | **Design** | | | **Programme** | | | | **School year** | | | **Discipline in Medicine** | | | | | | | **Main assessment tools** | | | |  |
| --- | --- | --- | --- | --- | --- | --- | --- | --- | --- | --- | --- | --- | --- | --- | --- | --- | --- | --- | --- | --- | --- | --- | --- | --- |
|  |  |  | **RCT** | **QES** | **Ob** | **M** | **P** | **N** | **O** | **1** | **2** | **≥3** | **A/P** | **Pat** | **Op** | **R/O** | **E/S** | **Ph** | **Other** | **MCQ** | **OSCE** | **Quiz** | **other** |  |
| 1 | Anderson 2017 | USA | √ |  |  |  | √ |  |  | √ |  |  |  |  |  |  |  |  |  |  | √ |  |  |  |
| 2 | Angadi 2019 | India |  | √ |  | √ |  |  |  |  | √ |  |  |  |  |  |  | √ |  | √ |  |  |  |  |
| 3 | Baris 2020 | Turkey |  | √ |  | √ |  |  |  |  | √ |  |  |  |  |  |  |  |  |  | √ |  |  |  |
| 4 | Bossaer 2016 | USA |  | √ |  |  | √ |  |  |  |  | √ |  |  |  | √ |  |  |  | √ |  |  |  |  |
| 5 | Boysen-Osborn 2016 | USA |  |  |  | √ |  |  |  |  |  | √ |  |  |  |  |  |  | √ | √ |  |  |  |  |
| 6 | Burak 2015 | Canada |  |  | √ | √ |  |  |  |  |  |  |  |  |  |  |  |  | √ |  |  |  | √Exam Performance |  |
| 7 | Chaudhuri 2019 | India |  |  | √ | √ |  |  |  | √ |  |  | √ |  |  |  |  |  |  | √ |  |  |  |  |
| 8 | Cheng 2016 | China |  |  | √ |  |  |  | √ |  |  |  |  |  |  |  |  |  |  | √ |  |  |  |  |
| 9 | Chiu 2018 | Taiwan | √ |  |  | √ |  |  |  |  |  | √ |  |  |  |  |  |  | √ |  |  |  | √ Mean score |  |
| 10 | Chu 2019 | Taiwan |  | √ |  |  |  | √ |  |  |  |  |  |  |  |  |  |  |  |  |  |  | √ EBP scale |  |
| 11 | Cotta 2016 | USA |  |  | √ |  | √ |  |  |  |  |  |  |  |  |  |  |  |  |  |  |  | √ Exam score |  |
| 12 | Dehghanzadeh 2020 | Iran |  | √ |  |  |  | √ |  |  | √ |  | √ |  |  |  |  |  |  |  |  |  | √Ricketts’ Critical Thinking Disposition Inventory |  |
| 13 | Dodiya 2019 | India | √ |  |  | √ |  |  |  |  |  |  |  |  |  |  |  |  |  |  |  |  |  |  |
| 14 | Evans 2016 | USA |  |  | √ | √ |  |  |  | √ |  |  |  |  |  |  | √ |  |  |  |  |  | √ Final exam scores |  |
| 15 | Fan 2020 | Taiwan |  | √ |  |  |  | √ |  |  | √ |  |  |  |  |  |  |  |  |  |  |  | √ |  |
| 16 | Gillispie 2016 | Australia |  |  | √ | √ |  |  |  |  |  | √ |  |  |  |  |  |  | √ | √ | √ |  |  |  |
| 17 | Grønlien 2021 | No |  | √ |  | √ |  | √ | √ |  |  |  |  |  |  |  |  |  |  |  |  |  |  |  |
| 18 | Harrington 2015 | USA | √ |  |  |  |  | √ |  | √ |  |  |  |  |  |  |  |  |  |  |  | √ |  |  |
| 19 | Heitz 2015 | USA | √ |  |  | √ |  |  |  |  |  | √ |  |  |  |  |  |  | √ | √ |  |  |  |  |
| 20 | Herrero 2020 | Spain |  | √ |  | √ |  |  |  |  |  | √ |  | √ |  |  |  |  |  | √ |  |  |  |  |
| 21 | Hu 2019 | China |  | √ |  | √ |  |  |  |  |  | √ |  |  |  |  |  |  | √ |  |  | √ |  |  |
| 22 | Huang 2020 | Taiwan |  | √ |  |  |  |  | √ |  |  |  |  |  |  |  |  |  |  |  |  |  | √ Fresno test scores |  |
| 23 | Isherwood 2019 | UK | √ |  |  |  |  |  | √ |  |  | √ |  |  |  |  |  |  | √ | √ |  |  |  |  |
| 24 | Kuhl 2017 | Germany | √ |  |  | √ |  |  |  | √ |  |  |  |  |  |  |  |  | √ |  |  |  | √ EOS score |  |
| 25 | Lin 2017 | China | √ |  |  | √ |  |  |  |  |  |  |  |  | √ |  |  |  |  |  |  |  | √ Exam score |  |
| 26 | Lucchetti 2018 | Brazil |  | √ |  | √ |  |  |  |  |  | √ |  |  |  |  |  |  | √ |  |  |  | √ |  |
| 27 | Missildine 2013 | USA |  | √ |  |  |  | √ |  |  |  |  |  |  |  |  |  |  |  |  |  |  | √ Exam score |  |
| 28 | Morton 2017 | USA |  |  | √ | √ |  |  |  | √ |  |  |  |  |  |  |  |  | √ |  |  |  | √ Final exam |  |
| 29 | O’Connor 2016 | USA |  |  | √ | √ |  |  |  |  |  | √ |  |  |  | √ |  |  |  |  |  |  | √ |  |
| 30 | Park 2018 | South Korea |  | √ |  |  |  | √ |  | √ |  |  |  |  |  |  |  |  | √ |  |  |  | √Mean score |  |
| 31 | Ren 2020 | China | √ |  |  |  | √ |  |  |  |  |  |  |  |  |  |  |  |  |  |  |  |  |  |
| 32 | Rui 2017 | China | √ |  |  | √ |  |  | √ |  |  |  |  |  |  |  |  |  | √ |  |  |  | √Test score |  |
| 33 | Sajid 2020 | Saudi Arabia |  | √ |  |  | √ |  |  |  |  |  |  |  |  |  |  |  |  |  |  |  |  |  |
| 34 | Sinclair-Bennett 2019 | USA |  | √ |  |  |  | √ |  |  |  |  |  |  |  |  |  |  | √ |  |  |  | √ |  |
| 35 | Stewart 2013 | USA |  |  | √ |  | √ |  |  |  |  | √ |  |  |  |  |  | √ |  | √ |  |  |  |  |
| 36 | Street 2015 | USA |  | √ |  | √ |  |  |  |  |  | √ | √ |  |  |  |  |  |  |  |  |  | √Exam score |  |
| 37 | Suda 2014 | USA |  | √ |  |  | √ |  |  |  |  | √ |  |  |  |  |  |  | √ | √ |  |  |  |  |
| 38 | Tang 2017 | China |  | √ |  | √ |  |  |  |  |  | √ |  |  | √ |  |  |  |  | √ |  |  |  |  |
| 39 | Wang 2021 | China | √ |  |  |  |  |  | √ |  |  |  |  |  |  |  |  |  |  |  |  |  |  |  |
| 40 | Whelan 2015 | Canada |  |  | √ | √ |  |  |  |  |  | √ | √ |  |  |  |  |  |  |  |  |  | √ |  |
| 41 | Whillier 2015 | Australia |  |  | √ | √ |  |  |  |  | √ |  | √ |  |  |  |  |  |  |  |  |  | √ |  |
| 42 | Wilson 2016 | USA |  |  | √ |  | √ |  |  | √ | √ | √ |  |  |  |  |  | √ |  |  |  | √ |  |  |
| 43 | Wong 2014 | USA |  |  | √ |  | √ |  |  | √ |  |  |  |  |  |  |  |  | √ cardiac arrythmia | √ |  |  |  |  |
| 44 | Zheng 2020 | China | √ |  |  | √ |  |  |  |  |  |  |  |  |  |  |  |  |  |  |  |  |  |  |
| 45 | Zhu 2020 | China |  | √ |  |  |  | √ |  |  |  |  |  |  |  |  |  |  |  |  |  |  |  |  |

M: Medicine; P: Pharmacy; N: Nursing; O: Others; A/P: Anatomy/Physiology, E/S: Epidemiology/Statistics, Pat: Patho/Pathophysiology, Ph: Pharmacology/Pharmacotherapy, Op: Ophthamaology, R/O: Radiology/oncology, RCT: Randomized controlled trial, QES: quasi-experimental design,Ob: Observational study, MCQ: Multiple choice question; EOS: End of semester; OSCE: Objective Structured Clinical Examination.

## 3 Search strategies

| **1) Electronic databases** |
| --- |
| **a) Ovid MEDLINE(R) and Epub Ahead of Print, In-Process, In-Data-Review & Other Non-Indexed Citations, Daily and Versions 1946 to April 28, 2022**   1. exp Education, Dental/ OR exp Education, Medical, Undergraduate/ OR exp education, medical/ OR exp Education, Nursing, Baccalaureate/ OR exp education, nursing/ OR exp education, pharmacy/ OR exp education, predental/ OR exp education, premedical/ OR exp education, professional/ OR exp education, public health professional/ 2. exp Health Occupations/ 3. exp education/ 4. 2 AND 3 5. 1 OR 4 6. (anatomy OR BSN OR chiropract* OR dental OR “health profession*“ OR Medical OR Nurs* OR pharmac* OR pre#dental OR pre#med* OR “public health”).tw. 7. (bachelor* OR class* OR course* OR educat* OR learn* OR instruct* OR professor* OR student* OR teach* OR train* OR undergrad*).tw. 8. 6 AND 7 9. 5 OR 8 10. ((“flip* the class*“) OR (flipped#classroom) OR (flip* ADJ10 class*) OR (flip* ADJ10 educat*) OR (flip* ADJ10 learn*) OR (flip* ADJ10 instruct*) OR (flip* ADJ10 teach*)).tw 11. ((“invert* the class*“) OR (inverted#classroom) OR (invert* ADJ10 class*) OR (invert* ADJ10 educat*) OR (invert* ADJ10 learn*) OR (invert* ADJ10 instruct*) OR (invert* ADJ10 teach*)).tw 12. 10 OR 11 13. 9 AND 12 |
| **b) EMBASE (Ovid)**   1. exp Health Education/ OR paramedical education/ Or exp Nursing education/ 2. exp Education/ OR exp Teacher/ OR exp Teaching/ OR exp Learning/ 3. exp Medical profession 4. 2 AND 3 5. 1 OR 4 6. (anatomy OR BSN OR chiropract* OR dental OR “health profession*“ OR Medical OR Nurs* OR pharmac* OR pre#dental OR pre#med* OR “public health”).ti,ab 7. (bachelor* OR class* OR course* OR educat* OR learn* OR instruct* OR professor* OR student* OR teach* OR train* OR undergrad*).ti,ab 8. 6 AND 7 9. 5 OR 8 10. ((“flip* the class*“) OR (flipped#classroom) OR (flip* ADJ10 class*) OR (flip* ADJ10 educat*) OR (flip* ADJ10 learn*) OR (flip* ADJ10 instruct*) OR (flip* ADJ10 teach*)).ti,ab 11. ((“invert* the class*“) OR (inverted#classroom) OR (invert* ADJ10 class*) OR (invert* ADJ10 educat*) OR (invert* ADJ10 learn*) OR (invert* ADJ10 instruct*) OR (invert* ADJ10 teach*)).ti,ab 12. (invertebrate* or flippase*).ti,ab 13. 12 NOT 10 14. 10 OR 11 15. 14 NOT 13 16. 9 AND 15 |
| **c} PubMed**  (((“Education, Dental”[Mesh] OR “Education, Medical, Undergraduate”[Mesh] OR “Education, Medical”[Mesh] OR “Education, Nursing, Baccalaureate”[Mesh] OR “Education, Nursing”[Mesh] OR “Education, Pharmacy”[Mesh] OR “Education, Predental”[Mesh] OR “Education, Premedical”[Mesh] OR “Education, Professional”[Mesh] OR “Education, Public Health Professional”[Mesh]) OR (“Health Occupations”[Mesh] AND “Education”[Mesh])) OR ((anatomy[tw] OR BSN[tw] OR chiropract*[tw] OR dental[tw] OR “health profession*“[tw] OR Medical[tw] OR Nurs*[tw] OR pharmac*[tw] OR predental[tw] OR pre-dental[tw] OR premed*[tw] OR pre-med[tw] OR “public health”[tw]) AND (bachelor*[tw] OR class*[tw] OR course*[tw] OR educat*[tw] OR learn*[tw] OR instruct*[tw] OR professor*[tw] OR student*[tw] OR teach*[tw] OR train*[tw] OR undergrad*[tw]))) AND (((flip*[tw] OR invert*[tw]) AND (class*[tw] OR learn*[tw] OR educat*[tw] OR teach*[tw] OR instruct*[tw])) NOT (invertebrate*[tw] or flippase*[tw])) |
| **d)** **Education Resources Information Centre (ERIC) via EBSCO**   1. TI (anatomy OR BSN OR chiropract* OR dental OR “health profession*“ OR Medical OR Nurs* OR pharmac* OR pre#dental OR pre#med* OR “public health”) 2. AB (anatomy OR BSN OR chiropract* OR dental OR “health profession*“ OR Medical OR Nurs* OR pharmac* OR pre#dental OR pre#med* OR “public health”) 3. S2 OR S3 4. TI (bachelor* OR class* OR course* OR educat* OR learn* OR instruct* OR professor* OR student* OR teach* OR train* OR undergrad*) 5. AB (bachelor* OR class* OR course* OR educat* OR learn* OR instruct* OR professor* OR student* OR teach* OR train* OR undergrad*) 6. DE “Undergraduate programs” OR DE “Academic degrees” OR DE “Cooperative education -- Universities & colleges” OR DE “Counseling in higher education” OR DE “Distance higher education” OR DE “Doctoral programs” OR DE “Graduate education” OR DE “Health education (Higher)” OR DE “Independent study -- Universities & colleges” OR DE “Multicultural education in universities & colleges” OR DE “Postdoctoral programs” OR DE “Professional education” OR DE “Science education (Higher)” OR DE “Undergraduate programs” OR DE “University extension” OR DE “Women’s colleges” OR DE Associate degree education” OR DE “Bachelor’s degree” OR DE “College students” OR DE “Doctoral programs” OR DE “Universities & colleges” OR DE Undergraduates OR DE “University faculty” OR DE College Teachers” 7. S5 OR S6 OR S7 8. S4 AND S8 9. S1 OR S9 10. DE “Flipped classrooms” 11. TI ((“flip* the class*“) OR (flipped#classroom) OR (flip* N10 class*) OR (flip* N10 educat*) OR (flip* N10 learn*) OR (flip* N10 instruct*) OR (flip* N10 teach*)) 12. AB ((“flip* the class*“) OR (flipped#classroom) OR (flip* N10 class*) OR (flip* N10 educat*) OR (flip* N10 learn*) OR (flip* N10 instruct*) OR (flip* N10 teach*)) 13. TI ((“invert* the class*“) OR (inverted#classroom) OR (invert* N10 class*) OR (invert* N10 educat*) OR (invert* N10 learn*) OR (invert* N10 instruct*) OR (invert* N10 teach*)) 14. AB ((“invert* the class*“) OR (inverted#classroom) OR (invert* N10 class*) OR (invert* N10 educat*) OR (invert* N10 learn*) OR (invert* N10 instruct*) OR (invert* N10 teach*)) 15. S11 OR S12 OR S13 OR S14 OR S15 16. S10 AND S16 |
| **e) CENTRAL**  (flip OR flipped OR flipping OR inverted OR inverting) AND (classroom OR class OR classes OR educat* OR teach* OR learn*) |
| **f) SCOPUS**  (((TITLE-ABS-KEY((bachelor* OR class* OR course* OR educat* OR learn* OR instruct* OR professor* OR student* OR teach* OR train* OR undergrad*)) AND TITLE-ABS-KEY((anatomy OR BSN OR chiropract* OR dental OR “health profession*“ OR Medical OR Nurs* OR pharmac* OR pre-dental OR predental OR pre-med* OR premed OR “public health”)))) AND ((TITLE-ABS-KEY(((“flip* the class*“) OR ( flipped AND classroom) OR ( flip* W/9 class*) OR ( flip* W/9 educat*) OR ( flip* W/9 learn*) OR ( flip* W/9 instruct*) OR ( flip* W/9 teach*))) OR TITLE-ABS-KEY(( (“invert* the class*“) OR (inverted classroom) OR (invert* W/9 class*) OR (invert* W/9 educat*) OR (invert* W/9 learn*) OR (invert* W/9 instruct*) OR (invert* W/9 teach*)))))) |
| **g) Best Evidence Medical Education**  Site searched  flip* OR invert* site:bemecollaboration.org  flip* OR invert* site:amee.org |
| **h) APA PsycINFO**   1. exp Nursing Education/ or exp Medical Education/ or exp Nursing Students/ 2. exp Higher Education/ OR exp Education/ OR exp Classrooms/ OR exp Teaching/ OR exp College Students/ OR exp Graduate Students/ OR exp Postgraduate Students/ 3. (bachelor* OR class* OR course* OR educat* OR learn* OR instruct* OR professor* OR student* OR teach* OR train* OR undergrad*).tw. 4. (anatomy OR BSN OR chiropract* OR dental OR “health profession*“ OR Medical OR Nurs* OR pharmac* OR pre?dental OR pre?med* OR “public health”).tw. 5. 2 OR 3 6. 4 AND 5 7. 1 OR 6 8. ((“flip* the class*“) OR (flipped?classroom) OR (flip* ADJ10 class*) OR (flip* ADJ10 educat*) OR (flip* ADJ10 learn*) OR (flip* ADJ10 instruct*) OR (flip* ADJ10 teach*)).tw 9. ((“invert* the class*“) OR (inverted?classroom) OR (invert* ADJ10 class*) OR (invert* ADJ10 educat*) OR (invert* ADJ10 learn*) OR (invert* ADJ10 instruct*) OR (invert* ADJ10 teach*)).tw 10. 8 OR 9 11. 7 AND 10 |
| **i) Web of Science Core Collection**   1. TOPIC:((bachelor* OR class* OR course* OR educat* OR learn* OR instruct* OR professor* OR student* OR teach* OR train* OR undergrad*)) 2. TOPIC: ((anatomy OR BSN OR chiropract* OR dental OR “health profession*“ OR Medical OR Nurs* OR pharmac* OR pre-dental OR predental OR pre-med* OR premed OR “public health”)) 3. 1 AND 2 4. TOPIC:((((“flip* the class*“) OR (flipped classroom) OR (flip* NEAR/10 class*) OR (flip* NEAR/10 educat*) OR (flip* NEAR/10 learn*) OR (flip* NEAR/10 instruct*) OR (flip* NEAR/10 teach*))) NOT( flippase*)) 5. TOPIC:((((“invert* the class*“) OR (inverted classroom) OR (invert* NEAR/10 class*) OR (invert* NEAR/10 educat*) OR (invert* NEAR/10 learn*) OR (invert* NEAR/10 instruct*) OR (invert* NEAR/10 teach*))) NOT (invertebrate*)) 6. 4 OR 5 7. 3 AND 6 |
| **j) Google Scholar**  (Health OR medical) AND (“flip* class*“ OR “invert* class” OR “flip* learn*“ OR “invert* learn” OR “flip* the class*“ OR “invert* the class*“) followed by eliminating patents |
| **2) Research Registers and Websites** |
| **a) Cochrane Library**  (flip OR flipped OR flipping OR inverted OR inverting) AND (classroom OR class OR classes OR educat* OR teach* OR learn*) |
| **b) Campbell Library**  flip OR flipped OR flipping OR inverted OR inverting |
| **c) Database of Abstracts of Reviews of Effectiveness**  (classroom OR class OR classes OR educat* OR teach* OR learn*):TI AND (flip OR flipped OR flipping OR inverted OR inverting):TI |
| **d) System for Information on Grey Literature**  title:(((flip* OR invert*) AND (class OR classes OR classroom OR teach* OR educat* OR learn*) AND (health* OR medical*)) NOT (invertebrate OR invertase OR “inverted-u”)) OR abstract:(((flip* OR invert*) AND (class OR classes OR classroom OR teach* OR educat* OR learn*) AND (health* OR medical*)) NOT (invertebrate OR invertase OR “inverted-u”)) |
| **e) Evidence for Policy Practice Information and Coordinating Centre (EPPI-Centre**)  (flip* OR invert*) AND (class* OR teach* OR educat* OR learn*) AND (health* OR medical*) |
| **f) Applied Social Sciences Index and Abstracts (ASSIA)**  1. ti((bachelor* OR class* OR course* OR educat* OR learn* OR instruct* OR professor* OR student* OR teach* OR train* OR undergrad*)) OR ab((bachelor* OR class* OR course* OR educat* OR learn* OR instruct* OR professor* OR student* OR teach* OR train* OR undergrad*))  2. ti((anatomy OR BSN OR chiropract* OR dental OR “health profession*“ OR Medical OR Nurs* OR pharmac* OR pre-dental OR predental OR pre-med* OR premed OR health OR “public health”)) OR ab((anatomy OR BSN OR chiropract* OR dental OR “health profession*“ OR Medical OR Nurs* OR pharmac* OR pre-dental OR predental OR pre-med* OR premed OR health OR “public health”))  3. 1 AND 2  4. ti((“flip* the class*“) OR (flipped#classroom) OR (flip* NEAR/10 class*) OR (flip* NEAR/10 educat*) OR (flip* NEAR/10 learn*) OR (flip* NEAR/10 instruct*) OR (flip* NEAR/10 teach*)) OR ab((“flip* the class*“) OR (flipped#classroom) OR (flip* NEAR/10 class*) OR (flip* NEAR/10 educat*) OR (flip* NEAR/10 learn*) OR (flip* NEAR/10 instruct*) OR (flip* NEAR/10 teach*))  5. ti((“invert* the class*“) OR (inverted#classroom) OR (invert* NEAR/10 class*) OR (invert* NEAR/10 educat*) OR (invert* NEAR/10 learn*) OR (invert* NEAR/10 instruct*) OR (invert* NEAR/10 teach*)) OR ab((“invert* the class*“) OR (inverted#classroom) OR (invert* NEAR/10 class*) OR (invert* NEAR/10 educat*) OR (invert* NEAR/10 learn*) OR (invert* NEAR/10 instruct*) OR (invert* NEAR/10 teach*))  6. 4 OR 5  7. 3 AND 6 |
| **3) Dissertations and theses databases** |
| **Proquest Global Dissertations & Theses**   1. ti((bachelor* OR class* OR course* OR educat* OR learn* OR instruct* OR professor* OR student* OR teach* OR train* OR undergrad*)) OR ab((bachelor* OR class* OR course* OR educat* OR learn* OR instruct* OR professor* OR student* OR teach* OR train* OR undergrad*)) 2. ti((anatomy OR BSN OR chiropract* OR dental OR “health profession*“ OR Medical OR Nurs* OR pharmac* OR pre-dental OR predental OR pre-med* OR premed OR “public health”)) OR ab((anatomy OR BSN OR chiropract* OR dental OR “health profession*“ OR Medical OR Nurs* OR pharmac* OR pre-dental OR predental OR pre-med* OR premed OR “public health”)) 3. 1 AND 2 4. ti((“flip* the class*“) OR (flipped#classroom) OR (flip* NEAR/10 class*) OR (flip* NEAR/10 educat*) OR (flip* NEAR/10 learn*) OR (flip* NEAR/10 instruct*) OR (flip* NEAR/10 teach*)) OR ab((“flip* the class*“) OR (flipped#classroom) OR (flip* NEAR/10 class*) OR (flip* NEAR/10 educat*) OR (flip* NEAR/10 learn*) OR (flip* NEAR/10 instruct*) OR (flip* NEAR/10 teach*)) 5. ti((“invert* the class*“) OR (inverted#classroom) OR (invert* NEAR/10 class*) OR (invert* NEAR/10 educat*) OR (invert* NEAR/10 learn*) OR (invert* NEAR/10 instruct*) OR (invert* NEAR/10 teach*)) OR ab((“invert* the class*“) OR (inverted#classroom) OR (invert* NEAR/10 class*) OR (invert* NEAR/10 educat*) OR (invert* NEAR/10 learn*) OR (invert* NEAR/10 instruct*) OR (invert* NEAR/10 teach*)) 6. 4 OR 5 7. ti(invertebrate* OR flippase*) OR ab(invertebrate* OR flippase*) 8. 6 NOT 7 9. 3 AND 8 |
| **Index to Theses in Great Britain and Ireland (www.theses.com/)**  (flip* OR invert*) AND (class OR classes OR classroom OR teach* OR educat* OR learn*) AND (health* OR medical*) |
| **Theses Canada (www.collectionscanada.gc.ca/thesescanada/)**  Any keyword: (flip* OR invert*) AND (class OR classes OR classroom OR teach* OR educat* OR learn*) AND (health* OR medical*) |
| **Networked Digital Library of Theses and Dissertations (http://www.ndltd.org/)**  description:(flip OR flipped OR flipping OR flips OR invert OR inverts OR inverted OR inverting) AND (class OR classes OR classroom OR teach* OR educat* OR learn*) AND (health* OR medical*) |
| **4) Regional bibliographic databases** |
| **Australian Education Index (https://opac.acer.edu.au/edresearch/index.html)**  (flip* OR invert*) AND (class OR classes OR classroom OR teach* OR educat* OR learn*) AND (health* OR medical*) |
| **British Education Index (EBSCO)**  1. TI (anatomy OR BSN OR chiropract* OR dental OR “health profession*“ OR Medical OR Nurs* OR pharmac* OR pre#dental OR pre#med* OR “public health”) OR AB (anatomy OR BSN OR chiropract* OR dental OR “health profession*“ OR Medical OR Nurs* OR pharmac* OR pre#dental OR pre#med* OR “public health”)  2. TI (bachelor* OR class* OR course* OR educat* OR learn* OR instruct* OR professor* OR student* OR teach* OR train* OR undergrad*) OR AB (bachelor* OR class* OR course* OR educat* OR learn* OR instruct* OR professor* OR student* OR teach* OR train* OR undergrad*)  3. S1 AND S2  4. TI ((“flip* the class*“) OR (flipped#classroom) OR (flip* N10 class*) OR (flip* N10 educat*) OR (flip* N10 learn*) OR (flip* N10 instruct*) OR (flip* N10 teach*)) OR AB ((“flip* the class*“) OR (flipped#classroom) OR (flip* N10 class*) OR (flip* N10 educat*) OR (flip* N10 learn*) OR (flip* N10 instruct*) OR (flip* N10 teach*))  5. TI ((“invert* the class*“) OR (inverted#classroom) OR (invert* N10 class*) OR (invert* N10 educat*) OR (invert* N10 learn*) OR (invert* N10 instruct*) OR (invert* N10 teach*)) OR AB ((“invert* the class*“) OR (inverted#classroom) OR (invert* N10 class*) OR (invert* N10 educat*) OR (invert* N10 learn*) OR (invert* N10 instruct*) OR (invert* N10 teach*))  6. S4 OR S5  7. S3 AND S6 |
| **Canadian Business & Current Affairs Database (Proquest)**  1. ti((bachelor* OR class* OR course* OR educat* OR learn* OR instruct* OR professor* OR student* OR teach* OR train* OR undergrad*)) OR ab((bachelor* OR class* OR course* OR educat* OR learn* OR instruct* OR professor* OR student* OR teach* OR train* OR undergrad*))  2. ti((anatomy OR BSN OR chiropract* OR dental OR “health profession*“ OR Medical OR Nurs* OR pharmac* OR pre-dental OR predental OR pre-med* OR premed OR “public health”)) OR ab((anatomy OR BSN OR chiropract* OR dental OR “health profession*“ OR Medical OR Nurs* OR pharmac* OR pre-dental OR predental OR pre-med* OR premed OR “public health”))  3. 1 AND 2  4. ti((“flip* the class*“) OR (flipped#classroom) OR (flip* NEAR/10 class*) OR (flip* NEAR/10 educat*) OR (flip* NEAR/10 learn*) OR (flip* NEAR/10 instruct*) OR (flip* NEAR/10 teach*)) OR ab((“flip* the class*“) OR (flipped#classroom) OR (flip* NEAR/10 class*) OR (flip* NEAR/10 educat*) OR (flip* NEAR/10 learn*) OR (flip* NEAR/10 instruct*) OR (flip* NEAR/10 teach*))  5. ti((“invert* the class*“) OR (inverted#classroom) OR (invert* NEAR/10 class*) OR (invert* NEAR/10 educat*) OR (invert* NEAR/10 learn*) OR (invert* NEAR/10 instruct*) OR (invert* NEAR/10 teach*)) OR ab((“invert* the class*“) OR (inverted#classroom) OR (invert* NEAR/10 class*) OR (invert* NEAR/10 educat*) OR (invert* NEAR/10 learn*) OR (invert* NEAR/10 instruct*) OR (invert* NEAR/10 teach*))  6. 4 OR 5  7. ti(invertebrate* OR flippase*) OR ab(invertebrate* OR flippase*)  8. 6 NOT 7  9. 3 AND 8 |
| **Canadian Research Index (Proquest)**  1. ti((bachelor* OR class* OR course* OR educat* OR learn* OR instruct* OR professor* OR student* OR teach* OR train* OR undergrad*)) OR ab((bachelor* OR class* OR course* OR educat* OR learn* OR instruct* OR professor* OR student* OR teach* OR train* OR undergrad*))  2. ti((anatomy OR BSN OR chiropract* OR dental OR “health profession*“ OR Medical OR Nurs* OR pharmac* OR pre-dental OR predental OR pre-med* OR premed OR “public health”)) OR ab((anatomy OR BSN OR chiropract* OR dental OR “health profession*“ OR Medical OR Nurs* OR pharmac* OR pre-dental OR predental OR pre-med* OR premed OR “public health”))  3. 1 AND 2  4. ti((“flip* the class*“) OR (flipped#classroom) OR (flip* NEAR/10 class*) OR (flip* NEAR/10 educat*) OR (flip* NEAR/10 learn*) OR (flip* NEAR/10 instruct*) OR (flip* NEAR/10 teach*)) OR ab((“flip* the class*“) OR (flipped#classroom) OR (flip* NEAR/10 class*) OR (flip* NEAR/10 educat*) OR (flip* NEAR/10 learn*) OR (flip* NEAR/10 instruct*) OR (flip* NEAR/10 teach*))  5. ti((“invert* the class*“) OR (inverted#classroom) OR (invert* NEAR/10 class*) OR (invert* NEAR/10 educat*) OR (invert* NEAR/10 learn*) OR (invert* NEAR/10 instruct*) OR (invert* NEAR/10 teach*)) OR ab((“invert* the class*“) OR (inverted#classroom) OR (invert* NEAR/10 class*) OR (invert* NEAR/10 educat*) OR (invert* NEAR/10 learn*) OR (invert* NEAR/10 instruct*) OR (invert* NEAR/10 teach*))  6. 4 OR 5  7. ti(invertebrate* OR flippase*) OR ab(invertebrate* OR flippase*)  8. 6 NOT 7  9. 3 AND 8 |
| **LILACS (https://lilacs.bvsalud.org/en/)**  1. ti((bachelor* OR class* OR course* OR educat* OR learn* OR instruct* OR professor* OR student* OR teach* OR train* OR undergrad*)) OR ab((bachelor* OR class* OR course* OR educat* OR learn* OR instruct* OR professor* OR student* OR teach* OR train* OR undergrad*))  2. ti((anatomy OR BSN OR chiropract* OR dental OR “health profession*“ OR Medical OR Nurs* OR pharmac* OR pre-dental OR predental OR pre-med* OR premed OR “public health”)) OR ab((anatomy OR BSN OR chiropract* OR dental OR “health profession*“ OR Medical OR Nurs* OR pharmac* OR pre-dental OR predental OR pre-med* OR premed OR “public health”))  3. 1 AND 2  4. ti((“flip* the class*“) OR (flipped#classroom) OR (flip* NEAR/10 class*) OR (flip* NEAR/10 educat*) OR (flip* NEAR/10 learn*) OR (flip* NEAR/10 instruct*) OR (flip* NEAR/10 teach*)) OR ab((“flip* the class*“) OR (flipped#classroom) OR (flip* NEAR/10 class*) OR (flip* NEAR/10 educat*) OR (flip* NEAR/10 learn*) OR (flip* NEAR/10 instruct*) OR (flip* NEAR/10 teach*))  5. ti((“invert* the class*“) OR (inverted#classroom) OR (invert* NEAR/10 class*) OR (invert* NEAR/10 educat*) OR (invert* NEAR/10 learn*) OR (invert* NEAR/10 instruct*) OR (invert* NEAR/10 teach*)) OR ab((“invert* the class*“) OR (inverted#classroom) OR (invert* NEAR/10 class*) OR (invert* NEAR/10 educat*) OR (invert* NEAR/10 learn*) OR (invert* NEAR/10 instruct*) OR (invert* NEAR/10 teach*))  6. 4 OR 5  7. ti(invertebrate* OR flippase*) OR ab(invertebrate* OR flippase*)  8. 6 NOT 7  9. 3 AND 8 |
| **5) Full-text journals available electronically** |
| **BioMedCentral (www.biomedcentral.com)**  site search (e.g., (invert OR inverted OR inverting OR inverts OR flipping OR flip OR flips OR flipped) AND (teach* OR classroom* OR instruct*) |
| **Public Library of Science (PLoS) (www.plos.org)**  (flip* OR invert*) AND (class OR classes OR classroom OR teach* OR educat* OR learn*) AND (health* OR medical*) |
| **PubMedCentral (PMC) (www.pubmedcentral.nih.gov/)**  1. Search (((anatomy[Abstract] OR BSN[Abstract] OR chiropract*[Abstract] OR dental[Abstract] OR “health profession*“[Abstract] OR Medical[Abstract] OR Nurs*[Abstract] OR pharmac*[Abstract] OR pre#dental[Abstract] OR pre#med*[Abstract] OR “public health”)[Abstract])) OR ((anatomy[Title] OR BSN[Title] OR chiropract*[Title] OR dental[Title] OR “health profession*“[Title] OR Medical[Title] OR Nurs*[Title] OR pharmac*[Title] OR pre#dental[Title] OR pre#med*[Title] OR “public health”)[Title])  2. Search (((bachelor*[Abstract] OR class*[Abstract] OR course*[Abstract] OR educat*[Abstract] OR learn*[Abstract] OR instruct*[Abstract] OR professor*[Abstract] OR student*[Abstract] OR teach*[Abstract] OR train*[Abstract] OR undergrad*)[Abstract])) OR ((bachelor*[Title] OR class*[Title] OR course*[Title] OR educat*[Title] OR learn*[Title] OR instruct*[Title] OR professor*[Title] OR student*[Title] OR teach*[Title] OR train*[Title] OR undergrad*)[Title])  3. Search ((((“flip* the class*“)[Abstract] OR (flipped#classroom)[Abstract] OR (flip* class*)[Abstract] OR (flip* educat*)[Abstract] OR (flip* learn*)[Abstract] OR (flip* instruct*)[Abstract] OR (flip* teach*))[Abstract] OR (“invert* the class*“)[Abstract] OR (inverted#classroom)[Abstract] OR (invert* class*)[Abstract] OR (invert* educat*)[Abstract] OR (invert* learn*)[Abstract] OR (invert* instruct*)[Abstract] OR (invert* teach*))[Abstract])) OR (((“flip* the class*“)[Title] OR (flipped#classroom)[Title] OR (flip* class*)[Title] OR (flip* educat*)[Title] OR (flip* learn*)[Title] OR (flip* instruct*)[Title] OR (flip* teach*))[Title] OR (“invert* the class*“)[Title] OR (inverted#classroom)[Title] OR (invert* class*)[Title] OR (invert* educat*)[Title] OR (invert* learn*)[Title] OR (invert* instruct*)[Title] OR (invert* teach*))[Title])  4. #1 AND #2 AND #3 |
| **Directory of Open Access Journals (DOAJ) (www.doaj.org)**  flipp* class* (health OR medical) |

## 4 Facilitators (enabling factors) and barriers

| **Study** | **Barriers** | **Facilitators** |
| --- | --- | --- |
| Angadi 2019 | Internet accessibility | - |
| Baris 2020 | A lack of training materials | Students usually adopt a passive role, given a friendly atmosphere. |
| Belfi 2015a | Internet is essential |  |
| Bossaer 2016 | Did not have enough time to listen lectures before coming to class | Could rewind and relisten |
| Chaudhuri 2019 | Study materials provided to them all these methods were not at all fruitful. |  |
| Chiu 2018 | - | All programme facilitators are qualified by by Taiwan Evidence-Based Medicine Association. |
| Zhu 2020 | To spend a great deal of time to be well prepared before the flipped classroom. | Improve abilities (self-regulated learning, critical thinking, team cooperation, communication, performance, presentation, and so on) |
